# Changes in the Profile of Fecal Microbiota and Metabolites as Well as Serum Metabolites and Proteome After Dietary Inulin Supplementation in Dairy Cows With Subclinical Mastitis

**DOI:** 10.3389/fmicb.2022.809139

**Published:** 2022-04-04

**Authors:** Yue Wang, Xuemei Nan, Yiguang Zhao, Linshu Jiang, Hui Wang, Fan Zhang, Dengke Hua, Jun Liu, Liang Yang, Junhu Yao, Benhai Xiong

**Affiliations:** ^1^State Key Laboratory of Animal Nutrition, Institute of Animal Science, Chinese Academy of Agricultural Sciences, Beijing, China; ^2^College of Animal Science and Technology, Northwest A&F University, Yangling, China; ^3^Beijing Key Laboratory for Dairy Cow Nutrition, Beijing University of Agriculture, Beijing, China; ^4^Langfang Academy of Agriculture and Forestry, Langfang, China

**Keywords:** subclinical mastitis, inulin, fecal microbiota, metabolomics, serum proteome, dairy cows

## Abstract

The occurrence and development of mastitis is linked to dysbiostic gastrointestinal microbiota. Inulin is a dietary prebiotic that improves the profile of intestinal flora. Our previous study showed that inulin supplementation could improve the ruminal microbes of subclinical mastitis (SCM) cows. The current study attempted to further investigate the response of hindgut (fecal) microbiome and metabolites, serum metabolism, and protein expression to inulin in the in SCM cows. Different levels of inulin (0, 100, 200, 300, and 400 g/day per cow) were supplemented in SCM cows. Compared with control group, *Bacteroides* and *Bifidobacteria* were increased, and *Paeniclostridium*, *Ruminococcaceae*, *Coprococcus*, and *Clostridia* were decreased in the feces of inulin groups, and accompanied with elevated propionate and butyrate concentrations, while secondary bile acid (SBA) metabolites were increased and proinflammatory lipid oxidation products were dropped in both feces and serum. In serum, inulin intake suppressed the levels of triglyceride (TG) and low-density lipoprotein (LDL). Serum proteome analysis found that CD44 antigen, phosphatidylinositol-glycan-specific phospholipase D, apolipoprotein A-II, and superoxide dismutase [Cu-Zn] were upregulated, while cathelicidin-1, haptoglobin, serpin A3, inter-alpha-trypsin inhibitor heavy chain H4 were downregulated in inulin groups. These findings suggested further evidence for inulin supplementation in amelioration of inflammatory symptoms in SCM cows, which might provide alternative treatment for mastitis.

## Graphical Abstract

Effects of dietary inulin supplementation on fecal microorganisms and metabolites, as well as serum metabolites and proteins in dairy cows with subclinical mastitis.

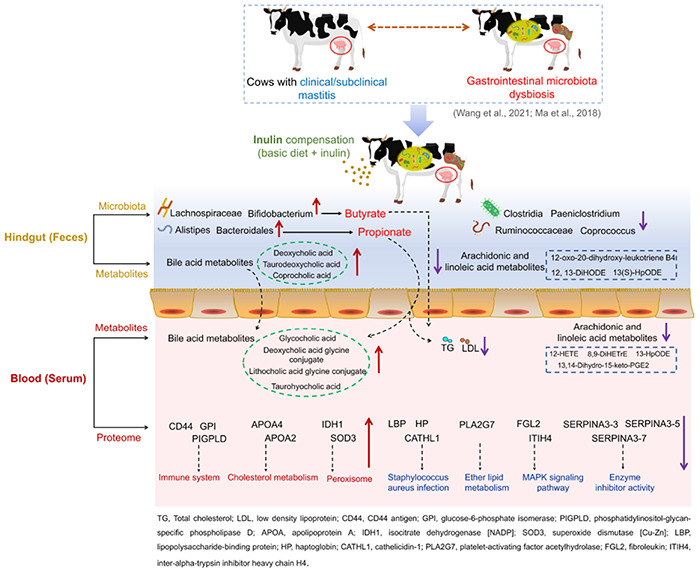

## Introduction

High prevalence of subclinical mastitis (SCM) is a major threat to dairy farms. If not observed and treated in time, SCM is prone to developing into clinical mastitis (CM) (Kasozi et al., 2014), resulting in serious loss of milk yield, quality, cow health, and treatment costs (Hogeveen et al., 2011). Although without obvious clinical symptoms in the udder, the changes in the milk of cows suffering from SCM do occur, including increased milk somatic cell counts (SCC, SCC > 200,000 cells/ml), and reduced milk yield and milk composition (Zhang et al., 2015). Previous studies believed that mastitis is a local inflammation of the mammary gland caused by pathogen infection (Zadoks et al., 2011), and most treatments were merely localized to the udder, such as local injection of antibiotics or antimicrobial peptides (Barlow, 2011). Therefore, mastitis is also the main reason for antibiotic usage on continued basis and in large quantities (Barlow, 2011). More recently, growing studies confirmed the correlation between the profiling of gastrointestinal microbiota, and the occurrence and development of mastitis (Ma et al., 2018a; Zhong et al., 2018; Hu et al., 2020; Wang et al., 2021b). These latest findings may provide new possibilities for describing pathogenesis and control of mastitis. It is reported that transplanting the feces of mastitic cows into sterile mice caused mastitis symptoms in mice and inflammation in the serum [increased interferon (IFN)-γ, interleukins (IL)-17, and endotoxin] and colon (increased IL-1β), which did not occur when transplanting healthy cows’ feces. However, supplementation with probiotics could relieve the mastitic symptoms in mice (Ma et al., 2018a). Another study compared the physiological changes between normal mice and antibiotic-mediated gut microbiota-dysbiosis mice after *Staphylococcus aureus* induced mastitis. Results showed increased blood–milk barrier permeability and lower fecal short-chain fatty acid (SCFA) concentration in the gut microbiota-dysbiosis mastitic mice. Administration of sodium propionate and sodium butyrate could reduce the permeability of the blood–milk barrier and alleviate inflammation (Hu et al., 2020). Similarly, upon *E. coli* stimulation, gut microbiota-dysbiostic mice had more apparent mastitis characteristics compared with control mice, including increased myeloperoxidase activity, tumor necrosis factor (TNF)-α, and interleukin (IL)-1β concentrations in the mammary gland tissue (Zhao et al., 2021). The above studies suggested that disturbance in the gut microbiota community may cause or aggravate mastitis. Our previous research found significantly decreased SCFA (acetate, propionate, and butyrate) concentration and elevated inflammation-related microbes, such as *Moraxella*, *Neisseriaceae*, and *Enterorhabdus*, and proinflammatory metabolism in the rumen of SCM and CM cows compared with healthy cows (Wang et al., 2021b). Similar studies showed lower propionate concentration and *Succinivibrionaceae* abundance in the rumen of high-somatic cell count (SCC) (>1,000,000 somatic cells/ml) cows compared with low-SCC (< 200,000 somatic cells/ml) cows (Zhong et al., 2018). These findings indicated changes in SCFA concentration and profiles of microbes and metabolites in the rumen during mastitis. Gastrointestinal microbiota dysbiosis may cause harmful intestinal metabolites, such as LPS, to enter the mammary gland (Hu et al., 2020).

Commensal microbiota in the gastrointestinal tract participates in nutrient digestion and absorption, specific immune response, and defense of pathogen colonization. Conversely, gut microbiota dysbiosis may be triggered in systemic inflammatory diseases in the host (Kamada et al., 2013). Crosstalk of gastrointestinal microbiota (Berg, 1995) and the entero-mammary pathway (Young et al., 2015) may play a relevant role in the correlation between gastrointestinal microbiota and mastitis. Certain bacteria in the gastrointestinal tract can migrate to the mammary gland through the mechanism involving immune cells, that is, bacterial translocation (Berg, 1995; Addis et al., 2016). Therefore, improvement in profile of the gastrointestinal microbes is expected to ameliorate mastitis.

Inulin is a well-established dietary prebiotic with stimulating effect on probiotics, such as *Bifidobacterium* and *Lactobacillus*, etc., and suppressing effect on *Streptococcus*, *Clostridium*, and *Enterococcus*, etc., in human and monogastric animals (Ramirez-Farias et al., 2009). In ruminants, inulin was reported to decrease the production of ammonia, nitrogen, and methane, and increase the synthesis of microbial protein in the rumen (Samanta et al., 2013; Zhao et al., 2014). Tian et al. (2019) reported increased *Bacteroidetes* phyla and decreased *Firmicutes* phyla in the rumen of finishing beef steers fed inulin. Our previous study found that inulin supplementation increased propionate- and butyrate-producing bacteria (e.g., *Prevotella* and *Butyrivibrio*) and several beneficial commensal bacteria (e.g., *Muribaculaceae* and *Bifidobacterium*), and suppressed several proinflammatory bacteria (e.g., *Clostridia_UCG-014*, *Streptococcus*, and *Escherichia–Shigella*) in the rumen. Meanwhile, the concentration of serum inflammatory cytokines, such as IL-6 and TNF-α, was also reduced (Wang et al., 2021a). These findings suggested an optimizing effect of inulin on the rumen microbiota, which may further affect the inflammatory symptoms in mammary gland.

Given the bacterial transplantation and crosstalk (Berg, 1995; Wilson and Ebrary, 2008), the current study investigated whether dietary inulin compensation further affected the profiling of hindgut (feces) microbiota and metabolites. Furthermore, for a large part, the association of gut microbiota with major diseases can be attributed to metabolic signals of the microbiota that enter the circulation (Berggren et al., 1996; Vojinovic et al., 2019). Thus, in the current study, changes in serum metabolites and proteome profiles were simultaneously observed. Protein expression reflects the physiological and pathological conditions of the host. Blood is the most comprehensive and largest version of the host proteome (Kelly-Spratt et al., 2011). Therefore, the current study attempted to further explore the mechanism by which inulin affected inflammatory response of SCM from the perspectives of fecal microbiota and metabolites, as well as serum metabolites and protein profiles.

## Results

### Serum Lipid Levels

**FIGURE 1:**
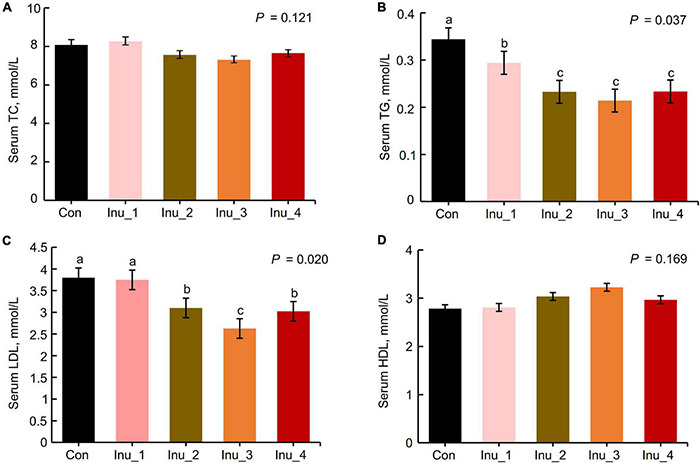
Comparison of serum lipids and proinflammatory cytokines among control and inulin treatment groups (*n* = 8). TC, total cholesterol; TG, triglyceride; LDL, low-density lipoprotein; HDL, high-density lipoprotein. The data are means and standard error (SEM). Means with different letters (a, b, c) are significantly different (*p* < 0.05). The *p*-value indicates the significance of the difference between the five groups. Con, control group; Inu_1, inulin_1 group, the inulin addition level was 100 g/day per cow; Inu_2, inulin_2 group, the inulin addition level was 200 g/day per cow; Inu_3, inulin_3 group, the inulin addition level was 300 g/day per cow; Inu_4, inulin_4 group, the inulin addition level was 400 g/day per cow.

Compared with the control group, serum triglycerides (TG) (*p* = 0.037) and low-density lipoprotein (LDL) (*p* = 0.020) decreased in the Inu_3 group (300 g/day per cow) (the concentration of TG had no significant difference among Inu_2 (200 g/day per cow), Inu_3, and Inu_4 (400 g/day per cow) groups). However, the concentration of serum total cholesterol (TC) (*p* = 0.121) and high-density lipoprotein (HDL) (*p* = 0.169) was not significantly changed among the five groups (Figure 1).

### Fecal Volatile Fatty Acids and Lactic Acid Concentrations

As shown in Table 1, compared with the control group, the concentrations of propionate (*p* = 0.041) and butyrate (*p* = 0.047) increased in Inu_3 and Inu_4 groups, and TVFA (*p* = 0.058) concentrations in feces tended to increase. Fecal pH (*p* = 0.086) and the ratio of acetate to propionate (A/P) (*p* = 0.061) tended to decrease after inulin supplementation. However, inulin supplementation had no significant effects on LA and other VFAs in feces of SCM cows.

**TABLE 1:** Effects of inulin addition on fecal lactate and short-chain fatty acids of subclinical mastitis (SCM) dairy cows.

	Groups (*n* = 8)						
Items	Con	Inu_1	Inu_2	Inu_3	Inu_4	SEM	*p*-value
pH	7.03	6.80	6.91	6.73	6.75	0.025	0.086
LA, mmol/L	0.99	1.02	1.13	1.02	1.15	0.015	0.141
Acetate, mmol/L	58.7	58.0	60.7	62.4	60.6	0.350	0.134
Propionate, mmol/L	10.3^c^	10.6^c^	13.1^b^	14.8^a^	14.4^a^	0.420	0.041
A/P	5.70	5.47	4.63	4.22	4.21	0.140	0.061
Butyrate, mmol/L	6.02^b^	6.08^b^	6.87^ab^	7.92^a^	7.64^a^	0.174	0.047
Isobutyrate, mmol/L	0.7	0.75	0.74	0.82	0.76	0.009	0.855
Valerate, mmol/L	0.88	1.02	0.77	0.95	1.09	0.025	0.264
Isovalerate, mmol/L	0.51	0.59	0.58	0.57	0.6	0.007	0.736
TVFA, mmol/L	75.3	76.3	83.5	85.2	85.4	0.989	0.058

*LA, lactic acid; A/P, the ratio of acetate to propionate; TVFA, total volatile fatty acids; Con, control group; Inu_1, inulin_1 group, the inulin addition level was 100 g/day per cow; Inu_2, inulin_2 group, the inulin addition level was 200 g/day per cow; Inu_3, inulin_3 group, the inulin addition level was 300 g/day per cow; Inu_4, inulin_4 group, the inulin addition level was 400 g/day per cow. ^a,b,c^Within a row, different letters differed significantly (p < 0.05).*

### Fecal Microbiota Diversity, Richness, and Composition

**FIGURE 2:**
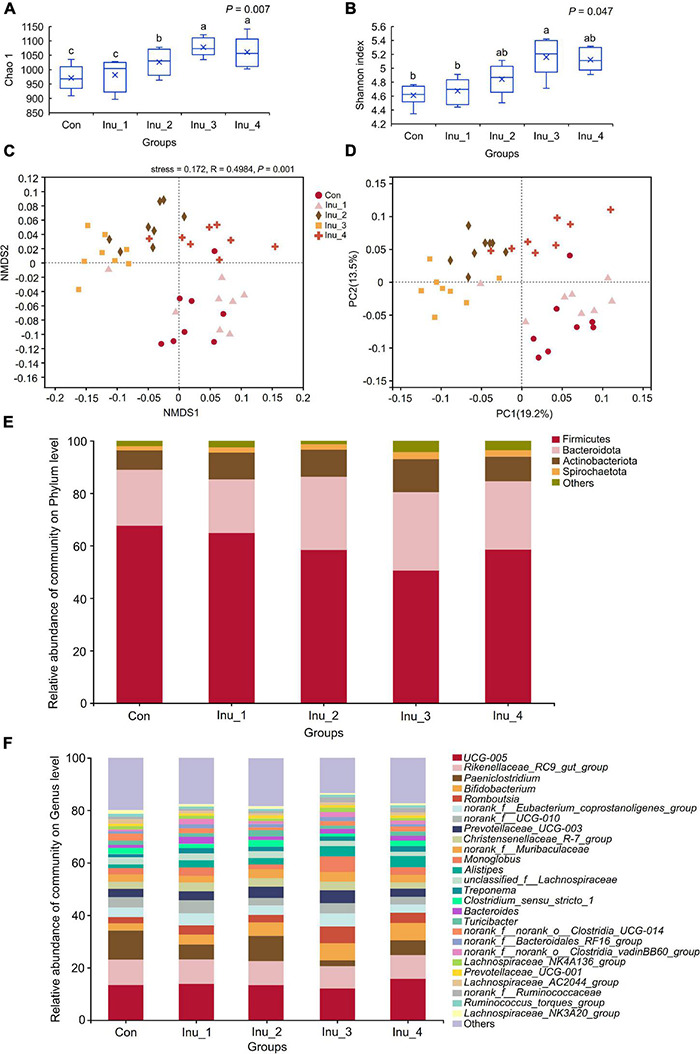
Changes in α- and β-diversity as well as composition of fecal microbiota in subclinical mastitic cows after inulin treatment. **(A)** Shannon and **(B)** Chao 1 index were the α-diversity indices, which reflected the diversity and richness of fecal microbiota, respectively. ^a,b^Different letters differed significantly (*p* < 0.05). **(C)** Non-metric multidimensional scaling (NMDS) and **(D)** principal coordinate analysis (PCoA) based on Bray–Curtis represented β-diversity, which displayed the profile of fecal microbial communities among groups. **(E,F)** Fecal microbiota composition at phylum and genus levels, respectively. Con, control group; Inu_1, inulin_1 group, the inulin addition level was 100 g/day per cow; Inu_2, inulin_2 group, the inulin addition level was 200 g/day per cow; Inu_3, inulin_3 group, the inulin addition level was 300 g/day per cow; Inu_4, inulin_4 group, the inulin addition level was 400 g/day per cow.

After merging and quality control of raw sequencing data, 1,245,375 high-quality 16S rRNA gene sequences were obtained from 40 fecal samples, with an average of 31,134 high-quality sequences per sample. To compare samples at the equal OTU sequence number level, the sequences of all samples were subsampled according to the minimum number of sequences (23,887 reads per sample). After cluster analysis, a total of 1,418 OTUs with > 97% similar level were obtained. As the number of reads increased, the rarefaction curve leveled off gradually, which reflected sufficient sequencing depth (Supplementary Figure 1). Meanwhile, the Good’s coverage in all groups was 99%, indicating adequate community coverage (Supplementary Table 2). Community alpha-diversity in feces is shown in Figures 2A,B and Supplementary Table 2. Compared with the control group, Shannon (*p* = 0.007), Simpson (*p* = 0.016), and Chao1 (*p* = 0.047) indices increased in the Inu_3 group (Shannon index in the Inu_3 and Inu_4 groups had no significant difference), implying elevated diversity and richness of fecal microbiota. Taxonomic analysis revealed 14 bacterial phyla (Supplementary Table 3), mainly including Firmicutes (60.3% ± 1.07%), Bacteroidota (30.0% ± 0.89%), Actinobacteriota (6.69% ± 0.14%), and Spirochaetota (2.11% ± 0.13%) (Figure 2E and Supplementary Figure 2A). At genus level, a total of 239 genera were identified. The top 100 bacteria in relative abundance are listed in Supplementary Table 4. Among the predominant genera, *UCG-005* (13.5 ± 0.16%), *Rikenellaceae_RC9_gut_group* (9.14 ± 0.08%), *Paeniclostridium* (6.16 ± 0.58%), *norank_f__Eubacterium_coprostanoligenes_group* (5.09% ± 0.16%), and *Bifidobacterium* (5.07% ± 0.23%) were the five most abundant ones (Figure 2F and Supplementary Figure 2B).

Non-metric multidimensional scaling and PCoA based on Bray–Curtis distance algorithm were used to compare the profile of microbial communities among groups (Figures 2C,D). The profile of fecal microbes in inulin treatment groups (except for Inu_1 group) could be basically separated from that in the control group. Furthermore, the HCA of the top 50 bacteria in relative abundance (Supplementary Figure 2C) revealed that the fecal microbiota was broadly divided into three categories in which *Paeniclostridium*, *Clostridia_UCG-014*, *Coprococcus*, *Ruminococcaceae*, *Peptostreptococcaceae*, etc., were concentrated in control and Inu_1 groups, while, *Bifidobacterium*, *Romboutsia*, *Christensenellaceae_R-7_group*, *Muribaculaceae*, *Alistipes*, etc., were mainly enriched in Inu_3 group.

### Difference of Fecal Microbiota Among Control and Inulin Treatment Groups

**FIGURE 3:**
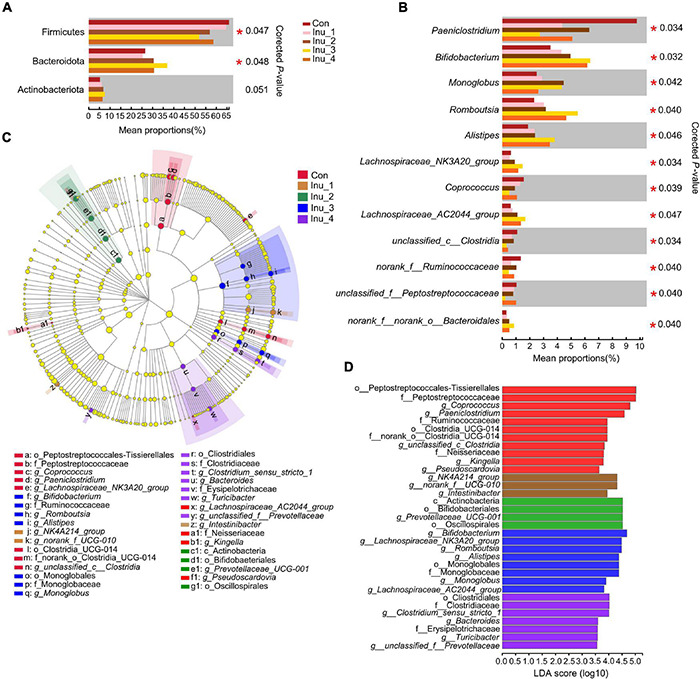
Significant differential microbiota in the feces of subclinical mastitic cows among control and different inulin treatment groups. **(A)** at phylum level; **(B)** at genus level. **(C,D)** Linear discriminant effect size (LEfSe) analysis and bar chart of linear discriminant analysis (LDA) score of different bacterial taxa in the feces, respectively. LDA score > 3.5. Con, control group; Inu_1, inulin_1 group, the inulin addition level was 100 g/day per cow; Inu_2, inulin_2 group, the inulin addition level was 200 g/day per cow; Inu_3, inulin_3 group, the inulin addition level was 300 g/day per cow; Inu_4, inulin_4 group, the inulin addition level was 400 g/day per cow. *0.01 < corrected *p*-value < 0.05.

At the phylum level, Bacteroidota (corrected *p* = 0.048) was increased in the Inu_3 group compared with the control group, whereas Firmicutes (corrected *p* = 0.047) was decreased in the Inu_3 group compared with the control group (the Firmicutes had no significant difference among Inu_2, Inu_3, and Inu_4 groups) (Figure 3A and Supplementary Table 3). At the genus level, compared with the control group, *Romboutsia* (corrected *p* = 0.040; LDA score = 4.16), *Monoglobus* (corrected *p* = 0.042; LDA score = 3.70), *Alistipes* (corrected *p* = 0.046; LDA score = 4.10), and *unclassified_o__Bacteroidales* (corrected *p* = 0.040; LDA score = 3.69), *Bifidobacterium* (corrected *p* = 0.032; LDA score = 4.64), *Lachnospiraceae_AC2044_group* (corrected *p* = 0.040; LDA score = 3.77), and *Lachnospiraceae_NK3A20_group* (corrected *p* = 0.034; LDA score = 4.16) were increased in Inu_3 group (*Alistipes*, *Lachnospiraceae_AC2044_group*, and *Bifidobacterium* between Inu_3 and Inu_4 had no significant difference; *Monoglobus* in Inu_2 and Inu_3 had no significant difference). However, *Paeniclostridium* (corrected *p* = 0.034; LDA score = 4.38), *norank_f__Ruminococcaceae* (corrected *p* = 0.047; LDA score = 3.85), *Coprococcus* (corrected *p* = 0.039; LDA score = 4.54) *unclassified_c__Clostridia* (corrected *p* = 0.034; LDA score = 3.78), and *unclassified_f__Peptostreptococcaceae* (corrected *p* = 0.040; LDA score = 4.78) were decreased in the Inu_3 group (*unclassified_c__Clostridia* between Inu_3 and Inu_4 had no significant difference) compared with control group (Figures 3B–D and Supplementary Table 4).

### Profile and Difference of Fecal and Serum Metabolites

**FIGURE 4:**
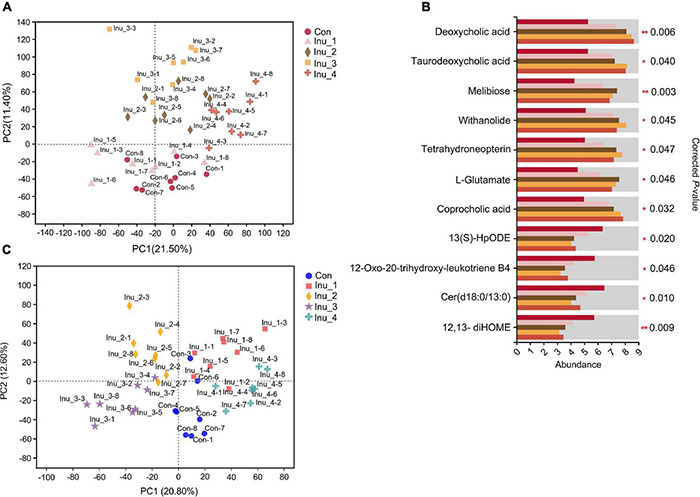
Changes in metabolites in feces and serum of subclinical mastitic cows after inulin treatment. **(A,C)** Principal component analysis (PCA) of the feces and serum metabolome among control and different inulin treatment groups. **(B)** Significant differential metabolites in the fecal sample among control and different inulin treatment groups. Con, control group; Inu_1, inulin_1 group, the inulin addition level was 100 g/day per cow; Inu_2, inulin_2 group, the inulin addition level was 200 g/day per cow; Inu_3, inulin_3 group, the inulin addition level was 300 g/day per cow; Inu_4, inulin_4 group, the inulin addition level was 400 g/day per cow. *0.01 < corrected *p*-value < 0.05; **corrected *p*-value < 0.01.

Total ion chromatogram (TIC) of QC samples in the feces and serum (Supplementary Figure 3) provided the overview of metabolome data. High similarity of retention time and total ion intensity among QC samples reflected the high accuracy of the metabolite data. Principal component analysis (PCA) was performed to assess the difference in samples among the five groups. In the PCA score plots, the inulin treatment groups could be separated from the control group, indicating the changes in inulin intake on the metabolic profile in the feces and serum of SCM dairy cows (Figures 4A,C). In the OPLS-DA score plots, R^2^X(cum) and R^2^Y(cum) represent the cumulative interpretation rate of the X and Y matrices of the model, respectively; Q^2^(cum) represents the predictive ability of the model. The closer these three indicators are to 1, the more stable and reliable the model is. In the current study, the R^2^X(cum), R^2^Y(cum), and Q^2^(cum) in all the models were above 0.50, indicating a high accuracy and prediction ability of the OPLS-DA model (Supplementary Figures 4, 5).

In total, 1,298 (715 and 583 in positive and negative ion modes, respectively) and 782 (423 and 359 in positive and negative ion modes, respectively) metabolites were identified in the feces and serum samples, respectively. The compound classification of fecal and serum metabolites at the superclass and subclass levels is shown in Supplementary Figure 6. Significant differential metabolites in the feces (Supplementary Tables 5−8) and serum (Supplementary Tables 9−12) samples, with VIP > 1, corrected *p* < 0.05 and FC > 1.5 or < 0.67, were detected between the control and Inu_1 groups, control and Inu_2 groups, control and Inu_3 groups, as well as control and Inu_4 groups, respectively. Furthermore, multigroup analysis of variance performed by Kruskal–Wallis H test was used to obtain the significant different metabolites among the five groups.

In the fecal samples (Figure 4B and Supplementary Table 13), compared with the control group, the levels of bile acid derivatives, including deoxycholic acid (corrected *p* = 0.006), taurodeoxycholic acid (corrected *p* = 0.040), and coprocholic acid (corrected *p* = 0.032) in the Inu_3 and Inu_4 groups were increased. Other elevated metabolites, including melibiose (corrected *p* = 0.003), withanolide (corrected *p* = 0.045), tetrahydroneopterin (corrected *p* = 0.047), L-Glutamate (corrected *p* = 0.046), and their highest values had no significant difference between the Inu_2 and Inu_3 groups. On the other hand, linoleic acid and arachidonic acid metabolites, including 12,13-diHOME (corrected *p* = 0.009; the lowest value was in Inu_3 group), 12-oxo-20-trihydroxy-leukotriene B4 (corrected *p* = 0.046; the lowest value was in Inu_3 group), and 13(S)-HpODE (corrected *p* = 0.020; the lowest value in Inu_2, Inu_3, and Inu_4 groups had no significant difference), as well as sphingolipid metabolite, Cer(d18:0/13:0) (corrected *p* = 0.010; the lowest value in Inu_2 and Inu_3 groups had no significant difference), were decreased compared with the control group.

**FIGURE 5:**
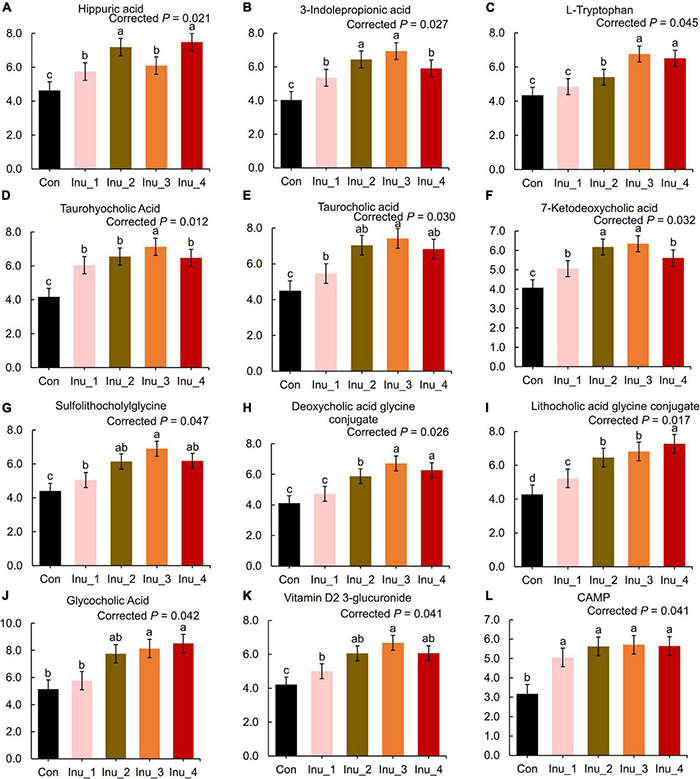
Secondary bile acid and amino acid metabolites in the serum of subclinical mastitic dairy cows. Con, control group; Inu_1, inulin_1 group, the inulin addition level was 100 g/day per cow; Inu_2, inulin_2 group, the inulin addition level was 200 g/day per cow; Inu_3, inulin_3 group, the inulin addition level was 300 g/day per cow; Inu_4, inulin_4 group, the inulin addition level was 400 g/day per cow. ^a,b,c^Different letters differed significantly (corrected *p* < 0.05).

**FIGURE 6:**
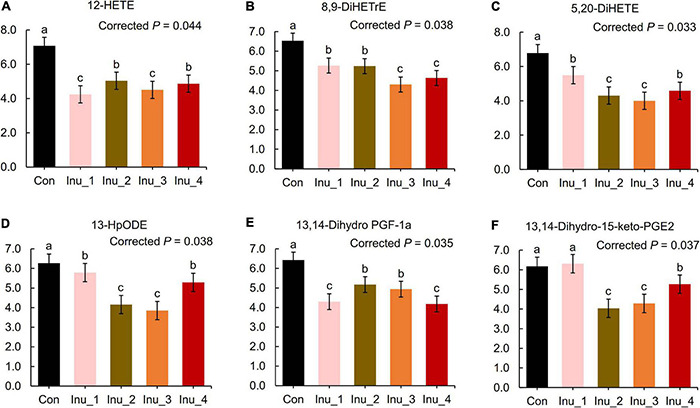
Lipid proinflammatory metabolites in the serum of subclinical mastitic dairy cows. Con, control group; Inu_1, inulin_1 group, the inulin addition level was 100 g/day per cow; Inu_2, inulin_2 group, the inulin addition level was 200 g/day per cow; Inu_3, inulin_3 group, the inulin addition level was 300 g/day per cow; Inu_4, inulin_4 group, the inulin addition level was 400 g/day per cow. ^a,b,c^Different letters differed significantly (corrected *p* < 0.05).

In the serum samples, bile acid derivates increased in the inulin treatment groups. When compared with the control group, taurohyocholic acid (corrected *p* = 0.012), sulfolithocholylglycine (corrected *p* = 0.047), and taurocholic acid (corrected *p* = 0.030) were elevated in the Inu_3 group. The highest values of glycocholic acid (corrected *p* = 0.042) and deoxycholic acid glycine conjugate (corrected *p* = 0.026) had no significant difference between the Inu_3 and Inu_4 groups. The highest values of 7-ketodeoxycholic acid (corrected *p* = 0.032) and lithocholic acid glycine conjugate (corrected *p* = 0.017) appeared in the Inu_3 and Inu_4 groups, respectively. Moreover, phenylalanine metabolite, hippuric acid (corrected *p* = 0.021; the highest value in Inu_2 and Inu_4 had no significant difference), and tryptophan metabolites, including L-tryptophan (corrected *p* = 0.045; the highest value in the Inu_3 and Inu_4 groups had no significant difference) and 3-indolepropionic acid (corrected *p* = 0.027; the highest value in the Inu_2 and Inu_3 groups had no significant difference) were increased with inulin intake (Figure 5 and Supplementary Table 13). In contrast, the arachidonic acid metabolites, 12-HETE (corrected *p* = 0.044; the lowest value was in the Inu_3 group), 8,9-DiHETrE (corrected *p* = 0.038; the lowest value in the Inu_3 and Inu_4 group had no significant difference), 13,14-dihydro-15-keto-PGE2 (corrected *p* = 0.037; the lowest value in the Inu_2 and Inu_3 groups had no significant difference), 13,14-dihydro PGF-1α (corrected *p* = 0.035; the lowest value in the Inu_1 and Inu_4 groups had no significant difference), as well as lineolic acid metabolites, 13-HpODE (corrected *p* = 0.038; the lowest value in the Inu_2 and Inu_3 group had no significant difference), and 5,20-DiHETE (corrected *p* = 0.033; the lowest value in the Inu_2 and Inu_3 groups had no significant difference) were decreased with inulin supplementation (Figure 6 and Supplementary Table 13). In both feces and serum samples, metabolic pathways related to bile acid metabolism, including bile secretion, bile acid, and secondary bile acid (SBA) biosynthesis were upregulated, while arachidonic acid and linoleic acid metabolism were downregulated (Table 2).

**TABLE 2:** Metabolic pathway enrichment analysis of significant differential metabolites in the feces and serum among control and inulin treatment groups.

KEGG pathway	Metabolites	*p*-value	Corrected *p*-value	Regulation
**Feces**				
Secondary bile acid biosynthesis	Deoxycholic acid	0.0017	0.019	**↑**
Bile secretion		0.0035	0.033	**↑**
Bile acid biosynthesis	Taurodeoxycholic acid	0.0057	0.043	**↑**
	Coprocholic acid			**↑**
Galactose metabolism	Melibiose	0.0057	0.043	**↑**
ABC transporters		0.0061	0.047	**↑**
D-Glutamine and D-glutamate metabolism	L-Glutamate	0.0022	0.022	**↑**
/	Withanolide	/	/	**↑**
/	Tetrahydroneopterin	/	/	**↑**
Linoleic acid and arachidonic acid metabolism	13(S)-HpODE	0.0044	0.034	↓
Arachidonic acid metabolism	12-oxo-20-dihydroxy-leukotriene B4	0.0047	0.039	↓
Sphingolipid metabolism	Cer(d18:0/13:0)	0.0036	0.010	↓
Linoleic acid metabolism	12, 13-DiHODE	0.0053	0.040	↓
**Serum**				
Bile secretion	CAMP	0.00002	0.0006	**↑**
	Glycocholic acid			
	Taurocholic acid			
Primary bile acid biosynthesis	Glycocholic acid	0.008	0.046	**↑**
	Taurocholic acid			
Secondary bile acid metabolism	Taurohyocholic acid	0.0072	0.0431	**↑**
	Deoxycholic acid glycine conjugate			
	Lithocholic acid glycine conjugate			
	7-Ketodeoxycholic acid			
	Sulfolithocholylglycine			
Phenylalanine metabolism	Hippuric acid	0.0004	0.0053	**↑**
	L-Tryptophan			
Tryptophan metabolism	3-Indolepropionic acid	0.0008	0.008	**↑**
	L-Tryptophan			
Serotonergic synapse	CAMP	0.0011	0.0093	**↑**
	L-Tryptophan			
/	Vitamin D2 3-glucuronide	/	/	**↑**
Arachidonic acid metabolism	12-HETE	0.004	0.0305	↓
	8,9-DiHETrE			
	13,14-Dihydro-15-keto-PGE2			
	13,14-Dihydro PGF-1α			
Lineolic acid metabolism	13-HpODE	0.0067	0.0425	↓
/	5,20-DiHETE	/	/	↓

*13(S)-HpODE. 13-L-hydroperoxylinoleic acid; 12, 13-DiHODE, 12, 13-hydroxyoctadecadienoate; CAMP, cyclic adenosine monophosphate; 12-HETE, 12(S)-hydroxyeicosatetraenoic acid; 8,9-DiHETrE, 8,9-dihydroxyeicosatrienoic acid; 5, 20-DiHETE, 5, 20-dihydroxyeicosatetraenoate; ABC transporters, ATP-binding cassette transporters;/, unannotated KEGG pathway; ↑=, the regulated metabolic pathway; ↓, the upregulated metabolic pathway.*

### Correlation Between Significantly Different Fecal Microbiota and Metabolites in Feces and Serum

**FIGURE 7:**
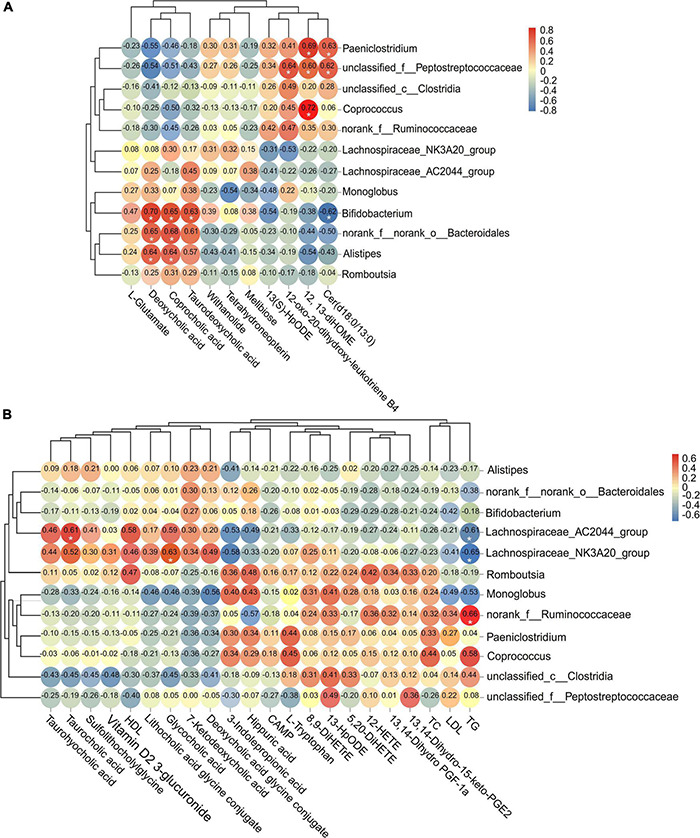
Analysis of the correlation between significant different fecal microflora and metabolites in **(A)** feces and **(B)** serum. The threshold value of correlation coefficient is −1.0 to 1.0. Red represents positive correlation, and blue represents negative correlation. *Corrected *p* < 0.05.

The abundance of *Bifidobacterium* was positively associated with the level of taurodeoxycholic acid (corrected *p* = 0.045), coprocholic acid (corrected *p* = 0.042), and deoxycholic acid (corrected *p* = 0.037), but negatively associated with cer(d18:0/13:0) (corrected *p* = 0.046). The abundance of *norank_f__norank_o__Bacteroidales* and *Alistipes* were positively associated with the level of deoxycholic acid (corrected *p* = 0.042; corrected *p* = 0.042) and coprocholic acid (corrected *p* = 0.040; corrected *p* = 0.042). The abundance of *Paeniclostridium* and *unclassified_f__Peptostreptococcaceae* were positively associated with the level of 12,13-diHOME (corrected *p* = 0.039; corrected *p* = 0.048) and cer(d18:0/13:0) (corrected *p* = 0.044; corrected *p* = 0.047). Meanwhile, *unclassified_f__Peptostreptococcaceae* was also positively associated with 12-oxo-20-dihydroxy-leukotriene B4 (corrected *p* = 0.044). The abundance of *Coprococcus* was positively associated with the level of 12,13-diHOME (corrected *p* = 0.037) (Figure 7A). In addition, the concentration of serum TG was positively associated with the abundance of *norank_f__Ruminococcaceae* (corrected *p* = 0.042), but negatively associated with *Lachnospiraceae_AC2044_group* (corrected *p* = 0.045) and *Lachnospiraceae_NK3A20_group* (corrected *p* = 0.042). The concentration of serum glycocholic acid and taurocholic acid were positively associated with the abundance of *Lachnospiraceae_NK3A20_group* (corrected *p* = 0.045) and *Lachnospiraceae_AC2044_group* (corrected *p* = 0.047), respectively (Figure 7B).

### Serum Protein Identification and Relative Quantification

**FIGURE 8:**
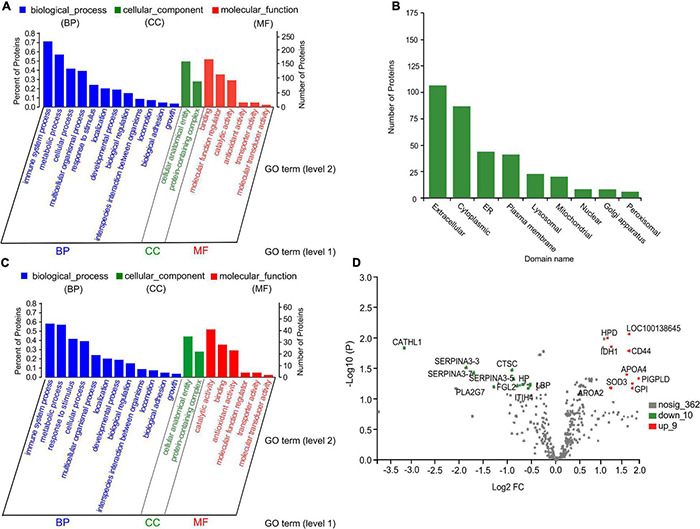
Gene ontology (GO) functional annotation, subcellular location class, and volcano plot of serum proteins. **(A,C)** GO bar chart of total proteins and differential proteins with *p* < 0.05, respectively. Each column represents a multilevel classification of GO. The abscissa represents the multilevel classification term of GO. The left and right ordinate represent the percentage of the proteins contained in the classification to total number of proteins, and the number of proteins in the classification, respectively. **(B)** Subcellular location annotation of total protein. ER, endoplasmic reticulum. **(D)** Volcano plot of significant differential proteins between the control and inulin_3 groups (300 g/day per cow). The abscissa is the fold change of protein expression between the two groups; the ordinate is corrected *p*-value of the difference in protein expression level. PLA2G7, platelet-activating factor acetylhydrolase; GPLD1, glycosyl-phosphatidylinositol-specific phospholipase D; HPD, 4-hydroxyphenylpyruvate dioxygenase; LOC100138645, amine oxidase; IDH1, isocitrate dehydrogenase (NADP); CD44, CD44 antigen; APOA4, apolipoprotein A-IV; FGL2, fibroleukin; SOD3, superoxide dismutase (Cu–Zn); GPI, glucose-6-phosphate isomerase; APOA2, apolipoprotein A-II; CATHL1, cathelicidin-1; SERPINA3-3, serpin A3-3; CTSC, Dipeptidyl peptidase 1; SERPINA3-7, serpin A3-7; SERPINA3-5, serpin A3-5; PIGPLD, phosphatidylinositol-glycan-specific phospholipase D; LBP, lipopolysaccharide-binding protein; HP, haptoglobin; ITIH4, inter-alpha-trypsin inhibitor heavy chain H4.

After the 10-plex TMT-nLC-MS/MS analysis, a total of 381 proteins from the five groups were identified. All proteins identified were compared with the GO database to obtain functional information. As shown in Figure 8A, most proteins were annotated to the functions related to immune system (70.0%), metabolic process (56.2%), binding (51.1%), and cellular anatomical entity (49.6%). In terms of subcellular localization, proteins belonging to extracellular, cytoplasmic, endoplasmic reticulum (ER), and plasma membrane accounted for more than 80% of the total proteins (Figure 8B).

### Functional Annotation of Differential Serum Proteins

Based on FC and *p*-value (FC > 1.2 or < 0.83; *p* < 0.05), a total of 79 differential proteins with 49 upregulated and 30 downregulated were identified among the control and four inulin treatment groups (Supplementary Tables 14, 15). Differential proteins were mainly involved in the immune system process (59.3%), metabolic process (59.0%), catalytic activity (51.2%), response to stimulus (40.0%), binding (36.5%), and antioxidant activity (29.1%) (Figure 8C). After correcting the *p*-values, 19 proteins with corrected *p* < 0.05 were obtained. As shown in Table 3, nine of the quantified proteins showed a more than twofold increase in serum sample of the Inu_3 group compared with the control group. In which, CD44 antigen (corrected *p* = 0.020), phosphatidylinositol-glycan-specific phospholipase D (corrected *p* = 0.042), and glucose-6-phosphate isomerase (corrected *p* = 0.048) were mainly related to immune system process. Amine oxidase (corrected *p* = 0.01), 4-hydroxyphenylpyruvate dioxygenase (corrected *p* = 0.011), isocitrate dehydrogenase (NADP; (corrected *p* = 0.017), and superoxide dismutase [Cu-Zn] (corrected *p* = 0.047) were mainly involved in oxidoreductase activity. Meanwhile, apolipoprotein A-IV (corrected *p* = 0.035), apolipoprotein A-II (corrected *p* = 0.049), and phosphatidylinositol-glycan-specific phospholipase D (corrected *p* = 0.042) were also associated with lipid transfer activity. On the other hand, of the 10 downregulated proteins, cathelicidin-1 (corrected *p* = 0.020) showed an almost ninefold decrease in the Inu_3 group compared with the control group. Cathelicidin-1 (corrected *p* = 0.020), haptoglobin (corrected *p* = 0.047), dipeptidyl peptidase 1 (corrected *p* = 0.037), platelet-activating factor acetylhydrolase (corrected *p* = 0.042), fibroleukin (corrected *p* = 0.043), and lipopolysaccharide-binding protein (corrected *p* = 0.045) were mainly involved in the immune system process. In addition, platelet-activating factor acetylhydrolase (corrected *p* = 0.042) was related to LDL particle remodeling. Serpin A3-3 (corrected *p* = 0.032), serpin A3-7 (corrected *p* = 0.040), serpin A3-5 (corrected *p* = 0.043), and inter-alpha-trypsin inhibitor heavy chain H4 (corrected *p* = 0.049) were related to enzyme inhibitor activity. The volcano plot of significant differential proteins between the control and Inu_3 groups is shown in Figure 8D.

**TABLE 3:** Significant differential proteins in the serum of SCM cows among control and different inulin treatment groups.

Accession ID	Protein name	Gene name	GO term level 2	GO term level 3	Inu_1/Con	Inu_2/Con	Inu_3/Con	Inu_4/Con	*p*-value	Corrected *p*-value
**Upregulated**										
A0A452DHX8	Amine oxidase	LOC100138645	Catalytic activity	Oxidoreductase activity, acting on the CH-NH_2_ group of donors	1.33	3.17	3.07	2.16	5.20E-05	0.010
A0A3Q1N2P4	4-Hydroxyphenylpyruvate dioxygenase	HPD	Catalytic activity	Dioxygenase activity	1.26	1.79	2.30	1.62	8.90E-05	0.011
A0A140T8A5	Isocitrate dehydrogenase [NADP]	IDH1	Binding; catalytic activity; metabolic process; response to stimulus	Nucleoside phosphate binding; oxidoreductase activity, acting on CH-OH group of donors; tricarboxylic acid cycle; response to oxidative stress	1.24	2.13	2.42	1.82	3.00E-04	0.017
F1MQT9	CD44 antigen	CD44	Molecular transducer activity; protein-containing complex; immune system process	Immune receptor activity; macrophage migration inhibitory factor receptor complex; lymphocyte activation	1.22	1.83	3.09	2.00	5.79E-04	0.020
F1N3Q7	Apolipoprotein A-IV	APOA4	Transporter activity; binding; protein-containing complex; multicellular organismal process; response to stimulus	Lipid transfer activity; sterol transporter activity; phospholipid binding; lipoprotein particle; innate immune response; plasma lipoprotein particle remodeling; innate immune response	1.30	2.74	3.02	2.18	0.001	0.035
P80109	Phosphatidylinositol-glycan-specific phospholipase D	PIGPLD	Catalytic activity; protein-containing complex; immune system process; metabolic process; localization	Hydrolase activity, acting on ester bonds; lipoprotein particle; immune response-activating signal transduction; lipid metabolic process; transport	1.26	3.08	3.24	2.41	0.003	0.042
F6R4N7	Superoxide dismutase [Cu-Zn]	SOD3	Catalytic activity; cellular process; response to stimulus	oxidoreductase activity, acting on superoxide radicals as acceptor; cellular oxidant detoxification; response to oxygen levels	1.23	2.88	3.43	2.40	0.002	0.047
Q3ZBD7	Glucose-6-phosphate isomerase	GPI	Binding; catalytic activity; immune system process; metabolic process	Signaling receptor binding; monosaccharide binding; intramolecular erythrocyte homeostasis; ATP generation from ADP	1.37	1.76	2.11	1.51	0.002	0.048
P81644	Apolipoprotein A-II	APOA2	Transporter activity; binding; protein-containing complex; multicellular organismal process; response to stimulus	Lipid transfer activity; sterol transporter activity; protein-lipid complex binding; lipoprotein particle; high-density lipoprotein particle clearance; defense response	1.21	2.35	2.62	1.97	0.002	0.049
A0A3Q1LRG2	Cathelicidin-1	CATHL1	Binding; immune system process; interspecies interaction between organisms; response to stimulus	Lipopolysaccharide binding; innate immune response; humoral immune response; response to bacterium; defense response	0.59	0.14	0.11	0.12	0.001	0.020
**Downregulated**										
G3N1U4	Serpin A3-3	SERPINA3-3	Molecular function regulator; biological regulation	Peptidase regulator activity; enzyme inhibitor activity; regulation of catalytic activity	0.61	0.39	0.30	0.70	0.001	0.032
Q3ZCJ8	Dipeptidyl peptidase 1	CTSC	Immune system process; biological regulation; cellular process; response to stimulus	Leukocyte-mediated immunity; regulation of immune system process; leukocyte mediated cytotoxicity; adaptive immune response	0.81	0.73	0.53	0.64	0.002	0.037
G8JKW7	Serpin A3-7	SERPINA3-7	Molecular function regulator; biological regulation	Peptidase regulator activity; enzyme inhibitor activity; regulation of catalytic activity	0.64	0.40	0.33	0.63	0.002	0.040
Q28017	Platelet-activating factor acetylhydrolase	PLA2G7	Binding; catalytic activity; protein-containing complex; biological regulation; metabolic process; multicellular organismal process	Phospholipid binding; hydrolase activity, acting on ester bonds; lipoprotein particle; regulation of immune system process; low-density lipoprotein particle remodeling	0.748	0.53	0.52	0.57	0.002	0.042
A2I7N1	Serpin A3-5	SERPINA3-5	Molecular function regulator; biological regulation	Peptidase regulator activity; enzyme inhibitor activity; regulation of catalytic activity	0.78	0.52	0.52	0.73	0.002	0.043
Q29RY7	Fibroleukin	FGL2	Molecular function regulator; immune system process; biological regulation	Peptidase regulator activity; leukocyte activation involved in immune response; immunoglobulin production; regulation of immune system process	0.73	0.75	0.59	0.64	0.002	0.043
Q2TBI0	Lipopolysaccharide-binding protein	LBP	Binding; immune system process	Lipopolysaccharide binding; lipoteichoic acid binding; cell activation involved in immune response; leukocyte activation involved in immune response; leukocyte chemotaxis; leukocyte migration involved in inflammatory response	0.73	0.81	0.71	0.70	0.003	0.045
Q2TBU0	Haptoglobin	HP	Binding; catalytic activity; interspecies interaction between organisms; response to stimulus	Hemoglobin binding; peptidase activity; response to bacterium; response to external biotic stimulus	0.73	0.60	0.58	0.64	0.003	0.047
A0A3Q1MA31	Inter-alpha-trypsin inhibitor heavy chain H4	ITIH4	Molecular function regulator; response to stimulus	Peptidase regulator activity; enzyme inhibitor activity; defense response	0.80	0.67	0.65	0.72	0.004	0.049

*GO, gene ontology; Con, control group; Inu_1, inulin_1 group, the inulin addition level was 100 g/day per cow; Inu_2, inulin_2 group, the inulin addition level was 200 g/day per cow; Inu_3, inulin_3 group, the inulin addition level was 300 g/day per cow; Inu_4, inulin_4 group, the inulin addition level was 400 g/day per cow.*

### Kyoto Encyclopedia of Genes and Genomes Pathway Enrichment Analysis of Differential Proteins

As shown in Table 4, upregulated proteins were mainly enriched in amino acid, lipid, and energy metabolic pathways, as well as peroxisome. Among them, 4-hydroxyphenylpyruvate dioxygenase and amine oxidase were enriched in phenylalanine (corrected *p* = 0.045) and tyrosine metabolism (corrected *p* = 0.049) pathway. Meanwhile, amine oxidase was also involved in tryptophan metabolism (corrected *p* = 0.065). Apolipoprotein A-IV and A-II were enriched in cholesterol metabolism pathway (corrected *p* = 0.186), and phosphatidylinositol-glycan-specific phospholipase D was involved in platelet activation (corrected *p* = 0.049). Glucose-6-phosphate isomerase was mainly involved in glycolysis/gluconeogenesis (corrected *p* = 0.067) and pentose phosphate pathway (corrected *p* = 0.068). In addition, superoxide dismutase (Cu–Zn) and isocitrate dehydrogenase (NADP) were enriched in peroxisome (corrected *p* = 0.041), and the latter also participated in glutathione metabolism (corrected *p* = 0.078) and citrate cycle (corrected *p* = 0.051).

**TABLE 4:** Kyoto encyclopedia of genes and genomes (KEGG) pathway enrichment analysis of significantly different proteins in serum among control and different inulin treatment groups.

KEGG pathway	Protein name	Gene name	*p*-value	Corrected *p*-value
Platelet activation	Phosphatidylinositol-glycan-specific phospholipase D	PIGPLD	0.022	0.049
Phenylalanine metabolism	4-Hydroxyphenylpyruvate dioxygenase	HPD	0.016	0.045
	Amine oxidase	LOC100138645		
Tyrosine metabolism	4-Hydroxyphenylpyruvate dioxygenase	HPD	0.021	0.049
	Amine oxidase	LOC100138645		
Tryptophan metabolism	Amine oxidase	LOC100138645	0.033	0.065
Peroxisome	Isocitrate dehydrogenase [NADP]	IDH1	0.019	0.041
	Superoxide dismutase [Cu-Zn]	SOD3		
Glutathione metabolism	Isocitrate dehydrogenase [NADP]	IDH1	0.037	0.078
Citrate cycle (TCA cycle)			0.021	0.051
ECM–receptor interaction	CD44 antigen	CD44	0.043	0.185
Cholesterol metabolism	Apolipoprotein A-IV	APOA4	0.052	0.186
	Apolipoprotein A-II	APOA2		
Glycolysis/gluconeogenesis	Glucose-6-phosphate isomerase	GPI	0.032	0.067
Pentose phosphate pathway			0.033	0.068
*Staphylococcus aureus* infection	Cathelicidin-1	CATHL1	0.009	0.037
	Haptoglobin	HP		
*Salmonella* infection	Lipopolysaccharide-binding protein	LBP	0.037	0.079
Complement and coagulation cascades	Serpin A3-3	SERPINA3-3	0.040	0.163
	Serpin A3-7	SERPINA3-7		
	Serpin A3-5	SERPINA3-5		
NOD-like receptor signaling pathway	Cathelicidin-1	CATHL1	0.023	0.057
	Dipeptidyl peptidase 1	CTSC		
Toll-like receptor signaling pathway	Dipeptidyl peptidase 1	CTSC	0.042	0.088
	Lipopolysaccharide-binding protein	LBP		
PI3K-Akt signaling pathway	Fibroleukin	FGL2	0.040	0.097
Ether lipid metabolism	Platelet-activating factor acetylhydrolase	PLA2G7	0.036	0.083
Rap1 signaling pathway	Fibroleukin	FGL2	0.042	0.157
MAPK signaling pathway	Inter-alpha-trypsin inhibitor heavy chain H4	ITIH4	0.035	0.069
	Fibroleukin	FGL2		
PPAR signaling pathway	Lipopolysaccharide-binding protein	LBP	0.033	0.065
NF-kappa B signaling pathway			0.017	0.044

*ECM, extracellular matrix; NOD, nucleotide oligomerization domain; PI3K, phosphatidylinositol 3′-kinase; Rap1, Ras-related protein 1; MAPK, mitogen-activated protein kinase; PPAR, peroxisome proliferators activate receptors.*

On the other hand, downregulated proteins were mainly concentrated in pathogen infection and inflammation-related signaling pathways. Cathelicidin-1 and haptoglobin might be involved in *Staphylococcus aureus* infection (corrected *p* = 0.037). Enrichment of lipopolysaccharide-binding protein was identified in the *Salmonella* infection pathway (corrected *p* = 0.079), NF-kappa B (corrected *p* = 0.044), peroxisome proliferator-activated receptors (PPAR), and Toll-like receptor (corrected *p* = 0.088) signaling pathways. Other downregulated proteins tended to be enriched in nucleotide oligomerization domain (NOD)-like receptor (corrected *p* = 0.057), phosphatidylinositol 3′-kinase (PI3K-Akt) (corrected *p* = 0.097), and MAPK (mitogen-activated protein kinase) (corrected *p* = 0.069) signaling pathways, respectively.

## Discussion

Based on the resent findings that mastitis was linked to the alterations in gastrointestinal microbiota and metabolites (Ma et al., 2018a; Zhong et al., 2018; Hu et al., 2020; Wang et al., 2021b), dietary intervention to remodeling the profile of microflora appeared to be a potential strategy in preventing mastitis. Inulin was extensively reported as the “food” of probiotics in the gastrointestinal tract (Shoaib et al., 2016; Guo et al., 2021). In the current study, dietary inulin supplementation affected the profile of fecal microbiota in the SCM cows. Although numerous studies have proven the beneficial regulation of dietary inulin on the gastrointestinal microflora, most were focused on monogastric animals (Ferrario et al., 2017; Li et al., 2019; Brosseau et al., 2021). However, in ruminants, inulin intake would first be used by ruminal microbes (Santoso et al., 2003; Zhao et al., 2014; Tian et al., 2019). In our previous *in vitro* study on the rumen degradation of inulin, nine levels of inulin including 0 (control group), 0.2, 0.4, 0.6, 0.8, 1, 1.2, 1.4, and 1.6% dry matter were added into fermentation bottles containing 0.5 g of basic diet, and then cultivated for 12, 24, and 36 h, respectively. The results showed that the degradation rate of inulin in the rumen leveled off when the inulin dose exceeds 1.2% (unpublished data). The inulin addition level in the current study was calculated based on the results of the *in vitro* experiment. It is, thus, speculated that the rumen undegraded inulin from high-dose groups (the Inu_3 and Inu_4 groups) might enter in the hindgut with chyme and be fermented by gut microbiota, which might also explain the result that fecal microbiota and metabolites showed significant changes in the Inu_3 and Inu_4 groups.

On the other hand, the changes in ruminal microbiota might also cause the shifts in fecal microbiota. In the current study, inulin consumption increased the Bacteroides phyla and decreased the Firmicutes phyla in the feces, which was consistent with the response of rumen microflora after inulin treatment in our previous study (Wang et al., 2021a). At the genus level, increased *Bifidobacterium*, *Lachnospiraceae_NK3A20_group*, as well as decreased *Coprococcus* and *Ruminococcaceae* abundances, were also identified in both feces and rumen (Wang et al., 2021a), suggesting several commonly modulated microbiomes between rumen and feces of dairy cows. Bacteria in the gastrointestinal tract can migrate to other niches *via* blood circulation (Berg, 1995; Samanta et al., 2013; Young et al., 2015). Lymphocytes in the gut-associated lymphoid tissue (GALT) enter into the mammary gland to form an entero-mammary gland connection, which is formed by the migration of immune cells (Addis et al., 2016). Especially, dendritic cells (DCs) can sample the intestinal contents, including live bacteria, by opening the tight junctions between intestinal cells without compromising the integrity of the epithelial barrier and carrying them to the mesenteric lymph nodes (Rescigno et al., 2001). Thus, these intestinal bacteria have the opportunity to spread to the mammary glands through the mucosal-associated lymphatic system (Young et al., 2015; Addis et al., 2016). Furthermore, microflora proliferation might promote bacterial translocation (Berg, 1995). It is, therefore, reasonable to speculate that the effect of inulin compensation on fecal microbiota of SCM cow might be related to the increased corresponding ruminal community abundance and diversity, which further promote bacterial translocation from the foregut (rumen) to the hindgut (rectum) *via* chyme flow.

Consistent with most studies, dietary inulin increased the relative abundance of Bacteroidetes and reduced Firmicutes in the feces (Ferrario et al., 2017; Li et al., 2019; Brosseau et al., 2021). The enriched abundance of the Bacteroidetes phylum was attributed to the fact that most bacteria in this phylum have genes encoding hydrolases to degrade non-starch carbohydrate (Dodd et al., 2011; He et al., 2021). It is reported that only a few selective bacteria in the gastrointestinal tract were able to ferment inulin, including *Bacteroides* and *Bifidobacteria* (Roberfroid, 1993), which was in agreement with our observation. The Bacteroidetes phylum and several Firmicutes phylum produced propionate primarily *via* the succinate pathway, and butyrate was also a main product of the Firmicutes phylum (Louis et al., 2014). Increased propionate and butyrate levels in the inulin groups were also observed in the rumen (Zhao et al., 2014; Tian et al., 2019; Wang et al., 2021a). Thus, in the present study, the increased *norank_f_norank_o__Bacteroidales* and *Alistipes* (Shkoporov et al., 2015) in Bacteroidetes phylum might be the main contributors to the increase in propionate. *Lachnospiraceae* (Lepage et al., 2011) and *Bifidobacterium* (Abbeele et al., 2011) were closely related to butyrate production. However, inulin supplementation suppressed several inflammation-related bacteria, which were mainly from the Firmicutes phylum. *Coprococcus* and *Ruminococcaceae* were detected to be significantly enriched in feces (Ma et al., 2018a) and rumen (Wang et al., 2021a) of mastitic cow. In addition, *Paeniclostridium* (formerly known as *Clostridium*) (Vidor et al., 2015) and *Peptostreptococcus* (Wensinck et al., 1983) infection were related to severe enteritis. These changes in microbiome indicated a potential correlation between disturbance in gut microflora and mastitis. The decrease in these inflammation-related bacteria in inulin groups in the current study might be related to the proliferation of probiotics, such as *Bifidobacteria* and the antibacterial property of propionate (Ciarlo et al., 2016).

Gut microbiota implicated in mastitis has also been linked to the level of lipids in the circulation (Vojinovic et al., 2019). Inflammation could trigger obvious variation in lipid metabolism (Khovidhunkit et al., 2004). Recent studies have shown the dyslipidemia during LPS-induced mastitis, including elevated plasma LDL, TG, and TC concentrations and lowered HDL level (Memon et al., 2000; Xiao et al., 2012, 2017). In the current study, inulin supplementation decreased serum TG and LDL concentrations. Vojinovic et al. (2019) confirmed the positive correlation of serum TG with genera from the family *Ruminococcaceae*, a gut microbe linked to low gut microbial richness (Fu et al., 2015), and negative association with the family *Lachnospiraceae*, which was consistent with our observation. In addition, higher microbiome diversity was significantly associated with lower levels of serum LDL particles and TG (Vojinovic et al., 2019). These findings indicated that the decreased serum TG and LDL in SCM cows fed inulin might be linked to increased fecal microbiota diversity and variation in the abundance of *Ruminococcaceae* and *Lachnospiraceae*.

Short-chain fatty acids in the gut can be absorbed into the portal circulation, acting as a source of SCFAs in the bloodstream (Berggren et al., 1996). Additionally, increased propionate and butyrate in the rumen after inulin compensation (Wang et al., 2021a) might also enter the blood through the rumen wall. It is reported that propionate could inhibit cholesterol synthesis and promote bile acid metabolism (Demigné et al., 1995; Beylot, 2005). Inulin supplementation promoting bile acid metabolites was observed in both feces and serum in the current study. These increased metabolites upregulated bile secretion and the SBA biosynthesis metabolism pathways. Roberfroid (1993) suggested that inulin in the hindgut was almost quantitatively fermented almost exclusively by *Bifidobacteria* and *Bacteroides*, which were the two main gut microbes involved in bile acid metabolism, and could convert primary bile acids into SBAs (Jia et al., 2018). Moreover, in the present study, the positive correlation of *Lachnospiraceae* with taurocholic acid and glycocholic acid was reported. Genera from *Lachnospiraceae* are also reported to be involved in the conversion of primary bile acids into SBAs (Vojinovic et al., 2019). Gut microbiota-mediated bile acid was observed to modulate the innate immune system of the host (Ma et al., 2018b). Bile acid receptors, farnesoid X receptors (FXR), and G protein-coupled receptor (TGR5) are capable of antagonizing the inflammatory response and inhibit proinflammatory cytokines by inhibiting the activation of NF-κB and inflammasomes, respectively (Wang et al., 2008; Yoneno et al., 2013). Secondary bile acids have a broad anti-inflammatory role. Among them, deoxycholic acid is an effective agonist of FXR and TGR5, which could reduce leukocyte infiltration and tissue inflammation (Yoneno et al., 2013). In addition, loss of SBAs in the serum might be linked to inflammasome activation (Hagan et al., 2019). The current study indicated that enrichment of *Bacteroides* and *Bifidobacteria* in the gastrointestinal tract promoted SBA production, which might exert anti-inflammatory effects.

Galactose metabolism in mastitic cows’ feces was observed to be depleted, which was recovered by probiotics intake (Ma et al., 2018a). In the current study, inulin ingestion enhanced the abundance of melibiose in the feces, which was involved in galactose metabolism. A similar observation was in the rumen of SCM cow fed inulin (Wang et al., 2021a). Amino acid metabolism was another upregulated metabolic pathway both in the feces and serum affected by inulin. In agreement with Drabińska et al. (2018), inulin supplementation increased fecal L-glutamate concentration in the current study, which was also previously observed in the rumen of SCM cow fed inulin (Wang et al., 2021a). Increased L-glutamate concentration suggested that inulin could improve intestinal integrity and influencing immune response (Nose et al., 2010; Ruth and Field, 2013). In addition, gut microbiota can convert L-tryptophan into derived indole (Krishnan et al., 2018). Indole propionic acid enhances the intestinal barrier function and suppresses TNF-α production (Marjo et al., 2018). The above observations indicated the potential role of inulin supplementation in maintaining the intestinal barrier and anti-inflammatory effects.

In contrast, arachidonic acid and linoleic acid metabolites in the inulin groups were downregulated compared with the control group in both feces and serum. 12-Oxo-20-dihydroxy-leukotriene B4, 12-HETE, and 8,9-DiHETrE are lipoxygenase metabolites of arachidonic acid. 13,14-Dihydro-15-keto-PGE2, 13,14-dihydro-PGF-1α are prostaglandin metabolites. The above metabolites are major proinflammatory mediators (Murphy and Gijón, 2007; Aoki and Narumiya, 2012). Downregulated 12-Oxo-20-dioxy-leukotriene B4 was also observed in the rumen of SCM cows fed inulin (Wang et al., 2021a). Decreased proinflammatory oxidized lipid products in the feces and serum suggested the positive regulation of inflammatory response. In addition, the changes in fecal microflora and metabolites compared with ruminal community and metabolites in SCM cows displayed partial similarity, further suggesting the crosstalk between the rumen and hindgut bacteria, or that there were commonly regulated microbiota and metabolites between the two niches.

Changes in serum protein further showed the effect of inulin supplementation on the inflammatory response of SCM cows. In the current study, upregulated proteins were mainly related to the immune system process, lipid transfer activity, and oxidoreductase activity. Among them, CD44 was reported to enhance the phagocytic ability of macrophages to apoptotic neutrophils during inflammation to prevent the release of proinflammatory mediators (Vivers et al., 2002). In the serum, most phosphatidylinositol-glycan-specific phospholipase D was related to HDL, and its activity depended on apolipoprotein A (Hoener and Brodbeck, 2010). In addition, isocitrate dehydrogenase (NADP) and superoxide dismutase (Cu–Zn) belonging to peroxisome were increased, indicating that the inulin supplementation might affect oxidative stress during SCM. 4-Hydroxyphenylpyruvate dioxygenase is the key enzyme of the phenylalanine, tyrosine, and tryptophan biosynthesis pathway in phenylalanine metabolism. Amine oxidase is the key enzyme for the conversion of tryptamine to indol-3-acetaldehyde in the tryptophan metabolism pathway. The above two enzymes further explained the changes in phenylalanine and tryptophan metabolism observed in serum metabolome.

Of the downregulated serum proteins, cathelicidin-1, haptoglobin, serpin A3, and inter-alpha-trypsin inhibitor heavy chain H4 were also found to be increased significantly in the whey of SCM cow (Zhang et al., 2015). Increased cathelicidin-1 might be linked to the antibacterial activity and chemotaxis of neutrophils in inflammatory responses (Zanetti, 2004). Haptoglobin and inter-alpha-trypsin inhibitor heavy chain H4 were acute phase proteins reported in mastitis (Pineiro et al., 2004; Quaye, 2008). Our previous study showed inhibited serum IL-6 concentration in SCM cows fed inulin (Wang et al., 2021a). IL-6 was reported as the main inducer of the haptoglobin expression (Quaye, 2008), suggesting that inulin-suppressed serum IL-6 might lead to the downregulation of haptoglobin. Moreover, lipopolysaccharide binding protein could control infection by binding to LPS and transferring it to the receptor on the surface of macrophages (Jack et al., 1997). As mentioned above, the serum LPS concentration in SCM cows was decreased after inulin supplementation, which might explain the decrease in lipopolysaccharide-binding protein. The current study showed that cathelicidin-1 and haptoglobin were related to *Staphylococcus aureus* infection. It is reasonable to speculate that the downregulation of the above two proteins was attributed to the decreased abundance of *Staphylococcus* in milk and rumen of SCM cows fed inulin (Wang et al., 2021a). Moreover, the downregulated platelet-activating factor acetylhydrolase might be associated with the decreased plasma LDL concentration (Guerra et al., 1997). Overall, the changes in these serum proteins reflected the effect of inulin supplementation on the inflammatory response, pathogen infection, and lipoprotein metabolism during SCM.

In general, in the light of the link between the gastrointestinal microbiota profiles and mastitis, we attempted to ameliorate inflammatory symptoms *via* dietary inulin compensation. The effect of inulin supplementation on fecal microbiome and metabolites appeared to be partly related to the alternation in ruminal microbiota and metabolites. Inulin intake changed the profiling of ruminal bacteria, and metabolites have been observed in our previous study. In addition, residual rumen-undegraded inulin in high-dose groups might also enter the hindgut with chyme and be utilized by gut microorganisms. Changes in gastrointestinal metabolites, including SCFAs, further affected serum metabolites. Although the specific mechanism by which inulin affects serum proteome is still unclear, altered proteins preliminarily displayed the effect of inulin supplementation on inflammatory reactions of SCM cows.

## Conclusion

Inulin consumption increased the abundance of *Bacteroides*, *Bifidobacteria*, and *Lachnospiraceae*, and reduced the abundance of *Paeniclostridium*, *Peptostreptococcaceae*, *Clostridia*, *Ruminococcaceae*, and *Coprococcus* in feces of SCM cows. Meanwhile, increased fecal microbiota diversity and changes in the abundance of *Lachnospiraceae* and *Ruminococcaceae* might be correlated with decreased serum TG and LDL. Elevated SBAs with anti-inflammation properties were observed in feces and serum samples from inulin-fed SCM cows, which might be mediated by increased *Bacteroides*, *Bifidobacteria*, and *Lachnospiraceae*. Conversely, proinflammatory oxidized lipid products were decreased in both feces and serum. Serum proteins related to immune response, lipid transport, and antioxidative stress were upregulated in SCM cows fed inulin, while acute phase proteins were downregulated. Collectively, the current study provided a potential mechanism of inulin in alleviating inflammation through the investigation of fecal microbiota, metabolites and serum metabolites, and proteins in SCM cows.

## Materials and Methods

### Ethics Statement

The experimental design conducted in the current study was approved by the Experimental Welfare Ethics Committee, Institute of Animal Science and Veterinary Medicine, Chinese Academy of Agricultural Sciences (Beijing, China; approval number: IAS-2020-92).

### Animals, Diets, and Experimental Design

Forty Holstein dairy cows with milk SCC of 718,000 ± 15,000 cells/ml, the result of California mastitis test (CMT) of weakly positive and positive, no clinical symptoms in udders, average dry matter intake of 25.0 ± 0.27 kg/day, average days in milk of 134 ± 7.0 days, average parity of 3.12 ± 0.610 and average milk yield of 29.6 ± 1.52 kg/day were randomly assigned to five treatments (n = 8/treatment), and housed in individual tie stalls. The basal diet for all cows was a total mixed ratio (TMR) with a concentration to forage ratio of 40:60 (Supplementary Table 1) and offered three times a day, at 07:30, 13:30, and 19:30, respectively. Inulin (purity > 90%) used in the current study was extracted from the tuber of Jerusalem artichoke and provided by Langfang Academy of Agriculture and Forestry Sciences (Hebei, China). To probe an appropriate range of inulin addition, an *in vitro* study by rumen simulation technique was performed before the current study. In the *in vitro* trial, nine levels of inulin including 0 (control group), 0.2, 0.4, 0.6, 0.8, 1, 1.2, 1.4, and 1.6% dry matter (DM) were added into the incubation bottle with 0.5 g of dried TMR (the TMR used in the *in vitro* test was the same as that used in the current study), respectively. Three incubation times, 12, 24, and 36 h, were set for each addition level (unpublished data). Based on the results of *in vitro* test and the average dry matter intake (25 kg/day per cow) of cows in the current study, inulin was added at five levels in the present study, including 0 (control), 100 (Inu_1 group), 200 (Inu_2 group), 300 (Inu_3 group), and 400 (Inu_4 group) g/day per cow, respectively, which were provided three times at the time of feeding TMR. To ensure the accuracy of inulin intake, it was accurately weighed and made into pills with the same size in Langfang Academy of Agriculture and Forestry Sciences (Hebei, China), which was offered by a 1-m-long feeder made of stainless steel (Jinhaifeng Animal Husbandry Technology Co., Ltd., Sichuan, China) through the oral cavity. The animal trial was conducted for eight weeks.

### Serum and Feces Sample Collection

Sampling was conducted on day 56. Two tubes of 5-ml blood samples from each cow were collected into the procoagulant inert separation tube through the tail vein at 1 h before the morning feeding. All blood samples were held at ambient temperature for 40 min and then centrifuged at 3,000 × *g* and 4°C for 15 min to obtain serum, which was stored in three 1.5-ml sterile tubes. One of the serum samples was stored at −20° for analysis of serum lipids, including TC, TG, HDL, and LDL. The residual two serum samples were stored at −80°C for analysis of serum metabolome and proteome. Sterile long-arm gloves were used to collect feces samples from the rectum at 3 h after morning feeding, which were then collected into sterile sealed plastic bags. Fecal pH was measured by inserting a portable pH meter (Mettler Toledo, Zurich, Switzerland) directly into the feces samples. Feces sample from each cow was stored in three 1.5-ml sterile tubes. One of them, for quantification of lactic acid (LA) and volatile fatty acids (VFAs), was stored at −20°. The other two were stored at −80° for analysis of fecal microbiota and metabolites.

### Serum Lipid Assay

Serum TC (MAK266), TG (MAK043), LDL, and HDL (MAK045) were determined by colorimetric kits (Sigma-Aldrich, Darmstadt, Germany) and cuvettes. The detection wavelength of TC and TG was 500 nm, and that of HDL and LDL was 546 nm.

### Feces Volatile Fatty Acids and Lactic Acid Assay

For quantification of VFAs, 0.5 g of thawed feces sample was combined with 8 ml of acid diluent (15 ml, 100 mmol/L of 2-ethylbutyric acid, and 50 ml, 5 mmol/L of hydrochloric acid) in a 20-ml centrifuge tube. After mixing by vortexing for 2 min, the solution was centrifuged at 10,000 × *g* and 4° for 20 min. The supernatant was transferred to a 10-ml centrifuge tube. A 1-ml supernatant was filtered by a 0.45-μm fiber filter into a sample bottle and then detected by Agilent 5975C gas chromatograph (Agilent, CA, United States) fitted with a flame-ionization detector. The samples (2 μl) were injected through the split injection port (50:1) onto a chromatographic column (PTX-Wax) (30.0 m × 0.25 mm column × 0.20 μm). The oven temperature was initially set at 60°C for 3 min and then increased at 10°C/min to 140°C and then held for 30 min. The detector temperatures were maintained at 300°C (Sato, 2009). The concentration of LA was determined by using a lactate assay kit (MAK064, Sigma-Aldrich, Darmstadt, Germany). A Multiskan Ascent microplate reader (Thermo Fisher Scientific, MA, United States) was used to detect the OD value at 570 nm.

### Fecal Bacterial DNA Extraction, 16S rRNA Gene PCR Amplification, and Sequencing

TIANamp Stool DNA kit (Tiangen Biotech Co., Ltd., Beijing, China) was used to extract fecal microbial genomic DNA. A 200-mg feces sample from each cow was weighed and added to a 2-ml centrifuge tube with 500 μl of buffer SA, 100 μl of buffer SC, 15 μl of proteinase K, and 0.25 g of grinding beads, which was mixed thoroughly by using a TGrinder H24 tissue grinding homogenizer (OES-TH-01, Tiangen Biotech Co., Ltd., Beijing, China) and incubated for 15 min. After centrifugation at 13,400 × *g* for 3 min, the supernatant was transferred to a centrifuge tube with 10 μl of RNase A, which was then mixed thoroughly and held for 5 min. After centrifugation at 13,400 × *g* for 3 min, the supernatant was transferred to a centrifuge tube, and an equivalent volume of buffer GFA was added. The solution was added to an adsorption column CR2. After centrifugation at 13,400 × *g* for 3 s, the waste liquid was discarded. A 700-μl buffer PW was added to the adsorption column CR2, which was centrifuged at 13,400 × *g* for 30 s. After discarding the waste liquid poured out, the column CR2 was dried at ambient temperature. A 50-μl eluting buffer TB was added to the adsorbent membrane and held for 5 min at room temperature. After centrifugation at 13,400 × *g* for 2 min, the total DNA was obtained. DNA fragment concentrations and purity were detected by 2% agarose gel electrophoresis (Liuyi Biological Technology Co., Ltd., Beijing, China) and ultraviolet spectrophotometer (Mettler Toledo, Zurich, Switzerland).

The hypervariable region V3–V4 of the bacterial 16S rRNA gene was amplified with primer pairs 338F (5′-ACTCCTACGGGAGGCAGCAG-3′) and 806R (5′-GGACTACHVGGGTWTCTAAT-3′) by ProFlex PCR thermocycler (Applied Biosystems, CA, United States) (Wu et al., 2016). The PCR amplification was performed as follows: initial denaturation at 95°C for 3 min, followed by 27 cycles of denaturing at 95° for 30 s, annealing at 55° for 30 s, extension at 72° for 45 s, and single extension at 72° for 10 min until halted. The PCR mixtures contained 5 × FastPfu buffer 4 μl, 2.5 mM dNTPs 2 μl, forward primer (5 μM) 0.8 μl, reverse primer (5 μM) 0.8 μl, FastPfu DNA polymerase 0.4 μl, template DNA 10 ng, and finally ddH_2_O up to 20 μl. The PCR product was identified, purified, and quantified by using 2% agarose gel electrophoresis (Liuyi Biological Technology Co., Ltd., Beijing, China), PureLink™ PCR purification kits (Thermo Fisher Scientific, MA, United States) and Quantus™ Fluorometer (Promega, United States), respectively. The purified amplified fragment was performed library constructed on the Illumina MiSeq platform (Illumina, San Diego, CA, United States): (i) connection of the “Y”-shaped adapter, (ii) self-linked fragments of the adapter was removed by using magnetic beads to screen, and (iii) the library template was enriched by using PCR amplification; (iv) sodium hydroxide denaturation was done, and single-stranded DNA fragments were produced. Sequencing was performed on Illumina’s Miseq PE300 platform (Illumina, San Diego, CA, United States) (Wu et al., 2016).

### Sequencing Data Processing

The raw sequence was quality controlled by using Trimmomatic^1^ and merged paired-end reads by using the FLASH software (version 1.2.11).^2^ Operational taxonomic unit (OTU) clustering of the high-quality sequences with > 97% similarity was conducted by using the Uparse software (version 7.0.1090).^3^ The OTU table was rarefied at 23,887 reads per sample. The RDP Classifier (version 2.11)^4^ was used to analyze taxonomic information of OTU representative sequences with > 97% similarity, which was compared with the Silva database (Release138).^5^ Alpha diversity was calculated by using Mothur (version 1.30.2).^6^ Rarefaction curve was drawn based on alpha diversity index (Shannon and Chao 1) using package in R. Beta diversity was estimated using Qiime (version 1.9.1).^7^ Among them, principal coordinates analysis (PCoA) and non-metric multidimensional scaling analysis (NMDS) were based on Bray–Curtis distance algorithm. Bacteria hierarchical clustering analysis (HCA) was conducted by using vegan package in R (version3.3.1). Significant differential microbiota among five groups was analyzed by Kruskal–Wallis H test. False discovery rate (FDR) was used to correct the *p*-value, with statistical significances declared at the corrected *p* < 0.05. Tukey–Kramer was used for *post hoc* test. Linear discriminant analysis effect size (LEfSe)^8^ was used to further detect the difference in microbial relative abundance among different groups, and linear discriminant analysis (LDA) was used to estimate the impact of these differential microbiota on the difference among groups. Significant different microbiota were considered as LDA > 3.5 and corrected *p*-value < 0.05.

### Feces and Serum Metabolomics Analysis

A total of 50 mg of feces and 100 μl of thawed serum samples from each cow were used for untargeted metabolomic analysis on liquid chromatography-mass spectrometry (LC-MS) platform (UPLC-TripleTOF, AB SCIEX, MA, United States), respectively. Samples were added into a 1.5-ml sterile centrifuge tube with 400 μl of extract solution (acetonitrile:methanol = 1:1), and thoroughly vortex mixed. The solution was centrifuged at 13,000 × *g* and 4° for 5 min. The supernatant was ultrasonically extracted at 5°C and 40 kHz for 5 min, and then centrifuged at 13,000 × *g* at 4°C for 5 min. The supernatant was transferred to a sample vial for LC-MS analysis, with an injection volume of 10 μl. Quality control (QC) samples of feces and serum were from a mixture of equal volume of 40 feces and serum samples, respectively. Chromatographic separation was through HSS T3 chromatographic column (100 mm × 2.1 mm i.d., 1.8 μm, Waters, Milford, CT, United States) at flow rate and column temperature of 0.40 ml/min and 40°, respectively. Mobile phase A contained water and 0.1% formic acid, and mobile phase B contained 0.1% formic acid and equal volume of acetonitrile and isopropanol. Separation gradient: 0–3 min, 95%: 5%; 80%: 20% at 3 min; 5%: 95% for 9–13 min; 95%: 5% for 13.1–16 min. Mass spectrum signal was acquired *via* positive and negative ion scanning mode. Ion spray voltages at positive and negative ion modes were 5,000 and 4,000 V. Ion source heating temperature and cyclic collision energy were 500° and 20–60 V (Wang et al., 2021b).

### Metabolomics Data Processing

LC-MS raw data were performed at baseline filtering, peak identification, integration, retention time correction, and peak alignment by using the Progenesis QI software (Waters, Milford, CT, United States). Finally, a data matrix of retention time, mass-to-charge ratio (m/z), and peak intensity were obtained. After normalizing the response intensity of mass spectrum peaks, the human metabolome database (HMDB)^9^ was used to obtain metabolite information. PCA and orthogonal partial least-squares discrimination analysis (OPLS-DA) in ropls packages of R (version1.6.2) were used to reflect difference among groups. Response permutation testing (RPT) was used to evaluate the accuracy of OPLS-DA models. Significant differentially expressed metabolites were calculated by Kruskal–Wallis H test. The *p*-value was corrected by FDR. Variable importance in the projection (VIP) from OPLS-DA models (SciPy in Python, version 1.6.2) was used to assess the contribution of the metabolites to the difference between the two groups. Significant differential metabolite was declared as corrected-*p*-value < 0.05, VIP > 1, and fold change (FC) > 1.5 or < 0.67. KEGG metabolic pathway enrichment was analyzed using SciPy in Python (version 1.0.0). Spearman’s correlation analysis was used to determine the correlation between fecal microbiota and metabolites in feces and serum. The correlation coefficients range from −1.0 to + 1.0. The *p*-value was corrected by FDR.

### Serum Protein Extraction, Digestion, and TMT Labeling

Thawed serum samples were used to remove high abundance proteins by using CaptureSelect™ MultiSpecies albumin depletion product (Thermo Fisher Scientific, MA, United States), followed the instructions of the manufacturer, and the protein solution was then collected. A 3KD ultrafiltration tube was used to concentrate the sample to an appropriate volume. The total protein was extracted by using an 8 M urea solution with protease inhibitors. Pierce BCA protein assay kit (Thermo Fisher Scientific, MA, United States) was used to quantify protein. Microplate reader (Thermo Fisher Scientific, MA, United States) was used to detect the OD value at 562 nm. A 10-μg protein from each sample was performed to sodium dodecyl sulfate-polyacrylamide gel electrophoresis (SDS-PAGE). Vertical electrophoresis (Liuyi Biological Technology Co., Ltd., Beijing, China) was used for protein separation at 100 V within 3 h.

A protein solution with 100 μg of protein was added in a 2-ml centrifuged tube, which was added with lysis buffer to 90 μl. Tris (2-carboxyethyl) phosphine (TCEP) reducing agent with a final concentration of 10 mmol/L was added, which was reacted at 37°C for 60 min. Iodoacetamide with a final concentration of 40 mmol/L was added and reacted at ambient temperature for 40 min. The precooled acetone (acetone: sample = 6:1) was added and precipitated at −20°C for 4 h. After centrifugation at 10,000 × *g* for 20 min, the precipitate was collected. After addition of 100 μl of 50 mmol/L tetraethyl-ammonium bromide (TEAB) to dissolve thoroughly, trypsin was added to enzymatically hydrolyze at 37°C overnight.

A 10-plex TMT reagent (Art. No. 90111, Thermo Fisher Scientific, MA, United States) was used in TMT labeling. One unit of TMT reagent was thawed and redissolved in 50 μl of acetonitrile. After tagging for 2 h at ambient temperature, hydroxylamine was added to react for 15 min. All labeled products in equal quantities were mixed, desalted, and vacuum dried (Bathla et al., 2020).

#### High pH RPLC Separation and LC-MS/MS Analysis

The peptide samples were redissolved with ultra-performance liquid chromatography (Waters, Milford, CT, United States) loading buffer, and then fractionated by using ACQUITY UPLC BEH C18 Column (1.7 μm, 2.1 × 150 mm, Waters, Milford, CT, United States). Phase A was 2% acetonitrile, and phase B was 80% acetonitrile. Elution gradient: 0–2 min, 100% A; 2–17 min, 0–3.8% B; 17–35 min, 3.8–24% B; 35–38 min, 24–30% B; 38–39 min, 30–43% B; 39–40 min, 43–100% B; 40–46 min, 100%–0 B. The UV detection wavelength was 214 nm, and the flow rate was 200 μl/min. A total of 20 fractions were collected from each sample, which was combined into 10 fractions per sample.

Labeled peptides were analyzed by nano-upgraded liquid chromatography tandem mass spectrometry (Easy-nLC 1200 combined with Q Exactive mass spectrometer). The peptides were dissolved by mass spectrometry loading buffer and separated on C18 chromatographic column (75 μm × 25 cm, Thermo Fisher Scientific, MA, United States) for 120 min with a flow rate of 300 μl/min. Phase A was 2% acetonitrile, and phase B was 80% acetonitrile, which both contained 0.1% formic acid. EASY-nLC liquid phase gradient elution: 0–1 min, 0–5% B; 1–63 in, 5–23% B; 63–88 min, 23–48% B; 88–89 min, 48–100% B; 89–95 min, 100% B. The full-scan MS (m/z 350–1,300) and MS/MS (m/z 100) resolution were 70 and 35 K, respectively. The top 20 most intense precursor ions were selected for secondary fragmentation, and the dynamic elimination was 18 s (Bili et al., 2018).

### Protein Identification and Bioinformatics Analysis

The raw data were analyzed by using Proteome Discoverer software (version 2.2, Thermo Fisher Scientific, MA, United States) against UniProt database.^10^ The MS/MS search criteria were as follows: mass tolerance of 10 ppm for MS and 0.02 Da for MS/MS Tolerance, trypsin as the enzyme with two missed cleavage allowed, carbamido methylation of cysteine and the TMT of N-terminus and lysine side chains of peptides as fixed modification, and methionine oxidation as dynamic modifications, respectively. False discovery rate of peptide identification was set as FDR ≤ 0.01. A minimum of one unique peptide identification was used to support protein identification. Student’s *t*-test in R was used to calculate *p*-value and FC. Significant differentially expressed proteins with corrected *p* < 0.05 and FC > 1.2 were upregulated proteins, and those with corrected *p* < 0.05 and FC < 0.83 were downregulated proteins.

Gene ontology (GO)^11,^^12^ was used to perform functional annotation of all proteins. The Kyoto Encyclopedia of Genes and Genomes (KEGG)^13^ pathway database was used to analyze the metabolic pathways involved in differential proteins.

### Statistical Analyses

Data of basic information of SCM cows (milk SCC, the result of CMT, average dry matter intake, days in milk, parity, and milk yield), the concentrations of serum lipids and fecal fermentation parameters were analyzed by one-way ANOVA in SPSS software (version 22.0, IBM, Chicago, IL, United States). Least significance difference (LSD) method was used for multiple comparisons of means among the five inulin groups. Significant difference and statistical tendency were set at *p* < 0.05 and 0.05 ≤ *p* < 0.1.

## Data Availability Statement

The datasets presented in this study can be found in online repositories. The names of the repository/repositories and accession number(s) can be found below: https://www.ncbi.nlm.nih.gov/, BioProject ID: PRJNA760802.

## Ethics Statement

The animal study was reviewed and approved by the Institute of Animal Science and Veterinary Medicine, Chinese Academy of Agricultural Sciences (Beijing, China; approval number: IAS-2020-92). Written informed consent was obtained from the owners for the participation of their animals in this study.

## Author Contributions

YW designed, performed the experiments, and drafted the manuscript. HW, FZ, and DH assisted in the sampling. JL provided the inulin. XN and YZ participated in the editing of the manuscript. LJ provided the sample-detecting instruments. BX, JY, and LY were involved in the conception of the study and manuscript. All authors read and approved the final manuscript.

## Conflict of Interest

The authors declare that the research was conducted in the absence of any commercial or financial relationships that could be construed as a potential conflict of interest.

## Publisher’s Note

All claims expressed in this article are solely those of the authors and do not necessarily represent those of their affiliated organizations, or those of the publisher, the editors and the reviewers. Any product that may be evaluated in this article, or claim that may be made by its manufacturer, is not guaranteed or endorsed by the publisher.

## Footnotes

1
http://www.usadellab.org/cms/index.php?page=trimmomatic

2
https://ccb.jhu.edu/software/FLASH/index.shtml

3
http://www.drive5.com/uparse/

4
https://sourceforge.net/projects/rdp-classifier/

5
http://www.arb-silva.de

6
https://www.mothur.org/wiki/Download_mothur

7
http://qiime.org/install/index.html

8
http://huttenhower.sph.harvard.edu/galaxy/root?tool_id=lefse_upload

9
http://www.hmdb.ca/

10
https://www.uniprot.org/

11
http://www.blast2go.com/b2ghome

12
http://geneontology.org/

13
http://www.genome.jp/kegg//
