# Supplementary material for: Changes in the Profile of Fecal Microbiota and Metabolites as Well as Serum Metabolites and Proteome After Dietary Inulin Supplementation in Dairy Cows With Subclinical Mastitis
Source: Front Microbiol. 2022 Apr 4;13:809139. doi: 10.3389/fmicb.2022.809139 (PMC9037088; doi:10.3389/fmicb.2022.809139)
Supplement: Supplementary file 1 [file Data_Sheet_1.docx]

**Table S1** Ingredients and chemical composition of basal diet (% DM)

| Ingredients | % | Chemical composition | % |
| --- | --- | --- | --- |
| Corn silage | 44.21 | DM, % of fresh | 48.0 |
| Alfalfa hey | 4.03 | CP | 16.5 |
| Oat grass | 2.30 | NDF | 30.0 |
| Alfalfa silage | 2.88 | ADF | 17.6 |
| Steam-flaked corn | 4.23 | EE | 5.30 |
| Corn | 8.99 | Ca | 0.21 |
| Corn husk of spray | 0.58 | P | 0.11 |
| Extruded soybean | 1.39 | Ash | 7.90 |
| Beet pellets | 2.56 | NEL, Mcal/kg DM | 1.74 |
| Brewer’s grains | 11.52 |  |  |
| DDGS | 0.58 |  |  |
| Cottonseed | 3.30 |  |  |
| Corn bran | 3.38 |  |  |
| Soya meal | 4.29 |  |  |
| Double-low rapeseed meal | 2.88 |  |  |
| Megalac | 0.66 |  |  |
| Fatty powder | 0.30 |  |  |
| 5% Premix | 1.92 |  |  |

DDGS, distillers dried grains with solubles.

Megalac, a rumen protected fatty acid calcium (VOLAC International Ltd., UK).

5% Premix, including (per kg of DM) 350 KIU of vitamin A, 150 KIU of vitamin D_3_, 680 IU of vitamin E, 700 mg of Cu, 3,000 mg of Zn, 50,000 mg of Fe, 24,000 mg of Mn, 10 mg of Se, 100 mg of I, 20 mg of Co, ≥ 14.5 % of Ca and ≥ 2.5 % of P.

EE = Ether extract.

**Table S2** Effects of inulin addition on α-abundance of ruminal microbiota

| **Items** | **Groups (n = 8)** | | | | | **SEM** | ***P*-value** |
| --- | --- | --- | --- | --- | --- | --- | --- |
|  | **Con** | **Inu_1** | **Inu_2** | **Inu_3** | **Inu_4** |  |  |
| Shannon | 4.61^c^ | 4.67^c^ | 4.84^b^ | 5.16^a^ | 5.12^a^ | 0.059 | 0.007 |
| Simpson | 0.013^b^ | 0.017^b^ | 0.028^ab^ | 0.032^a^ | 0.028^ab^ | 0.002 | 0.016 |
| Chao1 | 971^b^ | 981^b^ | 1026^ab^ | 1077^a^ | 1060^ab^ | 15.8 | 0.047 |
| Ace | 977 | 1008 | 1005 | 1090 | 1059 | 15.9 | 0.058 |
| Coverage | 0.99 | 0.99 | 0.99 | 0.99 | 0.99 | 0.00019 | 0.760 |

Con = control group; Con = control group; Inu_1 = inulin_1 group, the inulin addition level was 100 g/d per cow; Inu_2 = inulin_2 group, the inulin addition level was 200 g/d per cow; Inu_3 = inulin_3 group, the inulin addition level was 300 g/d per cow; Inu_4 = inulin_4 group, the inulin addition level was 400 g/d per cow; ^a, b, c^ = within a row, different letters differed significantly (*P* < 0.05).

**Table S3** Effects of inulin on the relative abundances of fecal microbiota at phylum level.

| **NO.** | **Items** | **Groups (n = 8)** | | | | | **SEM** | ***P*-value** | **Corrected *P*-value** |
| --- | --- | --- | --- | --- | --- | --- | --- | --- | --- |
|  |  | **Con** | **Inu_1** | **Inu_2** | **Inu_3** | **Inu_4** |  |  |  |
| 1 | Firmicutes | 66.0^a^ | 64.6^a^ | 58.5^b^ | 52.5^b^ | 59.9^b^ | 1.07 | 0.013 | 0.047 |
| 2 | Bacteroidota | 26.0^c^ | 25.8^c^ | 30.7^b^ | 36.6^a^ | 30.8^b^ | 0.89 | 0.017 | 0.048 |
| 3 | Actinobacteriota | 5.78 | 6.47 | 7.33 | 7.46 | 6.42 | 0.140 | 0.022 | 0.051 |
| 4 | Spirochaetota | 1.06 | 2.36 | 2.78 | 2.36 | 1.97 | 0.130 | 0.292 | 0.341 |
| 5 | Patescibacteria | 0.67 | 0.44 | 0.25 | 0.46 | 0.40 | 0.030 | 0.002 | 0.028 |
| 6 | Proteobacteria | 0.20 | 0.11 | 0.10 | 0.17 | 0.24 | 0.012 | 0.062 | 0.124 |
| 7 | unclassified_k__norank_d__Bacteria | 0.15 | 0.10 | 0.23 | 0.03 | 0.17 | 0.015 | 0.089 | 0.156 |
| 8 | Cyanobacteria | 0.13 | 0.09 | 0.04 | 0.26 | 0.01 | 0.020 | 0.005 | 0.023 |
| 9 | Verrucomicrobiota | 0.02 | 0.03 | 0.04 | 0.11 | 0.02 | 0.008 | 0.089 | 0.138 |
| 10 | Fibrobacterota | 0.02 | 0.01 | 0.03 | 0.02 | 0.01 | 0.002 | 0.880 | 0.880 |
| 11 | Desulfobacterota | 0.01 | 0.01 | 0.00 | 0.03 | 0.01 | 0.002 | 0.117 | 0.164 |
| 12 | Elusimicrobiota | 0.00 | 0.00 | 0.00 | 0.02 | 0.00 | 0.002 | 0.003 | 0.021 |
| 13 | Synergistota | 0.00 | 0.00 | 0.00 | 0.00 | 0.00 | 0.000 | 0.186 | 0.237 |
| 14 | Chloroflexi | 0.00 | 0.00 | 0.00 | 0.00 | 0.00 | 0.000 | 0.567 | 0.611 |

Con = control group; Inu_1 = inulin_1 group, the inulin addition level was 100 g/d per cow; Inu_2 = inulin_2 group, the inulin addition level was 200 g/d per cow; Inu_3 = inulin_3 group, the inulin addition level was 300 g/d per cow; Inu_4 = inulin_4 group, the inulin addition level was 400 g/d per cow; ^a, b, c^ = within a row, different letters differed significantly (Corrected *P* < 0.05)

**Table S4** Effects of inulin on the relative abundances of fecal microbiota at genus level (Top 100).

| **NO.** | **Items** | **Groups (n = 8)** | | | | | **SEM** | ***P*-value** | **Corrected *P*-value** |
| --- | --- | --- | --- | --- | --- | --- | --- | --- | --- |
|  |  | **Con** | **Inu_1** | **Inu_2** | **Inu_3** | **Inu_4** |  |  |  |
| 1 | *UCG-005* | 13.5 | 14.0 | 13.6 | 12.2 | 14.3 | 0.16 | 0.2568 | 0.377 |
| 2 | *Rikenellaceae_RC9_gut_group* | 9.25 | 9.03 | 9.12 | 8.57 | 9.73 | 0.083 | 0.1270 | 0.235 |
| 3 | *Paeniclostridium* | 9.83^a^ | 4.36^b^ | 6.37^b^ | 2.86^c^ | 5.39^b^ | 0.576 | 0.0013 | 0.034 |
| 4 | *norank_f__Eubacterium_coprostanoligenes_group* | 4.73 | 5.46 | 4.09 | 6.20 | 4.99 | 0.158 | 0.0499 | 0.144 |
| 5 | *Bifidobacterium* | 3.73^c^ | 4.21^c^ | 5.01^b^ | 6.20^a^ | 6.18^a^ | 0.225 | 0.0031 | 0.032 |
| 6 | *Romboutsia* | 2.15^d^ | 3.06^c^ | 3.09^c^ | 5.58^a^ | 4.65^b^ | 0.276 | 0.0038 | 0.040 |
| 7 | *norank_f__UCG-010* | 4.73 | 5.46 | 3.09 | 4.20 | 3.99 | 0.176 | 0.0499 | 0.144 |
| 8 | *Prevotellaceae_UCG-003* | 3.39 | 3.00 | 4.27 | 4.24 | 3.71 | 0.110 | 0.5425 | 0.606 |
| 9 | *Christensenellaceae_R-7_group* | 2.69 | 3.01 | 3.19 | 3.75 | 2.75 | 0.085 | 0.3991 | 0.498 |
| 10 | *norank_f__Muribaculaceae* | 2.80 | 2.75 | 3.39 | 3.08 | 2.63 | 0.061 | 0.3291 | 0.437 |
| 11 | *Monoglobus* | 2.60^b^ | 2.90^b^ | 4.68^a^ | 4.37^a^ | 2.67^b^ | 0.195 | 0.0046 | 0.042 |
| 12 | *Alistipes* | 1.97^c^ | 2.30^b^ | 2.24^b^ | 3.50^a^ | 3.39^a^ | 0.143 | 0.0044 | 0.046 |
| 13 | *unclassified_f__Lachnospiraceae* | 2.72 | 2.28 | 2.25 | 1.04 | 1.09 | 0.153 | 0.0945 | 0.195 |
| 14 | *Treponema* | 1.05 | 2.35 | 1.96 | 1.32 | 2.76 | 0.141 | 0.1478 | 0.252 |
| 15 | *Clostridium_sensu_stricto_1* | 2.50 | 1.63 | 2.82 | 0.89 | 1.88 | 0.151 | 0.0062 | 0.051 |
| 16 | *Bacteroides* | 0.89 | 2.46 | 0.52 | 0.76 | 1.57 | 0.158 | 0.0529 | 0.149 |
| 17 | *Turicibacter* | 1.48 | 1.10 | 2.09 | 1.06 | 1.46 | 0.083 | 0.0362 | 0.122 |
| 18 | *norank_f__norank_o__Clostridia_UCG-014* | 2.05 | 1.57 | 0.80 | 1.55 | 1.14 | 0.095 | 0.0261 | 0.099 |
| 19 | *norank_f__Bacteroidales_RF16_group* | 0.93 | 1.11 | 1.11 | 1.13 | 0.95 | 0.019 | 0.1600 | 0.267 |
| 20 | *norank_f__norank_o__Clostridia_vadinBB60_group* | 0.67 | 1.25 | 0.74 | 1.28 | 1.00 | 0.056 | 0.0062 | 0.051 |
| 21 | *Lachnospiraceae_NK4A136_group* | 0.94 | 0.90 | 0.90 | 1.00 | 0.86 | 0.010 | 0.8211 | 0.850 |
| 22 | *Prevotellaceae_UCG-001* | 0.74 | 0.59 | 0.77 | 0.89 | 1.37 | 0.060 | 0.5043 | 0.585 |
| 23 | *norank_f__Ruminococcaceae* | 1.43^a^ | 0.73^b^ | 1.11^a^ | 0.56^c^ | 0.95^b^ | 0.055 | 0.0051 | 0.047 |
| 24 | *Lachnospiraceae_AC2044_group* | 0.76^b^ | 0.82^b^ | 1.16^ab^ | 1.62^a^ | 1.47^a^ | 0.046 | 0.0037 | 0.040 |
| 25 | *Ruminococcus_torques_group* | 0.82 | 0.91 | 0.71 | 0.82 | 0.84 | 0.015 | 0.7255 | 0.771 |
| 26 | *Lachnospiraceae_NK3A20_group* | 0.63^c^ | 0.62^c^ | 1.08^b^ | 1.46^a^ | 1.19^b^ | 0.092 | 0.0003 | 0.034 |
| 27 | *Coprococcus* | 1.44^a^ | 1.42^a^ | 0.91^b^ | 0.69^c^ | 1.18^b^ | 0.054 | 0.0020 | 0.039 |
| 28 | *unclassified_f__Prevotellaceae* | 0.71 | 0.71 | 1.11 | 0.47 | 0.73 | 0.067 | 0.0084 | 0.054 |
| 29 | *g__Prevotella* | 0.55 | 0.42 | 0.85 | 0.70 | 0.60 | 0.032 | 0.1715 | 0.281 |
| 30 | *Family_XIII_AD3011_group* | 0.58 | 0.70 | 0.62 | 0.71 | 0.48 | 0.019 | 0.1611 | 0.267 |
| 31 | *Ruminococcus* | 0.54 | 0.57 | 0.59 | 0.74 | 0.61 | 0.015 | 0.2960 | 0.412 |
| 32 | *norank_f__norank_o__RF39* | 0.64 | 0.66 | 0.30 | 0.66 | 0.60 | 0.031 | 0.0675 | 0.171 |
| 33 | *UCG-009* | 0.46 | 0.58 | 0.60 | 0.73 | 0.46 | 0.022 | 0.1442 | 0.248 |
| 34 | *Candidatus_Saccharimonas* | 0.67 | 0.44 | 0.40 | 0.45 | 0.25 | 0.030 | 0.0105 | 0.058 |
| 35 | *Prevotellaceae_UCG-004* | 0.31 | 0.42 | 0.17 | 0.71 | 0.57 | 0.043 | 0.0341 | 0.119 |
| 36 | *unclassified_f__Ruminococcaceae* | 0.39 | 0.36 | 0.39 | 0.45 | 0.35 | 0.008 | 0.2063 | 0.320 |
| 37 | *UCG-002* | 0.35 | 0.30 | 0.47 | 0.43 | 0.36 | 0.014 | 0.0946 | 0.195 |
| 38 | *unclassified_o__Bacteroidales* | 0.25^c^ | 0.31^bc^ | 0.41^b^ | 0.62^a^ | 0.46^b^ | 0.029 | 0.0035 | 0.040 |
| 39 | *unclassified_o__Oscillospirales* | 0.71 | 0.64 | 0.47 | 0.33 | 0.21 | 0.037 | 0.0081 | 0.054 |
| 40 | *NK4A214_group* | 0.23 | 0.46 | 0.34 | 0.49 | 0.29 | 0.022 | 0.0008 | 0.034 |
| 41 | *unclassified_c__Clostridia* | 0.78^a^ | 0.73^a^ | 0.60^b^ | 0.21^c^ | 0.30^c^ | 0.051 | 0.0009 | 0.034 |
| 42 | *norank_f__p-2534-18B5_gut_group* | 0.48 | 0.74 | 0.47 | 0.26 | 0.31 | 0.037 | 0.0211 | 0.087 |
| 43 | *norank_f__Oscillospiraceae* | 0.70 | 0.78 | 0.25 | 0.21 | 0.23 | 0.018 | 0.0177 | 0.079 |
| 44 | *Cellulosilyticum* | 0.38 | 0.25 | 0.46 | 0.16 | 0.30 | 0.023 | 0.0453 | 0.140 |
| 45 | *Eubacterium_brachy_group* | 0.40 | 0.30 | 0.38 | 0.23 | 0.20 | 0.018 | 0.0202 | 0.086 |
| 46 | *Oscillibacter* | 0.24 | 0.23 | 0.36 | 0.39 | 0.22 | 0.017 | 0.0334 | 0.119 |
| 47 | *Eubacterium_nodatum_group* | 0.30 | 0.24 | 0.37 | 0.31 | 0.18 | 0.015 | 0.1083 | 0.214 |
| 48 | *Candidatus_Soleaferrea* | 0.24 | 0.28 | 0.22 | 0.28 | 0.19 | 0.008 | 0.3281 | 0.437 |
| 49 | *norank_f__norank_o__Bacteroidales* | 0.16 | 0.25 | 0.18 | 0.12 | 0.18 | 0.030 | 0.1070 | 0.214 |
| 50 | *Blautia* | 0.23 | 0.28 | 0.14 | 0.26 | 0.19 | 0.011 | 0.0607 | 0.160 |
| 51 | *unclassified_f__Peptostreptococcaceae* | 0.26^a^ | 0.23^a^ | 0.27^a^ | 0.02^c^ | 0.14^b^ | 0.021 | 0.0040 | 0.040 |
| 52 | *Dorea* | 0.09 | 0.19 | 0.17 | 0.24 | 0.22 | 0.012 | 0.0119 | 0.058 |
| 53 | *norank_f__Barnesiellaceae* | 0.16 | 0.16 | 0.27 | 0.18 | 0.15 | 0.010 | 0.2114 | 0.324 |
| 54 | *Alloprevotella* | 0.14 | 0.17 | 0.14 | 0.28 | 0.17 | 0.011 | 0.4390 | 0.527 |
| 55 | *Pygmaiobacter* | 0.15 | 0.19 | 0.17 | 0.16 | 0.17 | 0.003 | 0.8970 | 0.909 |
| 56 | *Ruminiclostridium* | 0.18 | 0.15 | 0.06 | 0.21 | 0.22 | 0.013 | 0.0071 | 0.052 |
| 57 | *Phascolarctobacterium* | 0.02 | 0.11 | 0.09 | 0.47 | 0.08 | 0.036 | 0.0068 | 0.052 |
| 58 | *norank_f__p-251-o5* | 0.14 | 0.11 | 0.14 | 0.15 | 0.39 | 0.029 | 0.0478 | 0.144 |
| 59 | *Agathobacter* | 0.14 | 0.16 | 0.14 | 0.12 | 0.15 | 0.003 | 0.5683 | 0.626 |
| 60 | *Eubacterium_siraeum_group* | 0.08 | 0.13 | 0.12 | 0.22 | 0.15 | 0.010 | 0.0112 | 0.058 |
| 61 | *Frisingicoccus* | 0.15 | 0.13 | 0.13 | 0.15 | 0.14 | 0.003 | 0.8134 | 0.845 |
| 62 | *unclassified_k__norank_d__Bacteria* | 0.15 | 0.10 | 0.17 | 0.03 | 0.23 | 0.015 | 0.0482 | 0.144 |
| 63 | *norank_f__Paludibacteraceae* | 0.06 | 0.14 | 0.17 | 0.17 | 0.15 | 0.010 | 0.0118 | 0.058 |
| 64 | *Ruminococcus_gauvreauii_group* | 0.23 | 0.16 | 0.14 | 0.17 | 0.16 | 0.007 | 0.0141 | 0.074 |
| 65 | *unclassified_f__Oscillospiraceae* | 0.10 | 0.13 | 0.13 | 0.22 | 0.10 | 0.010 | 0.0122 | 0.058 |
| 66 | *Olsenella* | 0.22 | 0.30 | 0.21 | 0.09 | 0.04 | 0.016 | 0.0774 | 0.176 |
| 67 | *dgA-11_gut_group* | 0.16 | 0.13 | 0.06 | 0.07 | 0.09 | 0.017 | 0.0129 | 0.060 |
| 68 | *Lachnospiraceae_UCG-010* | 0.07 | 0.07 | 0.12 | 0.25 | 0.07 | 0.015 | 0.0247 | 0.095 |
| 69 | *Marvinbryantia* | 0.10 | 0.16 | 0.06 | 0.12 | 0.13 | 0.008 | 0.0619 | 0.161 |
| 70 | *norank_f_norank_o__Izemoplasmatales* | 0.07 | 0.11 | 0.08 | 0.17 | 0.11 | 0.008 | 0.0319 | 0.116 |
| 71 | *Lachnospiraceae_FCS020_group* | 0.07 | 0.09 | 0.13 | 0.13 | 0.12 | 0.005 | 0.1083 | 0.214 |
| 72 | *norank_o_Gastranaerophilales* | 0.13 | 0.09 | 0.11 | 0.26 | 0.04 | 0.019 | 0.0012 | 0.034 |
| 73 | *Acetitomaculum* | 0.14 | 0.13 | 0.13 | 0.17 | 0.06 | 0.008 | 0.0120 | 0.058 |
| 74 | *Intestinibacter* | 0.13 | 0.13 | 0.22 | 0.12 | 0.10 | 0.017 | 0.0006 | 0.034 |
| 75 | *norank_f__Peptococcaceae* | 0.17 | 0.20 | 0.15 | 0.10 | 0.07 | 0.010 | 0.1374 | 0.241 |
| 76 | *Anaerovibrio* | 0.08 | 0.07 | 0.16 | 0.08 | 0.07 | 0.008 | 0.0725 | 0.171 |
| 77 | *Prevotellaceae_Ga6A1_group* | 0.06 | 0.10 | 0.11 | 0.09 | 0.12 | 0.006 | 0.1616 | 0.267 |
| 78 | *Anaeroplasma* | 0.07 | 0.09 | 0.08 | 0.06 | 0.08 | 0.004 | 0.0734 | 0.171 |
| 79 | *UCG-004* | 0.06 | 0.09 | 0.07 | 0.05 | 0.04 | 0.004 | 0.0536 | 0.149 |
| 80 | *Eubacterium_ruminantium_group* | 0.07 | 0.05 | 0.06 | 0.11 | 0.06 | 0.005 | 0.0167 | 0.075 |
| 81 | *Anaerosporobacter* | 0.04 | 0.05 | 0.14 | 0.07 | 0.04 | 0.009 | 0.0032 | 0.040 |
| 82 | *norank_o_norank_c_Clostridia* | 0.14 | 0.17 | 0.16 | 0.11 | 0.05 | 0.006 | 0.0208 | 0.087 |
| 83 | *EMP-G18* | 0.05 | 0.08 | 0.07 | 0.05 | 0.07 | 0.003 | 0.3208 | 0.431 |
| 84 | *Succinivibrio* | 0.07 | 0.04 | 0.15 | 0.05 | 0.01 | 0.010 | 0.0085 | 0.054 |
| 85 | *Defluviitaleaceae_UCG-011* | 0.05 | 0.06 | 0.05 | 0.08 | 0.07 | 0.002 | 0.0551 | 0.149 |
| 86 | *norank_f__F082* | 0.10 | 0.07 | 0.07 | 0.09 | 0.06 | 0.005 | 0.0837 | 0.182 |
| 87 | *Negativibacillus* | 0.06 | 0.05 | 0.09 | 0.06 | 0.03 | 0.005 | 0.1295 | 0.238 |
| 88 | *Anaerorhabdus_furcosa_group* | 0.05 | 0.06 | 0.05 | 0.05 | 0.05 | 0.001 | 0.9723 | 0.972 |
| 89 | *Parabacteroides* | 0.09 | 0.07 | 0.05 | 0.06 | 0.05 | 0.004 | 0.0269 | 0.100 |
| 90 | *Mogibacterium* | 0.07 | 0.08 | 0.05 | 0.03 | 0.03 | 0.004 | 0.0499 | 0.144 |
| 91 | *Family_XIII_UCG-001* | 0.06 | 0.03 | 0.07 | 0.05 | 0.03 | 0.003 | 0.0553 | 0.149 |
| 92 | *Clostridium_sensu_stricto_6* | 0.13 | 0.12 | 0.08 | 0.02 | 0.07 | 0.006 | 0.2251 | 0.338 |
| 93 | *Eubacterium_hallii_group* | 0.05 | 0.04 | 0.05 | 0.04 | 0.03 | 0.002 | 0.4384 | 0.527 |
| 94 | *norank_f__Erysipelatoclostridiaceae* | 0.03 | 0.06 | 0.03 | 0.05 | 0.04 | 0.003 | 0.0092 | 0.056 |
| 95 | *Akkermansia* | 0.02 | 0.03 | 0.02 | 0.09 | 0.03 | 0.006 | 0.7576 | 0.801 |
| 96 | *Howardella* | 0.07 | 0.07 | 0.03 | 0.02 | 0.02 | 0.004 | 0.0214 | 0.087 |
| 97 | *Eisenbergiella* | 0.02 | 0.05 | 0.02 | 0.04 | 0.04 | 0.002 | 0.0880 | 0.188 |
| 98 | *Saccharofermentans* | 0.03 | 0.04 | 0.04 | 0.05 | 0.02 | 0.003 | 0.0344 | 0.119 |
| 99 | *Syntrophococcus* | 0.05 | 0.04 | 0.06 | 0.03 | 0.01 | 0.003 | 0.0573 | 0.152 |
| 100 | *DNF00809* | 0.04 | 0.04 | 0.04 | 0.08 | 0.06 | 0.002 | 0.2741 | 0.392 |
|  | Others | 4.46^a^ | 4.75^a^ | 3.42^b^ | 3.50^b^ | 3.04^b^ | 0.177 | 0.0147 | 0.055 |

Con = control group; Inu_1 = inulin_1 group, the inulin addition level was 100 g/d per cow; Inu_2 = inulin_2 group, the inulin addition level was 200 g/d per cow; Inu_3 = inulin_3 group, the inulin addition level was 300 g/d per cow; Inu_4 = inulin_4 group, the inulin addition level was 400 g/d per cow; ^a, b, c^ = within a row, different letters differed significantly (Corrected *P* < 0.05).

Table S5 Significantly different metabolites based on VIP value (top 30) between control and inulin_1 groups in feces sample of SCM cows

| **Metabolite** | **Formula** | **Mode** | **Retention time** | **M/Z** | **VIP** | **FC** | ***P*-value** | **Corrected**  ***P*-value** | **HMDB Superclass** | **HMDB Class** | **HMDB Subclass** |
| --- | --- | --- | --- | --- | --- | --- | --- | --- | --- | --- | --- |
| Spinosin A | C_39_H_42_O_19_ | neg | 5.05 | 795.21 | 4.32 | 0.69 | 0.001 | 0.042 | Phenylpropanoids and polyketides | Flavonoid glycosides | Flavonoids |
| Disialyllactose | C_34_H_56_N_2_O_27_ | neg | 3.84 | 959.28 | 4.15 | 0.71 | 0.001 | 0.047 | Organic oxygen compounds | Carbohydrates and carbohydrate conjugates | Organooxygen compounds |
| 4Alpha-hydroxymethyl-5alpha-cholesta-8-en-3beta-ol | C_28_H_48_O_2_ | pos | 5.79 | 458.40 | 3.88 | 1.28 | 0.001 | 0.048 | Lipids and lipid-like molecules | Cholestane steroids | Steroids and steroid derivatives |
| Vitisin C | C_56_H_42_O_12_ | neg | 5.70 | 905.26 | 3.49 | 1.19 | 6.29 × 10^-6^ | 0.003 | Phenylpropanoids and polyketides | Not Available | 2-arylbenzofuran flavonoids |
| Homoarecoline | C_9_H_15_NO_2_ | neg | 3.60 | 214.11 | 3.21 | 0.56 | 7.69 × 10^-9^ | 3.09 ×10^-5^ | Alkaloids and derivatives | Not Available | Not Available |
| Lactapiperanol D | C_18_H_28_O_5_ | neg | 3.05 | 345.17 | 2.88 | 0.88 | 1.90 × 10^-4^ | 0.018 | Lipids and lipid-like molecules | Diterpenoids | Prenol lipids |
| Ethyl glucuronide | C_8_H_14_O_7_ | neg | 2.94 | 203.06 | 2.75 | 0.81 | 2.41 × 10^-4^ | 0.020 | Organic oxygen compounds | Carbohydrates and carbohydrate conjugates | Organooxygen compounds |
| Polysorbate 20 | C_26_H_50_O_10_ | pos | 5.80 | 564.38 | 2.42 | 0.63 | 1.27 × 10^-5^ | 0.004 | Lipids and lipid-like molecules | Fatty acid esters | Fatty Acyls |
| Melibiose | C_12_H_22_O_11_ | neg | 0.59 | 377.09 | 2.27 | 1.55 | 5.46 × 10^-5^ | 0.009 | Organic oxygen compounds | Organooxygen compounds | Carbohydrates and carbohydrate conjugates |
| 13(S)-HpODE | C_18_H_32_O_4_ | neg | 6.31 | 311.22 | 2.24 | 0.83 | 0.000 | 0.015 | - | - | - |
| L-glycyl-L-hydroxyproline | C_7_H_12_N_2_O_4_ | pos | 1.61 | 153.07 | 2.17 | 0.74 | 2.03 × 10^-5^ | 0.006 | Organic acids and derivatives | Amino acids, peptides, and analogues | Carboxylic acids and derivatives |
| N-(5-Methyl-3-oxohexyl) alanine | C_10_H_19_NO_3_ | pos | 1.36 | 202.14 | 2.17 | 1.06 | 1.20 × 10^-6^ | 0.001 | Organic acids and derivatives | Amino acids, peptides, and analogues | Carboxylic acids and derivatives |
| Withanolide | C_28_H_38_O_6_ | pos | 6.64 | 493.25 | 2.15 | 1.49 | 0.001 | 0.037 | Lipids and lipid-like molecules | Glycerophospholipids | Glycerophosphocholines |
| Erbstatin Analog | C_10_H_10_O_4_ | neg | 3.74 | 193.05 | 2.12 | 0.52 | 0.001 | 0.041 | - | - | - |
| (S)-ATPA | C_10_H_16_N_2_O_4_ | pos | 0.65 | 229.12 | 2.06 | 0.90 | 2.78 × 10^-5^ | 0.007 | - | - | - |
| Guanine | C_5_H_5_N_5_O | pos | 0.65 | 152.06 | 2.03 | 0.66 | 2.48 × 10^-5^ | 0.006 | Organoheterocyclic compounds | Purines and purine derivatives | Imidazopyrimidines |
| Coprocholic acid | C_27_H_46_O_5_ | neg | 6.39 | 449.33 | 2.02 | 1.39 | 0.001 | 0.041 | Lipids and lipid-like molecules | Steroids and steroid derivatives | Bile acids, alcohols and derivatives |
| Tetrahydroneopterin | C_9_H_15_N_5_O_4_ | pos | 6.66 | 515.24 | 2.00 | 1.35 | 0.001 | 0.042 | Organoheterocyclic compounds | Pteridines and derivatives | Pterins and derivatives |
| Pyroglutamic acid | C_5_H_7_NO_3_ | pos | 1.61 | 171.08 | 1.89 | 0.65 | 9.99 × 10^-5^ | 0.013 | Organic acids and derivatives | Amino acids, peptides, and analogues | Carboxylic acids and derivatives |
| 12-oxo-20-dihydroxy-leukotriene B4 | C_20_H_30_O_6_ | neg | 6.00 | 365.20 | 1.86 | 0.69 | 0.001 | 0.034 | Lipids and lipid-like molecules | Fatty Acyls | Eicosanoids |
| Deoxycholic acid | C_24_H_40_O_4_ | neg | 6.33 | 391.28 | 1.85 | 1.47 | 3.92 × 10^-5^ | 0.026 | Lipids and lipid-like molecules | Steroids and steroid derivatives | Bile acids, alcohols and derivatives |
| Sterebin C | C_20_H_32_O_5_ | neg | 6.36 | 373.20 | 1.83 | 1.04 | 8.87 × 10^-5^ | 0.012 | Lipids and lipid-like molecules | Sesquiterpenoids | Prenol lipids |
| L-Glutamate | C_5_H_9_NO_4_ | pos | 0.58 | 148.06 | 1.79 | 1.30 | 0.001 | 0.036 | - | - | - |
| Citrulline | C_6_H_13_N_3_O_3_ | pos | 0.57 | 176.10 | 1.79 | 0.66 | 1.97 × 10^-4^ | 0.018 | Organic acids and derivatives | Amino acids, peptides, and analogues | Carboxylic acids and derivatives |
| Methionyl-Proline | C_10_H_18_N_2_O_3_S | pos | 4.15 | 211.09 | 1.75 | 0.96 | 3.61 × 10^-4^ | 0.024 | Organic acids and derivatives | Amino acids, peptides, and analogues | Carboxylic acids and derivatives |
| Threoninyl-Glycine | C_6_H_12_N_2_O_4_ | pos | 0.57 | 159.08 | 1.72 | 0.97 | 1.23 × 10^-4^ | 0.015 | Organic acids and derivatives | Amino acids, peptides, and analogues | Carboxylic acids and derivatives |
| Miraxanthin-III | C_17_H_18_N_2_O_5_ | pos | 2.96 | 331.13 | 1.71 | 0.96 | 0.001 | 0.042 | - | - | - |
| Alpha-Amylcinnamyl formate | C_15_H_20_O_2_ | pos | 5.12 | 233.15 | 1.68 | 0.96 | 2.54 × 10^-4^ | 0.021 | Benzenoids | Not Available | Benzene and substituted derivatives |
| L-Hypoglycin A | C_7_H_11_NO_2_ | neg | 2.26 | 327.16 | 1.68 | 0.96 | 2.64 × 10^-4^ | 0.021 | Organic acids and derivatives | Amino acids, peptides, and analogues | Carboxylic acids and derivatives |
| 4-hydroxy-3-(3-hydroxy-1-phenylbutyl)-2H-chromen-2-one | C_19_H_18_O_4_ | neg | 4.43 | 347.07 | 1.68 | 0.96 | 1.36 × 10^-4^ | 0.015 | Phenylpropanoids and polyketides | Hydroxycoumarins | Coumarins and derivatives |

pos = positive ion mode; neg = negative ion mode; M/Z = charge-to-mass ratio; VIP = variable important in projection; FC = fold change; HMDB = human metabolome database; - = the compound classification was unknown.

Table S6 Significantly different metabolites based on VIP value (top 30) between control and inulin_2 groups in feces sample of SCM cows

| **Metabolite** | **Formula** | **Mode** | **Retention time** | **M/Z** | **VIP** | **FC** | **P-value** | **Corrected P-value** | **HMDB Superclass** | **HMDB Class** | **HMDB Subclass** |
| --- | --- | --- | --- | --- | --- | --- | --- | --- | --- | --- | --- |
| Melilotoside B | C41H68O12 | pos | 6.02 | 753.48 | 3.54 | 1.21 | 0.005 | 0.048 | Lipids and lipid-like molecules | Triterpenoids | Prenol lipids |
| S-(3-Methyl-2-butenyl) 2-methylpropanethioate | C9H16OS | pos | 5.97 | 190.13 | 3.32 | 0.79 | 0.004 | 0.043 | Organic acids and derivatives | Thioesters | Thiocarboxylic acids and derivatives |
| Homoarecoline | C9H15NO2 | neg | 3.60 | 214.11 | 3.31 | 0.85 | 4.60 ×10^-10^ | 1.97 ×10^-6^ | Not Available | Not Available | Alkaloids and derivatives |
| Melibiose | C12H22O11 | neg | 0.59 | 377.09 | 3.26 | 1.81 | 1.27 × 10^-5^ | 0.001 | Organic oxygen compounds | Organooxygen compounds | Carbohydrates and carbohydrate conjugates |
| Vitisin C | C56H42O12 | neg | 5.70 | 905.26 | 3.26 | 1.17 | 1.27 × 10^-5^ | 0.001 | Phenylpropanoids and polyketides | Not Available | 2-arylbenzofuran flavonoids |
| 6-Hydroxyetodolac | C17H21NO4 | pos | 4.09 | 321.18 | 3.18 | 0.81 | 0.003 | 0.031 | Organoheterocyclic compounds | Indolyl carboxylic acids and derivatives | Indoles and derivatives |
| Homodolicholide | C29H48O6 | pos | 6.67 | 531.30 | 3.11 | 0.87 | 1.23 × 10^-4^ | 0.004 | Organoheterocyclic compounds | 1-benzopyrans | Benzopyrans |
| Deoxycholic acid | C24H40O4 | neg | 6.33 | 391.28 | 3.10 | 1.52 | 3.36 × 10^-4^ | 0.008 | Lipids and lipid-like molecules | Steroids and steroid derivatives | Bile acids, alcohols and derivatives |
| Thr-Ile-OH | C16H22N2O7 | pos | 2.27 | 355.15 | 2.99 | 1.14 | 3.75 × 10^-5^ | 0.002 | - | - | - |
| Tetrahydroneopterin | C9H15N5O4 | pos | 6.66 | 515.24 | 2.99 | 1.52 | 0.001 | 0.013 | Organoheterocyclic compounds | Pteridines and derivatives | Pterins and derivatives |
| 12-oxo-20-dihydroxy-leukotriene B4 | C20H30O6 | neg | 6.00 | 365.20 | 2.85 | 0.63 | 3.02 × 10^-4^ | 0.008 | Lipids and lipid-like molecules | Fatty Acyls | Eicosanoids |
| Withanolide | C28H38O6 | pos | 6.64 | 493.25 | 2.79 | 1.58 | 3.22 × 10^-5^ | 0.002 | Lipids and lipid-like molecules | Glycerophospholipids | Glycerophosphocholines |
| 13(S)-HpODE | C18H32O4 | neg | 6.31 | 311.22 | 2.73 | 0.66 | 0.001 | 0.012 | - | - | - |
| L-Glutamate | C5H9NO4 | pos | 0.58 | 148.06 | 2.67 | 1.60 | 0.002 | 0.029 | - | - | - |
| Domoic acid | C15H21NO6 | neg | 4.70 | 356.13 | 2.66 | 1.09 | 3.08 × 10^-6^ | 2.95 ×10^-4^ | Organic acids and derivatives | Amino acids, peptides, and analogues | Carboxylic acids and derivatives |
| Aldosterone | C21H28O5 | pos | 3.10 | 343.19 | 2.66 | 1.10 | 2.67 × 10^-9^ | 3.90 ×10^-6^ | Lipids and lipid-like molecules | Hydroxysteroids | Steroids and steroid derivatives |
| Dicrocin | C32H44O14 | neg | 5.05 | 633.26 | 2.65 | 0.91 | 2.38 × 10^-4^ | 0.007 | Lipids and lipid-like molecules | Diterpenoids | Prenol lipids |
| Cer(d18:0/13:0) | C32H65NO3 | pos | 7.58 | 534.49 | 2.65 | 0.64 | 0.000 | 0.002 | Lipids and lipid-like molecules | Sphingolipids | Ceramides |
| 12,13-DiHODE | C18H32O4 | neg | 5.34 | 349.18 | 2.64 | 0.65 | 0.001 | 0.018 | Lipids and lipid-like molecules | Fatty Acyls | Lineolic acids and derivatives |
| Lactapiperanol D | C18H28O5 | neg | 3.05 | 345.17 | 2.57 | 0.89 | 0.001 | 0.020 | Lipids and lipid-like molecules | Diterpenoids | Prenol lipids |
| Indoleacrylic acid | C11H9NO2 | pos | 2.99 | 229.10 | 2.53 | 1.07 | 3.76 × 10^-7^ | 7.11 × 10^-5^ | Organoheterocyclic compounds | Indoles | Indoles and derivatives |
| Coprocholic acid | C27H46O5 | neg | 6.39 | 449.33 | 2.52 | 1.43 | 0.003 | 0.034 | Lipids and lipid-like molecules | Steroids and steroid derivatives | Bile acids, alcohols and derivatives |
| Cibaric acid | C18H28O5 | pos | 2.50 | 369.17 | 2.51 | 1.08 | 2.87 × 10^-8^ | 1.57 ×10^-5^ | Lipids and lipid-like molecules | Lineolic acids and derivatives | Fatty Acyls |
| Taurodeoxycholic acid | C26H45NO6S | pos | 8.39 | 464.28 | 2.50 | 1.37 | 1.23 × 10^-4^ | 0.004 | Lipids and lipid-like molecules | Steroids and steroid derivatives | Bile acids, alcohols and derivatives |
| 4-Methyl-5-thiazoleethanol | C6H9NOS | pos | 1.47 | 144.05 | 2.50 | 0.91 | 0.001 | 0.018 | - | - | - |
| Polysorbate 20 | C26H50O10 | pos | 5.80 | 564.38 | 2.46 | 0.94 | 0.000 | 0.000 | Lipids and lipid-like molecules | Fatty acid esters | Fatty Acyls |
| Rishitinol | C15H22O2 | pos | 2.21 | 257.15 | 2.42 | 0.90 | 0.001 | 0.014 | Benzenoids | Not Available | Tetralins |
| (1'R)-Nepetalic acid | C10H16O3 | neg | 5.07 | 183.10 | 2.42 | 1.08 | 0.000 | 0.001 | Lipids and lipid-like molecules | Monoterpenoids | Prenol lipids |
| (1beta,2beta,5beta)-p-Menth-3-ene-1,2,5-triol | C10H18O3 | pos | 1.50 | 204.16 | 2.40 | 1.09 | 0.000 | 0.000 | Lipids and lipid-like molecules | Monoterpenoids | Prenol lipids |
| Pseudouridine | C9H12N2O6 | pos | 3.32 | 245.07 | 2.37 | 0.91 | 0.000 | 0.004 | Nucleosides, nucleotides, and analogues | Not Available | Nucleoside and nucleotide analogues |

pos = positive ion mode; neg = negative ion mode; M/Z = charge-to-mass ratio; VIP = variable important in projection; FC = fold change; HMDB = human metabolome database; - = the compound classification was unknown.

Table S7 Significantly different metabolites based on VIP value (top 30) between control and inulin_3 groups in feces sample of SCM cows

| **Metabolite** | **Formula** | **Mode** | **Retention time** | **M/Z** | **VIP** | **FC** | **P-value** | **Corrected P-value** | **HMDB Superclass** | **HMDB Class** | **HMDB Subclass** |
| --- | --- | --- | --- | --- | --- | --- | --- | --- | --- | --- | --- |
| Oxypinnatanine | C10H16N2O6 | neg | 6.17 | 295.07 | 3.85 | 0.69 | 0.005 | 0.043 | Carboxylic acids and derivatives | Amino acids, peptides, and analogues | Organic acids and derivatives |
| Sintaxanthin | C31H42O | pos | 5.90 | 448.35 | 3.36 | 1.36 | 0.004 | 0.039 | Lipids and lipid-like molecules | Triterpenoids | Prenol lipids |
| Vitisin C | C56H42O12 | neg | 5.70 | 905.26 | 3.28 | 1.19 | 2.39×10^-6^ | 4.83×10^-4^ | Phenylpropanoids and polyketides | Not Available | 2-arylbenzofuran flavonoids |
| Lactapiperanol D | C18H28O5 | neg | 3.05 | 345.17 | 3.24 | 0.83 | 5.50×10^-6^ | 0.001 | Lipids and lipid-like molecules | Diterpenoids | Prenol lipids |
| Deoxycholic acid | C24H40O4 | neg | 6.33 | 391.28 | 3.18 | 1.65 | 0.006 | 0.032 | Lipids and lipid-like molecules | Steroids and steroid derivatives | Bile acids, alcohols and derivatives |
| Melibiose | C12H22O11 | neg | 0.59 | 377.09 | 3.15 | 1.78 | 8.83×10^-5^ | 0.004 | Organic oxygen compounds | Organooxygen compounds | Carbohydrates and carbohydrate conjugates |
| Taurodeoxycholic acid | C26H45NO6S | pos | 8.39 | 464.28 | 3.15 | 1.78 | 2.28×10^-6^ | 4.77×10^-4^ | Lipids and lipid-like molecules | Steroids and steroid derivatives | Bile acids, alcohols and derivatives |
| 12-oxo-20-dihydroxy-leukotriene B4 | C20H30O6 | neg | 6.00 | 365.20 | 3.14 | 0.51 | 4.93×10^-6^ | 0.001 | Lipids and lipid-like molecules | Fatty Acyls | Eicosanoids |
| 12,13-DiHODE | C18H32O4 | neg | 5.34 | 349.18 | 3.13 | 0.53 | 5.32×10^-5^ | 0.003 | Lipids and lipid-like molecules | Fatty Acyls | Lineolic acids and derivatives |
| 13(S)-HpODE | C18H32O4 | neg | 6.31 | 311.22 | 3.12 | 0.62 | 1.28×10^-5^ | 0.001 | - | - | - |
| Ethyl glucuronide | C8H14O7 | neg | 2.94 | 203.06 | 2.99 | 0.89 | 8.22×10^-7^ | 2.74 ×10^-4^ | Organooxygen compounds | Carbohydrates and carbohydrate conjugates | Organic oxygen compounds |
| HC Blue No.1 | C11H17N3O4 | pos | 1.57 | 256.13 | 2.97 | 1.14 | 2.23×10^-8^ | 3.36×10^-5^ | - | - | - |
| Tetrahydroneopterin | C9H15N5O4 | pos | 6.66 | 515.24 | 2.94 | 1.59 | 0.001 | 0.017 | Organoheterocyclic compounds | Pteridines and derivatives | Pterins and derivatives |
| Homoarecoline | C9H15NO2 | neg | 3.60 | 214.11 | 2.92 | 0.86 | 1.94E-09 | 5.53E-06 | Not Available | Not Available | Alkaloids and derivatives |
| Coprocholic acid | C27H46O5 | neg | 6.39 | 449.33 | 2.88 | 1.55 | 0.002 | 0.027 | Lipids and lipid-like molecules | Steroids and steroid derivatives | Bile acids, alcohols and derivatives |
| Cer(d18:0/13:0) | C32H65NO3 | pos | 7.58 | 534.49 | 2.85 | 0.61 | 0.001 | 0.014 | Lipids and lipid-like molecules | Sphingolipids | Ceramides |
| S-(3-Methyl-2-butenyl) 2-methylpropanethioate | C9H16OS | pos | 5.97 | 190.13 | 2.84 | 0.80 | 0.006 | 0.049 | Thiocarboxylic acids and derivatives | Thioesters | Organic acids and derivatives |
| 3-METHYLORSELLINIC ACID | C9H10O4 | neg | 2.55 | 181.05 | 2.82 | 0.88 | 0.001 | 0.017 | - | - | - |
| Triethyl phosphate | C6H15O4P | pos | 4.89 | 183.08 | 2.72 | 1.15 | 0.003 | 0.032 | - | - | - |
| Pyrophaeophorbide a | C33H34N4O3 | neg | 8.35 | 533.26 | 2.63 | 0.89 | 0.001 | 0.014 | Tetrapyrroles and derivatives | Chlorins | Organoheterocyclic compounds |
| N2-Acetylphenelzine | C10H14N2O | pos | 2.54 | 179.12 | 2.60 | 1.15 | 0.003 | 0.032 | - | - | - |
| L-Glutamate | C5H9NO4 | pos | 0.58 | 148.06 | 2.54 | 1.58 | 0.001 | 0.013 | - | - | - |
| Guanine | C5H5N5O | pos | 0.65 | 152.06 | 2.53 | 0.93 | 1.82×10^-6^ | 4.18×10^-4^ | Imidazopyrimidines | Purines and purine derivatives | Organoheterocyclic compounds |
| Polysorbate 20 | C26H50O10 | pos | 5.80 | 564.38 | 2.49 | 0.92 | 3.60×10^-7^ | 1.68 ×10^-4^ | Lipids and lipid-like molecules | Fatty acid esters | Fatty Acyls |
| N-Acetylornithine | C7H14N2O3 | pos | 1.32 | 216.13 | 2.33 | 1.10 | 4.72×10^-5^ | 0.003 | Organic acids and derivatives | Amino acids, peptides, and analogues | Carboxylic acids and derivatives |
| Indolelactic acid | C11H11NO3 | neg | 4.27 | 204.07 | 2.33 | 0.90 | 0.006 | 0.049 | Organoheterocyclic compounds | Indolyl carboxylic acids and derivatives | Indoles and derivatives |
| Deoxyinosine | C10H12N4O4 | neg | 1.20 | 251.08 | 2.31 | 0.94 | 0.000 | 0.004 | Nucleosides, nucleotides, and analogues | Purine 2'-deoxyribonucleosides | Purine nucleosides |
| 3-Deoxyguanosine | C10H13N5O4 | neg | 1.15 | 266.09 | 2.29 | 0.94 | 0.000 | 0.002 | - | - | - |
| (3R)-3,4-Dihydroxy-3-(hydroxymethyl) butanenitrile 4-glucoside | C11H19NO8 | pos | 0.76 | 276.11 | 2.27 | 0.93 | 0.000 | 0.001 | Lipids and lipid-like molecules | Glycosylglycerols | Glycerolipids |
| Homodolicholide | C29H48O6 | pos | 6.67 | 531.30 | 2.26 | 0.91 | 0.003 | 0.029 | Organoheterocyclic compounds | 1-benzopyrans | Benzopyrans |

pos = positive ion mode; neg = negative ion mode; M/Z = charge-to-mass ratio; VIP = variable important in projection; FC = fold change; HMDB = human metabolome database; - = the compound classification was unknown.

Table S8 Significantly different metabolites based on VIP value (top 30) between control and inulin_4 groups in feces sample of SCM cows

| **Metabolite** | **Formula** | **Mode** | **Retention time** | **M/Z** | **VIP** | **FC** | **P-value** | **Corrected P-value** | **HMDB Superclass** | **HMDB Class** | **HMDB Subclass** |
| --- | --- | --- | --- | --- | --- | --- | --- | --- | --- | --- | --- |
| Histidinyl-Proline | C11H16N4O3 | neg | 1.29 | 297.12 | 4.35 | 1.46 | 7.28×10^-7^ | 6.05×10^-5^ | Organic acids and derivatives | Amino acids, peptides, and analogues | Carboxylic acids and derivatives |
| Oxypinnatanine | C10H16N2O6 | neg | 6.17 | 295.07 | 3.85 | 0.69 | 0.005 | 0.033 | Organic acids and derivatives | Amino acids, peptides, and analogues | Carboxylic acids and derivatives |
| Ichangin 4-glucoside | C32H42O14 | neg | 5.05 | 649.25 | 3.54 | 0.79 | 2.53×10^-4^ | 0.004 | Lipids and lipid-like molecules | Terpene glycosides | Prenol lipids |
| 3-Hydroxy-carbofuran | C12H15NO4 | neg | 2.82 | 272.07 | 3.50 | 1.25 | 0.005 | 0.033 | Organoheterocyclic compounds | Not Available | Coumarans |
| Coprocholic acid | C27H46O5 | neg | 6.39 | 449.33 | 3.17 | 1.58 | 3.53×10^-6^ | 1.80×10^-4^ | Lipids and lipid-like molecules | Steroids and steroid derivatives | Bile acids, alcohols and derivatives |
| Vitisin C | C56H42O12 | neg | 5.70 | 905.26 | 3.13 | 1.22 | 0.003 | 0.021 | Phenylpropanoids and polyketides | Not Available | 2-arylbenzofuran flavonoids |
| Tetrahydroneopterin | C9H15N5O4 | pos | 6.66 | 515.24 | 2.97 | 1.51 | 0.001 | 0.008 | Organoheterocyclic compounds | Pteridines and derivatives | Pterins and derivatives |
| 13(S)-HpODE | C18H32O4 | neg | 6.31 | 311.22 | 2.89 | 0.66 | 1.27×10^-4^ | 0.002 | - | - | - |
| Melibiose | C12H22O11 | neg | 0.59 | 377.09 | 2.88 | 1.63 | 0.001 | 0.007 | Organic oxygen compounds | Organooxygen compounds | Carbohydrates and carbohydrate conjugates |
| Taurodeoxycholic acid | C26H45NO6S | pos | 8.39 | 464.28 | 2.83 | 1.61 | 0.002 | 0.015 | Lipids and lipid-like molecules | Steroids and steroid derivatives | Bile acids, alcohols and derivatives |
| D-erythro-L-galacto-Nonulose | C9H18O9 | pos | 1.20 | 253.09 | 2.71 | 0.88 | 1.78×10^-7^ | 2.61 ×10^-5^ | Organic oxygen compounds | Carbohydrates and carbohydrate conjugates | Organooxygen compounds |
| Formylfusarochromanone | C16H20N2O5 | pos | 5.36 | 285.12 | 2.69 | 0.86 | 4.65×10^-4^ | 0.006 | Organoheterocyclic compounds | 1-benzopyrans | Benzopyrans |
| Ganoderic acid Mj | C33H52O6 | pos | 7.56 | 567.36 | 2.65 | 0.89 | 0.001 | 0.007 | Lipids and lipid-like molecules | Triterpenoids | Prenol lipids |
| 12-oxo-20-dihydroxy-leukotriene B4 | C20H30O6 | neg | 6.00 | 365.20 | 2.64 | 0.65 | 6.42×10^-5^ | 0.001 | Lipids and lipid-like molecules | Fatty Acyls | Eicosanoids |
| Anhydrocinnzeylanine | C22H32O7 | neg | 6.41 | 443.19 | 2.62 | 0.88 | 0.001 | 0.011 | Lipids and lipid-like molecules | Terpene lactones | Prenol lipids |
| L-Methionine S-oxide | C5H11NO3S | pos | 0.58 | 166.05 | 2.60 | 0.89 | 3.14×10^-4^ | 0.005 | - | - | - |
| Dicrocin | C32H44O14 | neg | 5.05 | 633.26 | 2.59 | 0.90 | 1.05×10^-4^ | 0.002 | Lipids and lipid-like molecules | Diterpenoids | Prenol lipids |
| N-Acetylornithine | C7H14N2O3 | pos | 1.32 | 216.13 | 2.55 | 1.11 | 2.57×10^-6^ | 1.42 ×10^-4^ | Organic acids and derivatives | Amino acids, peptides, and analogues | Carboxylic acids and derivatives |
| 4-Methyl-5-thiazoleethanol | C6H9NOS | pos | 1.47 | 144.05 | 2.53 | 0.89 | 3.11E-04 | 0.005 | - | - | - |
| Pondaplin | C14H14O3 | pos | 1.47 | 294.11 | 2.46 | 1.08 | 2.66×10^-7^ | 3.25×10^-5^ | Organic oxygen compounds | Ethers | Organooxygen compounds |
| Cer(d18:0/13:0) | C32H65NO3 | pos | 7.58 | 534.49 | 2.44 | 0.71 | 0.002 | 0.021 | Lipids and lipid-like molecules | Sphingolipids | Ceramides |
| Aldosterone | C21H28O5 | pos | 3.10 | 343.19 | 2.42 | 1.10 | 2.07×10^-8^ | 6.56×10^-6^ | Lipids and lipid-like molecules | Hydroxysteroids | Steroids and steroid derivatives |
| 6-Hydroxyetodolac | C17H21NO4 | pos | 4.09 | 321.18 | 2.37 | 0.89 | 1.27×10^-4^ | 0.002 | Organoheterocyclic compounds | Indolyl carboxylic acids and derivatives | Indoles and derivatives |
| ()-3-Hydroxynonanoic acid | C9H18O3 | pos | 2.20 | 216.16 | 2.29 | 1.08 | 3.94×10^-9^ | 2.11 × 10^-6^ | Organic acids and derivatives | Medium-chain hydroxy acids and derivatives | Hydroxy acids and derivatives |
| Withanolide | C28H38O6 | pos | 6.64 | 493.25 | 2.27 | 1.48 | 0.005 | 0.033 | Lipids and lipid-like molecules | Glycerophospholipids | Glycerophosphocholines |
| 12,13-DiHODE | C18H32O4 | neg | 5.34 | 349.18 | 2.27 | 0.63 | 0.007 | 0.045 | Lipids and lipid-like molecules | Fatty Acyls | Lineolic acids and derivatives |
| L-glycyl-L-hydroxyproline | C7H12N2O4 | pos | 1.61 | 153.07 | 2.26 | 0.92 | 7.57×10^-8^ | 1.51×10^-5^ | Organic acids and derivatives | Amino acids, peptides, and analogues | Carboxylic acids and derivatives |
| Thymine | C5H6N2O2 | pos | 1.62 | 127.05 | 2.25 | 0.94 | 7.04×10^-8^ | 1.46×10^-5^ | Organoheterocyclic compounds | Pyrimidines and pyrimidine derivatives | Diazines |
| GW 9508 | C22H21NO3 | pos | 3.02 | 348.16 | 2.24 | 1.07 | 2.42×10^-8^ | 6.97×10^-6^ | - | - | - |
| Deoxycholic acid | C24H40O4 | neg | 6.33 | 391.28 | 2.24 | 1.66 | 4.84×10^-7^ | 4.66×10^-5^ | Lipids and lipid-like molecules | Steroids and steroid derivatives | Bile acids, alcohols and derivatives |

pos = positive ion mode; neg = negative ion mode; M/Z = charge-to-mass ratio; VIP = variable important in projection; FC = fold change; HMDB = human metabolome database; - = the compound classification was unknown.

Table S9 Significantly differential metabolites based on VIP value (top 30) between control and inulin_1 group in serum samples of SCM cows

| **Metabolite** | **Formula** | **Mode** | **Retention time** | **M/Z** | **VIP** | **FC** | ***P*-value** | **Corrected *P*-value** | **HMDB Superclass** | **HMDB Class** | **HMDB Subclass** |
| --- | --- | --- | --- | --- | --- | --- | --- | --- | --- | --- | --- |
| 12-HETE | C20H32O3 | neg | 6.33 | 319.23 | 4.01 | 0.60 | 2.90×10^-4^ | 0.003 | - | - | - |
| 12-Oxo-20-trihydroxy-leukotriene B4 | C20H30O7 | neg | 6.19 | 403.17 | 3.47 | 0.45 | 3.43×10^-4^ | 0.009 | Lipids and lipid-like molecules | Fatty Acyls | Eicosanoids |
| Taurohyocholic Acid | C26H45NO7S | neg | 7.28 | 496.27 | 3.38 | 1.45 | 3.74×10^-4^ | 0.009 | - | - | - |
| (R)C(R)S-S-Propylcysteine sulfoxide | C6H13NO3S | pos | 0.67 | 180.07 | 3.05 | 1.58 | 0.003 | 0.026 | Organic acids and derivatives | Carboxylic acids and derivatives | Amino acids, peptides, and analogues |
| CAMP | C10H12N5O6P | neg | 0.78 | 328.05 | 3.04 | 1.59 | 1.56×10^-4^ | 0.005 | - | - | - |
| N-lactoyl-Tryptophan | C14H16N2O4 | pos | 3.70 | 241.10 | 2.78 | 1.54 | 2.08×10^-4^ | 0.003 | Organic acids and derivatives | Carboxylic acids and derivatives | Amino acids, peptides, and analogues |
| Hippurate | C9H9NO3 | pos | 1.98 | 180.07 | 2.75 | 1.65 | 1.15×10^-4^ | 0.008 | - | - | - |
| 1-Methylhistidine | C7H11N3O2 | pos | 0.78 | 339.18 | 2.74 | 0.88 | 0.004 | 0.040 | Organic acids and derivatives | Carboxylic acids and derivatives | Amino acids, peptides, and analogues |
| Norketamine | C12H14ClNO | pos | 2.84 | 224.08 | 2.71 | 0.70 | 0.005 | 0.041 | Benzenoids | Benzene and substituted derivatives | Halobenzenes |
| Nicotyrine | C10H10N2 | pos | 3.55 | 159.09 | 2.68 | 0.84 | 0.004 | 0.038 | - | - | - |
| Glutamyltryptophan | C16H19N3O5 | neg | 2.06 | 332.13 | 2.66 | 1.42 | 0.005 | 0.021 | Organic acids and derivatives | Carboxylic acids and derivatives | Amino acids, peptides, and analogues |
| 13,14-Dihydro PGF-1a | C20H38O5 | neg | 6.31 | 357.26 | 2.65 | 0.67 | 0.003 | 0.021 | Lipids and lipid-like molecules | Fatty Acyls | Eicosanoids |
| 1-Methoxy-1H-indole-3-acetonitrile | C11H10N2O | pos | 2.06 | 187.09 | 2.42 | 1.49 | 0.003 | 0.011 | Organoheterocyclic compounds | Indoles and derivatives | Indoles |
| 3-Indolepropionic acid | C10H9NO2 | pos | 1.31 | 176.07 | 2.32 | 1.33 | 0.002 | 0.032 | Organoheterocyclic compounds | Indoles and derivatives | Indolyl carboxylic acids and derivatives |
| N-Formylmethionine | C6H11NO3S | neg | 2.26 | 176.04 | 2.28 | 0.61 | 0.008 | 0.046 | - | - | - |
| Cerasinone | C18H18O6 | neg | 5.72 | 329.10 | 2.25 | 1.47 | 0.009 | 0.049 | Phenylpropanoids and polyketides | Flavonoids | O-methylated flavonoids |
| Soyasaponin II | C47H76O17 | pos | 2.61 | 468.25 | 2.23 | 0.73 | 0.007 | 0.048 | Lipids and lipid-like molecules | Prenol lipids | Terpene glycosides |
| N-Nonanoylglycine | C11H21NO3 | pos | 2.19 | 216.16 | 2.21 | 1.51 | 0.003 | 0.046 | Organic acids and derivatives | Carboxylic acids and derivatives | Amino acids, peptides, and analogues |
| Trans-Grandmarin | C15H16O6 | neg | 5.93 | 291.09 | 2.17 | 1.54 | 0.006 | 0.047 | Phenylpropanoids and polyketides | Coumarins and derivatives | Pyranocoumarins |
| 4-Hydroxy-2-quinolone | C9H7NO2 | pos | 2.64 | 162.06 | 2.15 | 1.07 | 0.007 | 0.048 | - | - | - |
| Geranyl Phosphate | C5H11O4P.[C5H8]n | pos | 4.56 | 199.09 | 2.15 | 0.55 | 0.006 | 0.042 | Lipids and lipid-like molecules | Prenol lipids | Isoprenoid phosphates |
| N-Formylmethionine | C6H11NO3S | neg | 2.26 | 176.04 | 2.15 | 0.91 | 0.019 | 0.156 | - | - | - |
| 8-Hydroxythioguanine | C5H5N5OS | neg | 2.37 | 204.00 | 2.10 | 1.43 | 0.006 | 0.048 | Organoheterocyclic compounds | Imidazopyrimidines | Purines and purine derivatives |
| Tryptophanol | C10H11NO | pos | 2.22 | 144.08 | 2.09 | 1.01 | 0.004 | 0.027 | Organoheterocyclic compounds | Indoles and derivatives | Indoles |
| Malvidin | C17H14O7 | pos | 5.88 | 331.08 | 2.07 | 1.47 | 0.007 | 0.040 | - | - | - |
| 4-Hydroxy-2-quinolone | C9H7NO2 | pos | 2.64 | 162.06 | 2.07 | 1.07 | 0.003 | 0.181 | - | - | - |
| Bilirubin | C33H36N4O6 | pos | 5.87 | 585.27 | 2.06 | 1.08 | 0.046 | 0.410 | Organoheterocyclic compounds | Tetrapyrroles and derivatives | Bilirubins |
| 7-hydroxy-coumarin | C9H6O3 | pos | 1.31 | 204.07 | 2.04 | 1.07 | 0.003 | 0.169 | - | - | - |
| TXB2 | C20H34O6 | pos | 6.03 | 393.23 | 2.03 | 1.05 | 0.001 | 0.103 | - | - | - |
| Sonchifolin | C21H26O6 | neg | 6.76 | 373.17 | 1.96 | 0.75 | 0.005 | 0.034 | Lipids and lipid-like molecules | Prenol lipids | Terpene lactones |

pos = positive ion mode; neg = negative ion mode; M/Z = charge-to-mass ratio; VIP = variable important in projection; FC = fold change; HMDB = human metabolome database; - = the compound classification was unknown.

Table S10 Significantly differential metabolites based on VIP value (top 30) between control and inulin_2 group in serum samples of SCM cows

| **Metabolite** | **Formula** | **Mode** | **Retention time** | **M/Z** | **VIP** | **FC** | ***P*-value** | **Corrected *P*-value** | **HMDB Superclass** | **HMDB Class** | **HMDB Subclass** |
| --- | --- | --- | --- | --- | --- | --- | --- | --- | --- | --- | --- |
| 3-Indolepropionic acid | C11H11NO2 | pos | 5.71 | 190.09 | 3.44 | 1.60 | 2.07×10^-5^ | 0.0004 | Organoheterocyclic compounds | Indoles and derivatives | Indolyl carboxylic acids and derivatives |
| 5,20-DiHETE | C20H32O4 | neg | 6.29 | 381.23 | 3.18 | 0.63 | 1.36×10^-4^ | 0.007 | Lipids and lipid-like molecules | Fatty Acyls | Eicosanoids |
| 7-Ketodeoxycholic acid | C24H38O5 | pos | 6.10 | 389.27 | 3.01 | 1.52 | 0.004 | 0.019 | Lipids and lipid-like molecules | Steroids and steroid derivatives | Bile acids, alcohols and derivatives |
| 13-HpODE | C18H32O4 | neg | 6.19 | 311.22 | 2.86 | 0.66 | 6.85×10^-5^ | 0.0005 | - | - | - |
| Lithocholic acid glycine conjugate | C26H43NO4 | neg | 6.15 | 478.32 | 2.80 | 1.51 | 3.44×10^-4^ | 0.001 | Lipids and lipid-like molecules | Steroids and steroid derivatives | Bile acids, alcohols and derivatives |
| Eicosanoyl-EA | C22H45NO2 | pos | 6.07 | 356.35 | 2.78 | 0.77 | 0.004 | 0.048 |  |  |  |
| 13,14-Dihydro-15-keto-PGE2 | C20H32O5 | neg | 7.71 | 351.22 | 2.77 | 0.65 | 0.002 | 0.032 | Lipids and lipid-like molecules | Fatty Acyls | Eicosanoids |
| 12-HETE | C20H32O3 | neg | 6.33 | 319.23 | 2.75 | 0.71 | 2.12×10^-4^ | 0.005 | - | - | - |
| Taurohyocholic Acid | C26H45NO7S | neg | 7.28 | 496.27 | 2.71 | 1.57 | 3.54×10^-4^ | 0.005 | - | - | - |
| Taurocholic acid | C26H45NO7S | pos | 7.29 | 560.26 | 2.69 | 1.56 | 9.64×10^-4^ | 0.002 | Lipids and lipid-like molecules | Steroids and steroid derivatives | Bile acids, alcohols and derivatives |
| Ciclesonide | C32H44O7 | pos | 8.38 | 579.28 | 2.69 | 1.53 | 0.001 | 0.026 | Lipids and lipid-like molecules | Steroids and steroid derivatives | Pregnane steroids |
| CAMP | C10H12N5O6P | neg | 0.78 | 328.05 | 2.63 | 1.77 | 3.01×10^-4^ | 0.0009 | - | - | - |
| Sulfolithocholylglycine | C26H43NO7S | pos | 6.00 | 496.27 | 2.63 | 1.39 | 0.003 | 0.032 | Lipids and lipid-like molecules | Steroids and steroid derivatives | Bile acids, alcohols and derivatives |
| Vitamin D2 3-glucuronide | C34H52O7 | neg | 6.32 | 617.37 | 2.62 | 1.43 | 0.002 | 0.031 | Lipids and lipid-like molecules | Steroids and steroid derivatives | Vitamin D and derivatives |
| Deoxycholic acid glycine conjugate | C26H43NO5 | neg | 6.10 | 494.31 | 2.61 | 1.43 | 2.21×10^-4^ | 0.004 | Lipids and lipid-like molecules | Steroids and steroid derivatives | Bile acids, alcohols and derivatives |
| Ethyl 1-(methylthio)propyl disulfide | C6H14S3 | neg | 2.94 | 203.00 | 2.52 | 0.72 | 0.007 | 0.032 | Organosulfur compounds | Organic disulfides | Dialkyldisulfides |
| 1-(beta-D-Glucopyranosyloxy)-3-octanone | C14H26O7 | pos | 4.72 | 289.17 | 2.47 | 0.80 | 0.005 | 0.042 | Lipids and lipid-like molecules | Fatty Acyls | Fatty acyl glycosides |
| Nequinate | C22H23NO4 | neg | 6.29 | 346.14 | 2.47 | 1.42 | 0.002 | 0.046 | Organoheterocyclic compounds | Quinolines and derivatives | Quinolones and derivatives |
| Sulfolithocholic acid | C24H40O6S | pos | 3.02 | 474.29 | 2.43 | 1.54 | 0.006 | 0.038 | Lipids and lipid-like molecules | Steroids and steroid derivatives | Bile acids, alcohols and derivatives |
| (1R,2R,4S)-p-Menthane-1,2,8-triol 8-glucoside | C16H30O8 | pos | 2.22 | 368.23 | 2.41 | 0.84 | 0.005 | 0.041 | Organic oxygen compounds | Organooxygen compounds | Carbohydrates and carbohydrate conjugates |
| Glycocholic Acid | C26H43NO6 | neg | 6.12 | 464.30 | 2.40 | 1.51 | 0.003 | 0.021 | Lipids and lipid-like molecules | Steroids and steroid derivatives | Bile acids, alcohols and derivatives |
| Hippuric acid | C9H9NO3 | pos | 2.94 | 180.07 | 2.39 | 1.55 | 5.02×10^-4^ | 0.005 | Benzenoids | Benzene and substituted derivatives | Benzoic acids and derivatives |
| N-Acetyldehydroanonaine | C19H15NO3 | neg | 7.29 | 655.21 | 2.37 | 1.26 | 0.001 | 0.042 | Alkaloids and derivatives | Aporphines | Not Available |
| Persicachrome | C25H36O3 | pos | 6.14 | 426.30 | 2.32 | 1.35 | 0.005 | 0.048 | Lipids and lipid-like molecules | Prenol lipids | Diterpenoids |
| Proline betaine | C7H13NO2 | pos | 0.63 | 287.20 | 2.18 | 1.15 | 0.002 | 0.033 | Organic acids and derivatives | Carboxylic acids and derivatives | Amino acids, peptides, and analogues |
| L-Homocitrulline | C7H15N3O3 | pos | 0.72 | 190.12 | 2.11 | 1.44 | 0.006 | 0.051 | - | - | - |
| 9'-Carboxy-gamma-chromanol | C23H36O4 | neg | 6.16 | 421.26 | 2.06 | 1.07 | 0.002 | 0.017 | Organoheterocyclic compounds | 1-benzopyrans | Benzopyrans |
| O-methoxycatechol-O-sulphate | C7H8O5S | neg | 3.53 | 203.00 | 2.05 | 0.95 | 0.000 | 0.000 | Organic acids and derivatives | Arylsulfates | Organic sulfuric acids and derivatives |
| Geranyl Phosphate | C_5_H_11_O_4_P.[C_5_H_8_] n | pos | 4.56 | 199.09 | 2.04 | 0.91 | 0.000 | 0.016 | Lipids and lipid-like molecules | Isoprenoid phosphates | Prenol lipids |
| Paracetamol sulfate | C8H9NO5S | neg | 2.30 | 230.01 | 2.04 | 0.91 | 0.001 | 0.013 | Organic acids and derivatives | Arylsulfates | Organic sulfuric acids and derivatives |

pos = positive ion mode; neg = negative ion mode; M/Z = charge-to-mass ratio; VIP = variable important in projection; FC = fold change; HMDB = human metabolome database; - = the compound classification was unknown.

Table S11 Significantly differential metabolites based on VIP value (top 30) between control and inulin_3 group in serum samples of SCM cows

| **Metabolite** | **Formula** | **Mode** | **Retention time** | **M/Z** | **VIP** | **FC** | ***P*-value** | **Corrected *P*-value** | **HMDB Superclass** | **HMDB Class** | **HMDB Subclass** |
| --- | --- | --- | --- | --- | --- | --- | --- | --- | --- | --- | --- |
| Indole-3-propionic acid | C11H11NO2 | pos | 4.44 | 172.08 | 4.89 | 1.62 | 3.80×10^-5^ | 0.001 | Organoheterocyclic compounds | Indoles and derivatives | Indolyl carboxylic acids and derivatives |
| N-Acetyltryptophan | C13H14N2O3 | pos | 4.44 | 247.11 | 4.53 | 1.56 | 5.43×10^-5^ | 0.001 | Organic acids and derivatives | Carboxylic acids and derivatives | Amino acids, peptides, and analogues |
| 3-Indolepropionic acid | C11H11NO2 | pos | 5.71 | 190.09 | 4.39 | 1.72 | 0.001 | 0.031 | Organoheterocyclic compounds | Indoles and derivatives | Indolyl carboxylic acids and derivatives |
| 5,20-DiHETE | C20H32O4 | neg | 6.29 | 381.23 | 3.70 | 0.59 | 3.94×10^-5^ | 0.001 | Lipids and lipid-like molecules | Fatty Acyls | Eicosanoids |
| Taurohyocholic Acid | C26H45NO7S | neg | 7.28 | 496.27 | 3.59 | 1.71 | 3.78×10^-5^ | 0.001 | - | - | - |
| CAMP | C10H12N5O6P | neg | 0.78 | 328.05 | 3.56 | 1.80 | 1.53×10^-4^ | 0.005 | - | - | - |
| 8,9-DiHETrE | C20H34O4 | pos | 6.33 | 361.24 | 3.51 | 0.66 | 1.77×10^-4^ | 0.001 | Lipids and lipid-like molecules | Fatty Acyls | Eicosanoids |
| Aminohippuric acid | C9H10N2O3 | pos | 2.13 | 195.08 | 3.45 | 1.11 | 3.67×10^-4^ | 0.004 | Benzenoids | Benzene and substituted derivatives | Benzoic acids and derivatives |
| 12-HETE | C20H32O3 | neg | 6.33 | 319.23 | 3.34 | 0.64 | 1.47×10^-5^ | 0.001 | - | - | - |
| Deoxycholic acid glycine conjugate | C26H43NO5 | neg | 6.10 | 494.31 | 3.34 | 1.63 | 2.45×10^-4^ | 0.006 | Lipids and lipid-like molecules | Steroids and steroid derivatives | Bile acids, alcohols and derivatives |
| 7-Ketodeoxycholic acid | C24H38O5 | pos | 6.10 | 389.27 | 3.13 | 1.56 | 0.001 | 0.008 | Lipids and lipid-like molecules | Steroids and steroid derivatives | Bile acids, alcohols and derivatives |
| 6-Methoxymellein | C11H12O4 | pos | 6.30 | 209.08 | 3.06 | 0.71 | 0.001 | 0.005 | Organoheterocyclic compounds | Benzopyrans | 2-benzopyrans |
| Benzoquinoneacetic acid | C8H6O4 | pos | 6.30 | 149.02 | 3.04 | 0.62 | 0.001 | 0.006 | Organic oxygen compounds | Organooxygen compounds | Carbonyl compounds |
| Sulfolithocholylglycine | C26H43NO7S | pos | 6.00 | 496.27 | 3.03 | 1.56 | 3.98×10^-4^ | 0.003 | Lipids and lipid-like molecules | Steroids and steroid derivatives | Bile acids, alcohols and derivatives |
| Glycocholic Acid | C26H43NO6 | neg | 6.12 | 464.30 | 3.02 | 1.58 | 0.006 | 0.037 | Lipids and lipid-like molecules | Steroids and steroid derivatives | Bile acids, alcohols and derivatives |
| 13-HpODE | C18H32O4 | neg | 6.19 | 311.22 | 2.96 | 0.61 | 4.26×10^-4^ | 0.004 | - | - | - |
| 3-amino-2-naphthoic acid | C11H9NO2 | neg | 5.59 | 186.06 | 2.93 | 1.36 | 0.005 | 0.041 | - | - | - |
| 7-Dehydrocholesterol 5,6-oxide | C27H44O2 | pos | 6.86 | 401.34 | 2.91 | 0.73 | 0.006 | 0.042 | - | - | - |
| Lithocholic acid glycine conjugate | C26H43NO4 | neg | 6.15 | 478.32 | 2.84 | 1.59 | 0.003 | 0.031 | Lipids and lipid-like molecules | Steroids and steroid derivatives | Bile acids, alcohols and derivatives |
| L-Tryptophan | C11H12N2O2 | neg | 2.22 | 203.08 | 2.83 | 1.56 | 0.018 | 0.039 | Organoheterocyclic compounds | Indoles and derivatives | Indolyl carboxylic acids and derivatives |
| L-Homocitrulline | C7H15N3O3 | pos | 0.72 | 190.12 | 2.81 | 1.42 | 0.001 | 0.038 | - | - | - |
| Vitamin D2 3-glucuronide | C34H52O7 | neg | 6.32 | 617.37 | 2.79 | 1.58 | 0.007 | 0.043 | Lipids and lipid-like molecules | Steroids and steroid derivatives | Vitamin D and derivatives |
| 5-Carboxy-2'-deoxyuridine | C10H12N2O7 | pos | 4.22 | 290.10 | 2.79 | 0.62 | 0.006 | 0.042 | Nucleosides, nucleotides, and analogues | Pyrimidine nucleosides | Pyrimidine 2'-deoxyribonucleosides |
| 13,14-Dihydro-15-keto-PGE2 | C20H32O5 | neg | 7.71 | 351.22 | 2.71 | 0.69 | 0.003 | 0.037 | Lipids and lipid-like molecules | Fatty Acyls | Eicosanoids |
| 4,5-DIDEMETHYLSIMMONDSIN | C15H23NO8 | pos | 3.59 | 346.15 | 2.67 | 1.10 | 0.006 | 0.042 | - | - | - |
| Caffeoquinone | C9H6O4 | pos | 6.25 | 179.03 | 2.56 | 1.16 | 0.006 | 0.047 | - | - | - |
| Proline betaine | C7H13NO2 | pos | 0.63 | 287.20 | 2.29 | 1.47 | 0.007 | 0.048 | Organic acids and derivatives | Carboxylic acids and derivatives | Amino acids, peptides, and analogues |
| N2, N2-Dimethylguanosine | C12H17N5O5 | pos | 1.83 | 312.13 | 2.25 | 1.11 | 0.042 | 0.375 | Nucleosides, nucleotides, and analogues | Purine nucleosides | Not Available |
| 6-Hydroxy-R-acenocoumarol | C19H15NO7 | neg | 6.04 | 414.09 | 2.22 | 1.07 | 0.005 | 0.012 | Phenylpropanoids and polyketides | Coumarins and derivatives | Hydroxycoumarins |
| 11b-Hydroxyandrost-4-ene-3,17-dione | C19H26O3 | pos | 6.24 | 344.22 | 2.22 | 0.93 | 0.001 | 0.097 | Organoheterocyclic compounds | Oxazinanes | Morpholines |

pos = positive ion mode; neg = negative ion mode; M/Z = charge-to-mass ratio; VIP = variable important in projection; FC = fold change; HMDB = human metabolome database; - = the compound classification was unknown.

Table S12 Significantly differential metabolites based on VIP value (top 30) between control and inulin_4 group in serum samples of SCM cows

| **Metabolite** | **Formula** | **Mode** | **Retention time** | **M/Z** | **VIP** | **FC** | ***P*-value** | **Corrected *P*-value** | **HMDB Superclass** | **HMDB Class** | **HMDB Subclass** |
| --- | --- | --- | --- | --- | --- | --- | --- | --- | --- | --- | --- |
| Glycocholic Acid | C26H43NO6 | neg | 6.12 | 464.30 | 3.75 | 1.65 | 6.56×10^-5^ | 0.0002 | Lipids and lipid-like molecules | Steroids and steroid derivatives | Bile acids, alcohols and derivatives |
| Taurohyocholic Acid | C26H45NO7S | neg | 7.28 | 496.27 | 3.37 | 1.55 | 9.85×10^-5^ | 0.0004 | - | - | - |
| Lithocholic acid glycine conjugate | C26H43NO4 | neg | 6.15 | 478.32 | 3.12 | 1.70 | 3.18×10^-4^ | 0.001 | Lipids and lipid-like molecules | Steroids and steroid derivatives | Bile acids, alcohols and derivatives |
| 5,20-DiHETE | C20H32O4 | neg | 6.29 | 381.23 | 3.06 | 0.68 | 2.08×10^-4^ | 0.001 | Lipids and lipid-like molecules | Fatty Acyls | Eicosanoids |
| 12-HETE | C20H32O3 | neg | 6.33 | 319.23 | 3.03 | 0.69 | 3.35×10^-4^ | 0.003 | - | - | - |
| 8,9-DiHETrE | C20H34O4 | pos | 6.33 | 361.24 | 3.01 | 0.71 | 2.05×10^-4^ | 0.002 | Lipids and lipid-like molecules | Fatty Acyls | Eicosanoids |
| Deoxycholic acid glycine conjugate | C26H43NO5 | neg | 6.10 | 494.31 | 2.87 | 1.52 | 1.76×10^-4^ | 0.003 | Lipids and lipid-like molecules | Steroids and steroid derivatives | Bile acids, alcohols and derivatives |
| CAMP | C10H12N5O6P | neg | 0.78 | 328.05 | 2.87 | 1.78 | 2.08×10^-4^ | 0.001 | - | - | - |
| 7-Ketodeoxycholic acid | C24H38O5 | pos | 6.10 | 389.27 | 2.83 | 1.38 | 2.73×10^-4^ | 0.002 | Lipids and lipid-like molecules | Steroids and steroid derivatives | Bile acids, alcohols and derivatives |
| 3-keto Fusidic acid | C31H46O7 | neg | 6.23 | 511.30 | 2.77 | 1.31 | 0.009 | 0.043 | Lipids and lipid-like molecules | Steroids and steroid derivatives | Oxosteroids |
| Sulfolithocholylglycine | C26H43NO7S | pos | 6.00 | 496.27 | 2.74 | 1.40 | 0.002 | 0.020 | Lipids and lipid-like molecules | Steroids and steroid derivatives | Bile acids, alcohols and derivatives |
| Monoisobutyl phthalate | C12H14O4 | pos | 6.31 | 205.09 | 2.73 | 0.85 | 0.006 | 0.031 | - | - | - |
| Ethyl 1-(methylthio)propyl disulfide | C6H14S3 | neg | 2.94 | 203.00 | 2.71 | 0.67 | 4.16×10^-4^ | 0.002 | Organosulfur compounds | Organic disulfides | Dialkyldisulfides |
| Taurocholic acid | C26H45NO7S | pos | 7.29 | 560.26 | 2.68 | 1.52 | 1.15×10^-4^ | 0.006 | Lipids and lipid-like molecules | Steroids and steroid derivatives | Bile acids, alcohols and derivatives |
| 13,14-Dihydro PGF-1a | C20H38O5 | neg | 6.31 | 357.26 | 2.65 | 0.65 | 0.005 | 0.041 | Lipids and lipid-like molecules | Fatty Acyls | Eicosanoids |
| Hippuric acid | C9H9NO3 | pos | 2.94 | 180.07 | 2.64 | 1.62 | 0.001 | 0.013 | Benzenoids | Benzene and substituted derivatives | Benzoic acids and derivatives |
| Docosatrienoic acid | C22H38O2 | pos | 6.33 | 357.28 | 2.54 | 1.34 | 0.004 | 0.034 | Lipids and lipid-like molecules | Fatty Acyls | Fatty acids and conjugates |
| Ciclesonide | C32H44O7 | pos | 8.38 | 579.28 | 2.52 | 1.42 | 0.006 | 0.048 | Lipids and lipid-like molecules | Steroids and steroid derivatives | Pregnane steroids |
| 6-Methoxymellein | C11H12O4 | pos | 6.30 | 209.08 | 2.51 | 0.89 | 0.002 | 0.044 | Organoheterocyclic compounds | Benzopyrans | 2-benzopyrans |
| Desmosine | C24H40N5O8+ | neg | 6.00 | 547.26 | 2.48 | 1.13 | 0.005 | 0.044 | Organic acids and derivatives | Carboxylic acids and derivatives | Tetracarboxylic acids and derivatives |
| L-Tryptophan | C11H12N2O2 | neg | 2.22 | 203.08 | 2.47 | 1.50 | 0.002 | 0.029 | Organoheterocyclic compounds | Indoles and derivatives | Indolyl carboxylic acids and derivatives |
| Vitamin D2 3-glucuronide | C34H52O7 | neg | 6.32 | 617.37 | 2.46 | 1.44 | 0.004 | 0.036 | Lipids and lipid-like molecules | Steroids and steroid derivatives | Vitamin D and derivatives |
| 3-Indolepropionic acid | C11H11NO2 | pos | 5.71 | 190.09 | 2.46 | 1.47 | 0.004 | 0.014 | Organoheterocyclic compounds | Indoles and derivatives | Indolyl carboxylic acids and derivatives |
| Persicachrome | C25H36O3 | pos | 6.14 | 426.30 | 2.44 | 1.17 | 0.005 | 0.039 | Lipids and lipid-like molecules | Prenol lipids | Diterpenoids |
| Benzoquinoneacetic acid | C8H6O4 | pos | 6.30 | 149.02 | 2.36 | 0.90 | 0.002 | 0.045 | Organic oxygen compounds | Organooxygen compounds | Carbonyl compounds |
| Xanthine | C5H4N4O2 | neg | 0.89 | 151.03 | 2.12 | 1.23 | 0.005 | 0.040 | Organoheterocyclic compounds | Imidazopyrimidines | Purines and purine derivatives |
| O-methoxycatechol-O-sulphate | C7H8O5S | neg | 3.53 | 203.00 | 2.03 | 0.94 | 9.74×10^-8^ | 3.65×10^-5^ | Organic acids and derivatives | Organic sulfuric acids and derivatives | Arylsulfates |
| 3-Indoleacetic Acid | C10H9NO2 | pos | 1.31 | 176.07 | 2.01 | 1.10 | 0.001 | 0.012 | - | - | - |
| P-Cresol glucuronide | C13H16O7 | neg | 3.73 | 283.08 | 2.00 | 0.93 | 0.001 | 0.005 | Organic oxygen compounds | Organooxygen compounds | Carbohydrates and carbohydrate conjugates |
| Antibiotic SB 202742 | C24H34O3 | pos | 6.19 | 353.25 | 1.98 | 1.08 | 0.001 | 0.012 | Benzenoids | Benzene and substituted derivatives | Benzoic acids and derivatives |

pos = positive ion mode; neg = negative ion mode; M/Z = charge-to-mass ratio; VIP = variable important in projection; FC = fold change; HMDB = human metabolome database; - = the compound classification was unknown.

Table S13 Differential metabolites (average relative abundance) in the feces and serum among control and 4 inulin treatment groups

| **Metabolite** | **Groups (n = 8)** | | | | | **SEM** | ***P-*value** | **Corrected *P*-value** |
| --- | --- | --- | --- | --- | --- | --- | --- | --- |
|  | **Con** | **Inu_1** | **Inu_2** | **Inu_3** | **Inu_4** |  |  |  |
| **Feces** | | | | | | | | |
| Deoxycholic acid | 5.32^c^ | 7.85^b^ | 8.08^b^ | 8.80^a^ | 8.84^a^ | 0.281 | 0.0005 | 0.006 |
| Taurodeoxycholic acid | 5.24^c^ | 7.05^b^ | 7.19^b^ | 8.44^a^ | 8.43^a^ | 0.289 | 0.0093 | 0.040 |
| Melibiose | 4.34^c^ | 6.73^b^ | 7.86^a^ | 7.71^a^ | 7.08^ab^ | 0.285 | 0.0002 | 0.003 |
| Withanolide | 5.04^b^ | 7.51^ab^ | 7.95^a^ | 8.03^a^ | 7.48^ab^ | 0.247 | 0.0101 | 0.045 |
| Tetrahydroneopterin | 5.01^c^ | 6.75^b^ | 7.59^a^ | 7.99^a^ | 7.56^a^ | 0.238 | 0.0114 | 0.047 |
| L-Glutamate | 4.83^c^ | 6.29^b^ | 7.74^a^ | 7.62^a^ | 7.01^ab^ | 0.239 | 0.0097 | 0.046 |
| Coprocholic acid | 5.01^c^ | 6.97^b^ | 7.16^b^ | 7.78^a^ | 7.91^a^ | 0.233 | 0.0005 | 0.032 |
| 13(S)-HpODE | 6.51^a^ | 5.41^b^ | 4.28^c^ | 4.01^c^ | 4.30^c^ | 0.210 | 0.0030 | 0.020 |
| 12-oxo-20-trihydroxy-leukotriene B4 | 5.94^a^ | 4.11^b^ | 3.72^bc^ | 3.06^c^ | 3.88^bc^ | 0.218 | 0.0109 | 0.046 |
| Cer(d18:0/13:0) | 6.79^a^ | 5.13^b^ | 4.35^c^ | 4.12^c^ | 4.81^bc^ | 0.219 | 0.0010 | 0.010 |
| 12,13- diHOME | 5.92^a^ | 4.14^b^ | 3.85^bc^ | 3.16^c^ | 3.72^bc^ | 0.196 | 0.0008 | 0.009 |
| Serum | | | | | | | | |
| CAMP | 3.18^b^ | 5.06^a^ | 5.62^a^ | 5.71^a^ | 5.64^a^ | 0.215 | 0.0089 | 0.041 |
| Taurohyocholic acid | 4.17^c^ | 6.04^b^ | 6.55^b^ | 7.12^a^ | 6.47^b^ | 0.226 | 0.0027 | 0.012 |
| 3-Indolepropionic acid | 4.03^c^ | 5.36^b^ | 6.44^a^ | 6.94^a^ | 5.91^b^ | 0.224 | 0.0046 | 0.027 |
| Hippuric acid | 4.63^c^ | 5.74^b^ | 7.19^a^ | 6.10^b^ | 7.48^a^ | 0.230 | 0.0031 | 0.021 |
| Deoxycholic acid glycine conjugate | 4.12^c^ | 4.72^c^ | 5.88^b^ | 6.71^a^ | 6.26^a^ | 0.217 | 0.0043 | 0.026 |
| Lithocholic acid glycine conjugate | 4.28^d^ | 5.22^c^ | 6.46^b^ | 6.82^b^ | 7.27^a^ | 0.246 | 0.0035 | 0.017 |
| Vitamin D2 3-glucuronide | 4.23^c^ | 5.01^b^ | 6.06^ab^ | 6.69^a^ | 6.07^ab^ | 0.196 | 0.0092 | 0.041 |
| 7-ketodeoxycholic acid | 4.07^c^ | 5.06^b^ | 6.17^a^ | 6.34^a^ | 5.61^b^ | 0.184 | 0.0076 | 0.032 |
| glycocholic acid | 5.14^b^ | 5.77^b^ | 7.75^ab^ | 8.13^a^ | 8.51^a^ | 0.301 | 0.0093 | 0.042 |
| Sulfolithocholylglycine | 4.41^c^ | 5.04^b^ | 6.14^ab^ | 6.89^a^ | 6.18^ab^ | 0.199 | 0.010 | 0.047 |
| Taurocholic acid | 4.50^c^ | 5.46^b^ | 7.04^ab^ | 7.42^a^ | 6.82^ab^ | 0.245 | 0.0077 | 0.030 |
| L-Tryptophan | 4.34^c^ | 4.85^c^ | 5.41^b^ | 6.76^a^ | 6.52^a^ | 0.209 | 0.011 | 0.045 |
| 13-HpODE | 6.27^a^ | 5.79^b^ | 4.16^c^ | 3.85^c^ | 5.29^b^ | 0.208 | 0.0089 | 0.038 |
| 12-HETE | 7.07^a^ | 4.24^c^ | 5.04^b^ | 4.51^c^ | 4.87^b^ | 0.224 | 0.014 | 0.044 |
| 5,20-DiHETE | 6.78^a^ | 5.49^b^ | 4.30^c^ | 4.00^c^ | 4.58^b^ | 0.225 | 0.0076 | 0.033 |
| 8,9-DiHETrE | 6.53^a^ | 5.26^b^ | 5.24^b^ | 4.30^c^ | 4.63^c^ | 0.171 | 0.0083 | 0.038 |
| 13,14-Dihydro-15-keto-PGE2 | 6.18^a^ | 6.31^a^ | 4.04^c^ | 4.28^c^ | 5.27^b^ | 0.209 | 0.0092 | 0.037 |
| 13,14-Dihydro PGF-1α | 6.42^a^ | 4.30^c^ | 5.17^b^ | 4.94^b^ | 4.18^c^ | 0.180 | 0.0096 | 0.035 |

13(S)-HpODE = 13-L-hydroperoxylinoleic acid; Cer = ceramide; 12, 13-DiHODE = 12, 13-hydroxyoctadecadienoate; CAMP = cyclic adenosine monophosphate; 12-HETE = 12(S)-Hydroxyeicosatetraenoic acid; 8,9-DiHETrE = 8,9-Dihydroxyeicosatrienoic acid; 5, 20-DiHETE = 5, 20-Dihydroxyeicosatetraenoate; Con = control group; Inu_1 = inulin_1 group, the inulin addition level was 100 g/d per cow; Inu_2 = inulin_2 group, the inulin addition level was 200 g/d per cow; Inu_3 = inulin_3 group, the inulin addition level was 300 g/d per cow; Inu_4 = inulin_4 group, the inulin addition level was 400 g/d per cow; ^a, b, c^ = within a row, different letters differed significantly (Corrected *P* < 0.05).

Table S14 Up-regulated proteins in serum of SCM dairy cow after inulin treatment

| **Accession ID** | **Protein name** | **Gene name** | **GO Term level 2** | **GO term level 3** | **FC** | | | | ***P*-value** | **Corrected *P-*value** |
| --- | --- | --- | --- | --- | --- | --- | --- | --- | --- | --- |
|  |  |  |  |  | **Inu_1/Con** | **Inu_2/Con** | **Inu_3/Con** | **Inu_4/Con** |  |  |
| A0A452DHX8 | Amine oxidase | LOC100138645 | catalytic activity | oxidoreductase activity, acting on the CH-NH2 group of donors | 1.33 | 3.17 | 3.07 | 2.16 | 5.20×10^-5^ | 0.010 |
| A0A3Q1N2P4 | 4-hydroxyphenylpyruvate dioxygenase | HPD | catalytic activity | dioxygenase activity | 1.26 | 1.79 | 2.30 | 1.62 | 8.90×10^-5^ | 0.011 |
| A0A140T8A5 | Isocitrate dehydrogenase [NADP] | IDH1 | binding; catalytic activity; metabolic process; response to stimulus | nucleoside phosphate binding; oxidoreductase activity, acting on CH-OH group of donors; tricarboxylic acid cycle; response to oxidative stress | 1.24 | 2.13 | 2.42 | 1.82 | 3.00×10^-4^ | 0.017 |
| F1MQT9 | CD44 antigen | CD44 | molecular transducer activity; protein-containing complex; immune system process | immune receptor activity; macrophage migration inhibitory factor receptor complex; lymphocyte activation | 1.22 | 1.83 | 3.09 | 2.00 | 5.79×10^-4^ | 0.020 |
| F1N3Q7 | Apolipoprotein A-IV | APOA4 | transporter activity; binding; protein-containing complex; immune system process; multicellular organismal process; response to stimulus | lipid transfer activity; sterol transporter activity; phospholipid binding; lipoprotein particle; innate immune response; plasma lipoprotein particle remodeling; innate immune response | 1.30 | 2.74 | 3.02 | 2.18 | 0.001 | 0.035 |
| P80109 | Phosphatidylinositol-glycan-specific phospholipase D | GPLD1 | catalytic activity; protein-containing complex; immune system process; metabolic process; localization | hydrolase activity, acting on ester bonds; lipoprotein particle; immune response-activating signal transduction; lipid metabolic process; transport | 1.26 | 3.08 | 3.24 | 2.41 | 0.003 | 0.042 |
| F6R4N7 | Superoxide dismutase [Cu-Zn] | SOD3 | catalytic activity; cellular process; response to stimulus | oxidoreductase activity, acting on superoxide radicals as acceptor; cellular oxidant detoxification; response to oxygen levels | 1.23 | 2.88 | 3.43 | 2.40 | 0.002 | 0.047 |
| Q3ZBD7 | Glucose-6-phosphate isomerase | GPI | binding; catalytic activity; immune system process; metabolic process | signaling receptor binding; monosaccharide binding; intramolecular oxidoreductase activity; erythrocyte homeostasis; ATP generation from ADP | 1.37 | 1.76 | 2.11 | 1.51 | 0.002 | 0.048 |
| P81644 | Apolipoprotein A-II | APOA2 | transporter activity; binding; protein-containing complex; multicellular organismal process; response to stimulus | lipid transfer activity; sterol transporter activity; protein-lipid complex binding; lipoprotein particle; high-density lipoprotein particle clearance; defense response | 1.21 | 2.35 | 2.62 | 1.97 | 0.002 | 0.049 |
| A0A3Q1LSR2 | Coagulation factor XIII B chain | F13B | multicellular organismal process; biological regulation | blood coagulation; regulation of cellular process | 1.39 | 1.40 | 1.27 | 1.55 | 0.003 | 0.058 |
| E1BH06 | C4a anaphylatoxin | C4A | molecular function regulator; immune system process; biological regulation | peptidase regulator activity; complement activation; complement activation; regulation of immune system process | 1.29 | 2.17 | 2.39 | 1.79 | 0.004 | 0.063 |
| A0A3Q1MNN6 | Uncharacterized protein | LOC506828 | molecular function regulator | peptidase regulator activity; enzyme inhibitor activity | 1.31 | 1.50 | 1.41 | 1.36 | 0.008 | 0.069 |
| P37141 | Glutathione peroxidase 3 | GPX3 | antioxidant activity; catalytic activity; response to stimulus | glutathione peroxidase activity; oxidoreductase activity, acting on peroxide as acceptor; response to oxidative stress | 1.25 | 1.61 | 1.74 | 1.46 | 0.008 | 0.069 |
| A0A3Q1NEQ0 | Uncharacterized protein | LOC525947 | molecular function regulator | enzyme inhibitor activity | 1.32 | 1.30 | 1.25 | 1.54 | 0.008 | 0.070 |
| A0A452DHZ7 | Adipocyte plasma membrane-associated protein | APMAP | catalytic activity; cellular anatomical entity | carbon-nitrogen lyase activity; integral component of membrane | 1.22 | 1.23 | 1.67 | 1.57 | 0.008 | 0.071 |
| A0A3Q1LLI6 | Uncharacterized protein | LOC506828 | molecular function regulator | peptidase regulator activity; enzyme inhibitor activity | 1.50 | 1.29 | 0.96 | 1.30 | 0.008 | 0.071 |
| A0A452DJ62 | Thrombospondin-4 | THBS4 | molecular function regulator; binding; biological regulation | signaling receptor activator activity; integrin binding; regulation of immune system process | 1.32 | 1.27 | 1.81 | 1.42 | 0.009 | 0.072 |
| A0A3Q1M8I5 | Uncharacterized protein | LOC506828 | molecular function regulator; | peptidase regulator activity; enzyme inhibitor activity | 1.55 | 1.26 | 0.98 | 1.58 | 0.009 | 0.072 |
| A5PKH3 | Fumarylacetoacetase | FAH | binding; catalytic activity | cation binding; hydrolase activity, acting on acid carbon-carbon bonds | 1.62 | 2.36 | 2.12 | 2.22 | 0.013 | 0.085 |
| E1BHY6 | Granulin precursor | GRN | / | / | 1.31 | 1.13 | 1.82 | 1.38 | 0.013 | 0.085 |
| E1BLA8 | Golgi membrane protein 1 | GOLM1 | cellular anatomical entity; cellular anatomical entity | integral component of membrane; intracellular organelle | 1.23 | 1.33 | 1.39 | 1.39 | 0.013 | 0.085 |
| A0A3Q1MSL5 | Fumarate hydratase, mitochondrial | FH | catalytic activity; cellular anatomical entity; metabolic process | carbon-oxygen lyase activity; intracellular organelle; tricarboxylic acid cycle | 1.27 | 1.48 | 1.64 | 1.50 | 0.012 | 0.086 |
| F1MJH1 | Actin-depolymerizing factor | GSN | binding; cellular process | cytoskeletal protein binding; cellular component organization | 1.26 | 1.27 | 1.52 | 1.48 | 0.012 | 0.087 |
| F1MW44 | Coagulation factor XIII A chain | F13A1 | catalytic activity; metabolic process | transferase activity; protein metabolic process | 1.27 | 1.26 | 1.70 | 1.46 | 0.015 | 0.095 |
| A0A3Q1LPF0 | Apolipoprotein E | APOE | protein-containing complex; metabolic process; multicellular organismal process | lipid metabolic process; plasma lipoprotein particle assembly; very-low-density lipoprotein particle clearance; triglyceride-rich lipoprotein particle clearance | 1.16 | 1.49 | 1.24 | 0.96 | 0.015 | 0.095 |
| F1MZ96 | Uncharacterized protein | / | / | / | 1.23 | 1.35 | 1.37 | 1.30 | 0.016 | 0.095 |
| F1N2K1 | Prenylcysteine oxidase 1 | PCYOX1 | catalytic activity; protein-containing complex | oxidoreductase activity, acting on a sulfur group of donors; lipoprotein particle | 1.21 | 1.29 | 1.35 | 1.23 | 0.015 | 0.096 |
| F1N5M2 | Gc-globulin | GC | binding; metabolic process | cytoskeletal protein binding; lipid metabolic process | 1.24 | 1.29 | 1.21 | 1.38 | 0.017 | 0.097 |
| F2X2F3 | Insulin-like growth factor I | IGF1 | protein-containing complex; biological regulation | insulin-like growth factor binding protein complex; regulation of immune system process | 1.25 | 1.26 | 1.22 | 1.29 | 0.018 | 0.101 |
| G1K122 | Retinol-binding protein | RBP4 | transporter activity; binding; localization | retinol transmembrane transporter activity; isoprenoid binding; transport | 1.36 | 1.23 | 1.30 | 1.39 | 0.019 | 0.104 |
| G3MXL6 | Fructose-bisphosphate aldolase | ALDOB | binding; metabolic process | enzyme binding; generation of precursor metabolites and energy; ATP generation from ADP | 1.21 | 1.28 | 1.29 | 1.36 | 0.021 | 0.112 |
| G3MYZ3 | Afamin | AFM | binding; biological regulation | fatty acid binding; regulation of protein stability | 1.27 | 1.25 | 1.26 | 1.47 | 0.025 | 0.123 |
| G3N0J2 | WAP four-disulfide core domain protein 18 | WFDC18 | molecular function regulator | peptidase regulator activity; enzyme inhibitor activity | 1.21 | 1.27 | 1.26 | 1.22 | 0.026 | 0.126 |
| G3X6N3 | Beta-1 metal-binding globulin | TF | binding; biological regulation; interspecies interaction between organisms | signaling receptor binding; regulation of catalytic activity; response to bacterium | 1.26 | 1.40 | 1.46 | 1.25 | 0.030 | 0.136 |
| O46375 | Transthyretin | TTR | molecular function regulator; localization | signaling receptor activator activity; transport | 1.27 | 1.37 | 1.24 | 1.22 | 0.032 | 0.140 |
| P12763 | Alpha-2-HS-glycoprotein | AHSG | molecular function regulator; biological regulation; response to stimulus | peptidase regulator activity; regulation of catalytic activity; defense response | 1.25 | 1.20 | 1.39 | 1.60 | 0.035 | 0.152 |
| P17690 | Beta-2-glycoprotein 1 | APOH | binding | glycosaminoglycan binding | 1.31 | 1.22 | 1.25 | 1.22 | 0.038 | 0.157 |
| P23805 | Conglutinin | CGN1 | binding; cellular anatomical entity | lipopolysaccharide binding; membrane-bounded organelle | 1.26 | 1.40 | 1.67 | 1.77 | 0.037 | 0.158 |
| P35445 | Cartilage oligomeric matrix protein | COMP | binding; biological regulation; localization | enzyme binding; cytokine binding; regulation of hemostasis; transport | 1.26 | 1.50 | 1.80 | 1.34 | 0.042 | 0.165 |
| P60712 | Actin, cytoplasmic 1 | ACTB | structural molecule activity; binding; protein-containing complex | structural constituent of postsynapse; enzyme binding; transferase complex | 1.29 | 1.42 | 1.44 | 1.43 | 0.041 | 0.165 |
| Q09TE3 | Insulin-like growth factor binding protein acid labile subunit | IGFALS | protein-containing complex | insulin-like growth factor binding protein complex | 1.31 | 1.27 | 1.38 | 1.22 | 0.044 | 0.169 |
| Q17QC8 | Complement factor properdin | CFP | / | / | 1.25 | 1.36 | 1.70 | 1.50 | 0.047 | 0.169 |
| Q2KIH2 | ApoN protein | APON | / | / | 1.20 | 1.29 | 1.37 | 1.31 | 0.045 | 0.170 |
| Q2KIW1 | Paraoxonase 1 | PON1 | binding; catalytic activity; protein-containing complex; cellular process | phospholipid binding; hydrolase activity, acting on ester bonds; lipoprotein particle; cellular lipid metabolic process | 1.64 | 1.40 | 1.26 | 1.18 | 0.046 | 0.170 |
| A0A3Q1LVB8 | Biogenesis of lysosome-related organelles complex 1 subunit 1 | / | protein-containing complex | BLOC-1 complex | 1.23 | 1.41 | 1.26 | 1.21 | 0.046 | 0.170 |
| Q3SZJ0 | Argininosuccinate lyase | ASL | catalytic activity | carbon-nitrogen lyase activity | 1.29 | 1.75 | 1.39 | 1.44 | 0.049 | 0.171 |
| Q3Y5Z3 | Adiponectin | ADIPOQ | molecular function regulator | signaling receptor activator activity | 1.26 | 1.16 | 1.41 | 1.32 | 0.046 | 0.171 |
| Q6B855 | Transketolase | TKT | binding; catalytic activity | identical protein binding; transferase activity | 1.31 | 1.40 | 1.29 | 1.44 | 0.049 | 0.174 |

GO = gene ontology, FC = fold change; Con = control group; Inu_1 = inulin_1 group, the inulin addition level was 100 g/d per cow; Inu_2 = inulin_2 group, the inulin addition level was 200 g/d per cow; Inu_3 = inulin_3 group, the inulin addition level was 300 g/d per cow; Inu_4 = inulin_4 group, the inulin addition level was 400 g/d per cow.

Table S15 Down-regulated proteins in serum of SCM dairy cow after inulin treatment

| **Accession ID** | **Protein name** | **Gene name** | **GO Term level 2** | **GO term level 3** | **FC** | | | | ***P*-value** | **Corrected *P-*value** |
| --- | --- | --- | --- | --- | --- | --- | --- | --- | --- | --- |
|  |  |  |  |  | **Inu_1/Con** | **Inu_2/Con** | **Inu_3/Con** | **Inu_4/Con** |  |  |
| A0A3Q1LRG2 | Cathelicidin-1 | CATHL1 | binding; immune system process; interspecies interaction between organisms; response to stimulus | lipopolysaccharide binding; innate immune response; humoral immune response; response to bacterium; defense response | 0.59 | 0.14 | 0.11 | 0.12 | 0.001 | 0.020 |
| G3N1U4 | Serpin A3-3 | SERPINA3-3 | molecular function regulator; biological regulation; | peptidase regulator activity; enzyme inhibitor activity; regulation of catalytic activity | 0.61 | 0.39 | 0.30 | 0.70 | 0.001 | 0.032 |
| Q3ZCJ8 | Dipeptidyl peptidase 1 | CTSC | immune system process; biological regulation; cellular process; response to stimulus | leukocyte mediated immunity; regulation of immune system process; leukocyte mediated cytotoxicity; adaptive immune response | 0.81 | 0.73 | 0.53 | 0.64 | 0.002 | 0.037 |
| G8JKW7 | Serpin A3-7 | SERPINA3-7 | molecular function regulator; biological regulation | peptidase regulator activity; enzyme inhibitor activity; regulation of catalytic activity | 0.64 | 0.40 | 0.33 | 0.63 | 0.002 | 0.040 |
| Q28017 | Platelet-activating factor acetylhydrolase | PLA2G7 | binding; catalytic activity; protein-containing complex; biological regulation; metabolic process; multicellular organismal process | phospholipid binding; hydrolase activity, acting on ester bonds; lipoprotein particle; regulation of immune system process; low-density lipoprotein particle remodeling | 0.748 | 0.53 | 0.52 | 0.57 | 0.002 | 0.042 |
| A2I7N1 | Serpin A3-5 | SERPINA3-5 | molecular function regulator; biological regulation | peptidase regulator activity; enzyme inhibitor activity; regulation of catalytic activity | 0.78 | 0.52 | 0.52 | 0.73 | 0.002 | 0.043 |
| Q29RY7 | Fibroleukin | FGL2 | molecular function regulator; immune system process; biological regulation | peptidase regulator activity; leukocyte activation involved in immune response; immunoglobulin production; regulation of immune system process | 0.73 | 0.75 | 0.59 | 0.64 | 0.002 | 0.043 |
| Q2TBI0 | Lipopolysaccharide-binding protein | LBP | binding; immune system process | lipopolysaccharide binding; lipoteichoic acid binding; cell activation involved in immune response; leukocyte activation involved in immune response; leukocyte chemotaxis; leukocyte migration involved in inflammatory response | 0.73 | 0.81 | 0.71 | 0.70 | 0.003 | 0.045 |
| Q2TBU0 | Haptoglobin | HP | binding; catalytic activity; interspecies interaction between organisms; response to stimulus | hemoglobin binding; peptidase activity; response to bacterium; response to external biotic stimulus | 0.73 | 0.60 | 0.58 | 0.64 | 0.003 | 0.047 |
| A0A3Q1MA31 | Inter-alpha-trypsin inhibitor heavy chain H4 | ITIH4 | molecular function regulator; response to stimulus | peptidase regulator activity; enzyme inhibitor activity; defense response; | 0.80 | 0.67 | 0.65 | 0.72 | 0.004 | 0.049 |
| F1N6H1 | LDL receptor related protein 2 | LRP2 | binding; localization | chaperone binding; transport | 0.75 | 0.64 | 0.59 | 0.73 | 0.004 | 0.056 |
| F1MM86 | Complement component C6 | C6 | protein-containing complex; immune system process; biological regulation; response to stimulus | plasma membrane protein complex; complement activation; response to external biotic stimulus; defense response | 0.89 | 0.68 | 0.76 | 0.78 | 0.004 | 0.059 |
| A4IFA5 | VASN protein | VASN | binding; cellular anatomical entity; biological regulation; response to stimulus | cytokine binding; integral component of membrane; plasma membrane; regulation of response to stimulus; response to hypoxia | 0.72 | 0.89 | 0.76 | 0.68 | 0.006 | 0.061 |
| Q2HJ87 | Glucocorticoid modulatory element-binding protein 1 | GMEB1 | transcription regulator activity; binding | transcription coactivator activity; nucleic acid binding | 0.70 | 0.82 | 0.63 | 0.73 | 0.006 | 0.061 |
| A0A3Q1M5Q6 | Extracellular matrix protein 1 | ECM1 | binding; biological regulation; response to stimulus | laminin binding; enzyme binding; regulation of catalytic activity; regulation of immune system process; defense response | 0.65 | 0.74 | 0.74 | 0.25 | 0.007 | 0.066 |
| E1BFB4 | Serine/threonine-protein kinase mTOR | MTOR | binding; catalytic activity; protein-containing complex | nucleoside phosphate binding; phosphoprotein binding; protein kinase activity; TORC2 complex | 0.82 | 0.87 | 0.56 | 0.77 | 0.008 | 0.069 |
| A0A3Q1ML26 | Ig-like domain-containing protein | IGH | / | / | 0.74 | 0.84 | 0.67 | 0.55 | 0.011 | 0.083 |
| P01045 | Kininogen-2 | KNG2 | molecular function regulator; binding; biological regulation | peptidase regulator activity; signaling receptor binding; regulation of response to stimulus | 0.19 | 0.66 | 0.64 | 0.67 | 0.013 | 0.085 |
| A0A3Q1LJT1 | Ig-like domain-containing protein | IGLL1 | / | / | 0.76 | 0.71 | 0.60 | 0.74 | 0.012 | 0.088 |
| Q5GN72 | Alpha-1-acid glycoprotein | agp | biological regulation; response to stimulus | regulation of immune system process; defense response | 0.90 | 0.84 | 0.78 | 0.65 | 0.013 | 0.092 |
| A0A3Q1NJB1 | Ceruloplasmin | CP | catalytic activity; localization | oxidoreductase activity; transport | 0.56 | 0.79 | 0.85 | 0.82 | 0.015 | 0.097 |
| A5PJ69 | SERPINA10 protein | SERPINA10 | molecular function regulator; biological regulation; | peptidase regulator activity; enzyme inhibitor activity; regulation of catalytic activity | 0.86 | 0.81 | 0.72 | 0.80 | 0.020 | 0.107 |
| G3N1H5 | Ig-like domain-containing protein | LOC100300716 | / | / | 0.80 | 0.74 | 0.80 | 0.70 | 0.021 | 0.111 |
| P28800 | Alpha-2-antiplasmin | SERPINF2 | molecular function regulator; binding; biological regulation | peptidase regulator activity; identical protein binding; regulation of catalytic activity | 0.76 | 0.66 | 0.79 | 0.61 | 0.021 | 0.111 |
| G5E604 | Ig-like domain-containing protein | IGLL | / | / | 0.78 | 0.75 | 0.72 | 0.74 | 0.026 | 0.124 |
| A6QPQ2 | Serpin A3-8 | SERPINA3-8 | molecular function regulator; biological regulation; | peptidase regulator activity; enzyme inhibitor activity; regulation of catalytic activity | 0.71 | 0.65 | 0.65 | 0.66 | 0.027 | 0.126 |
| Q3T052 | Inter-alpha-trypsin inhibitor heavy chain H4 | ITIH4 | molecular function regulator; response to stimulus | peptidase regulator activity; enzyme inhibitor activity; defense response | 0.61 | 0.88 | 0.72 | 0.78 | 0.029 | 0.132 |
| Q3ZCL0 | Cysteine-rich secretory protein 2 | CRISP3 | cellular anatomical entity | intracellular organelle | 0.72 | 0.67 | 0.61 | 0.68 | 0.038 | 0.157 |
| A0A3Q1MTR1 | Ciliary rootlet coiled-coil, rootletin | CROCC | binding; cellular anatomical entity | cytoskeletal protein binding; intracellular organelle | 0.61 | 0.85 | 0.81 | 0.79 | 0.040 | 0.163 |
| A0A3Q1LVM5 | Inter-alpha-trypsin inhibitor heavy chain H1 | ITIH1 | molecular function regulator | peptidase regulator activity | 0.60 | 0.68 | 0.65 | 0.82 | 0.045 | 0.170 |

GO = gene ontology, FC = fold change; Con = control group; Inu_1 = inulin_1 group, the inulin addition level was 100 g/d per cow; Inu_2 = inulin_2 group, the inulin addition level was 200 g/d per cow; Inu_3 = inulin_3 group, the inulin addition level was 300 g/d per cow; Inu_4 = inulin_4 group, the inulin addition level was 400 g/d per cow.
