# Supplementary material for: Changes in the Profile of Fecal Microbiota and Metabolites as Well as Serum Metabolites and Proteome After Dietary Inulin Supplementation in Dairy Cows With Subclinical Mastitis
Source: Front Microbiol. 2022 Apr 4;13:809139. doi: 10.3389/fmicb.2022.809139 (PMC9037088; doi:10.3389/fmicb.2022.809139)
Supplement: Supplementary file 2 [file Data_Sheet_2.docx]

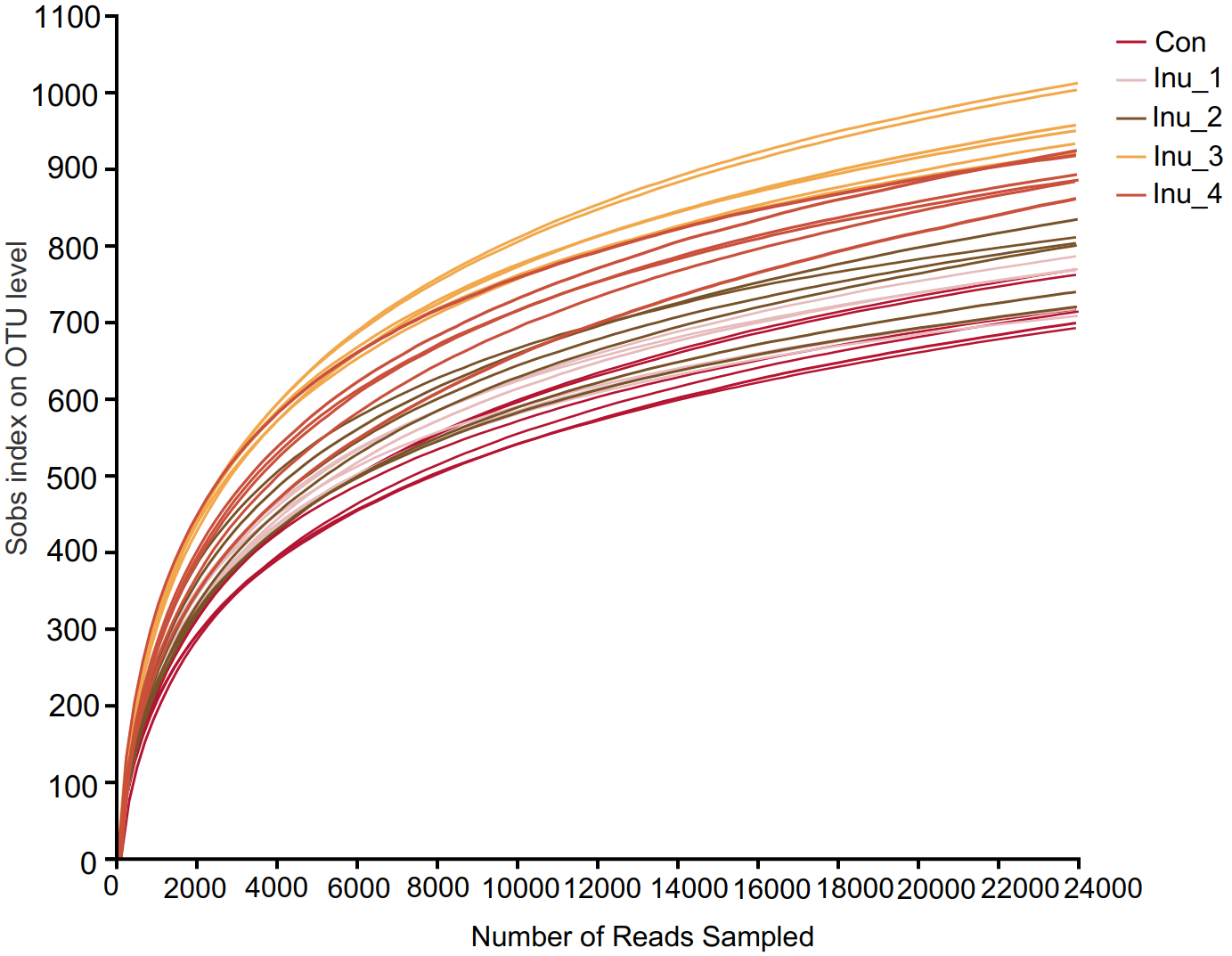

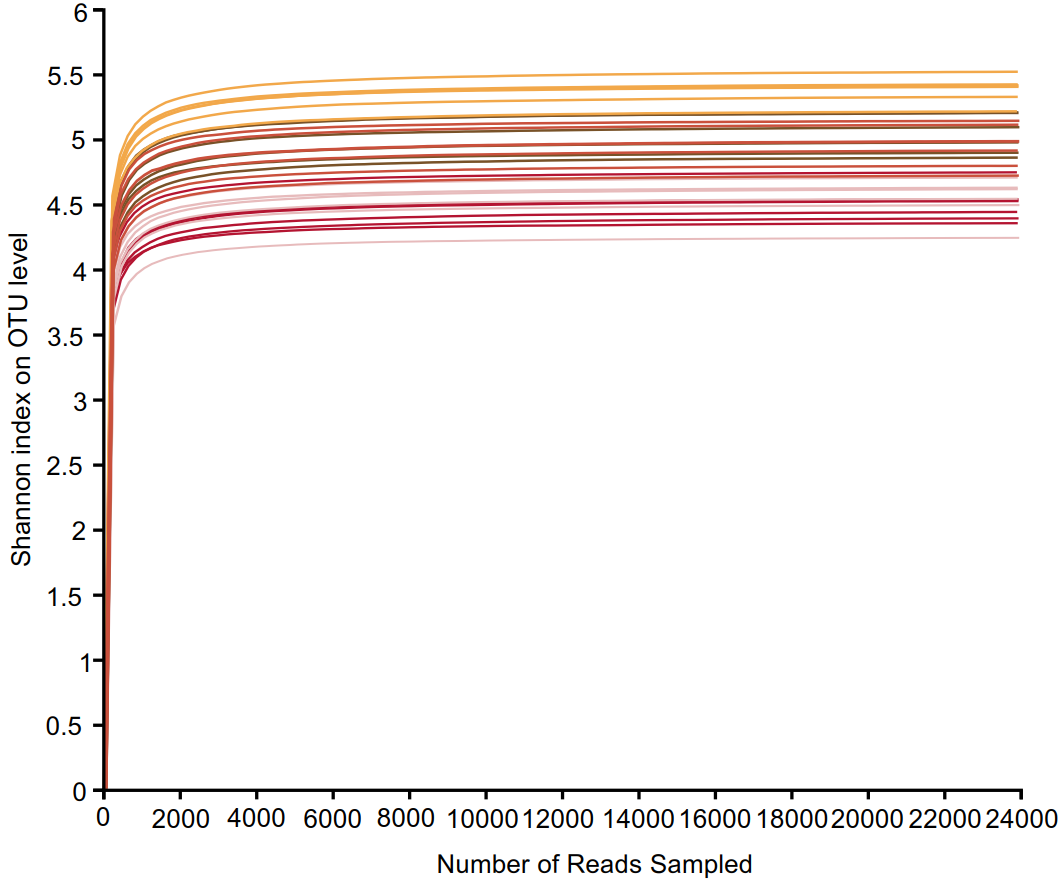


a

b

Figure S1 Rarefaction curves of fecal microbiota based on (a) Sobs and (b) Shannon indexes. Con = control group; Inu-1 = inulin-1 group, the inulin addition level was 100 g/d per cow; Inu-2 = inulin-2 group, the inulin addition level was 200 g/d per cow; Inu-3 = inulin-3 group, the inulin addition level was 300 g/d per cow; Inu-4 = inulin-4 group, the inulin addition level was 400 g/d per cow.


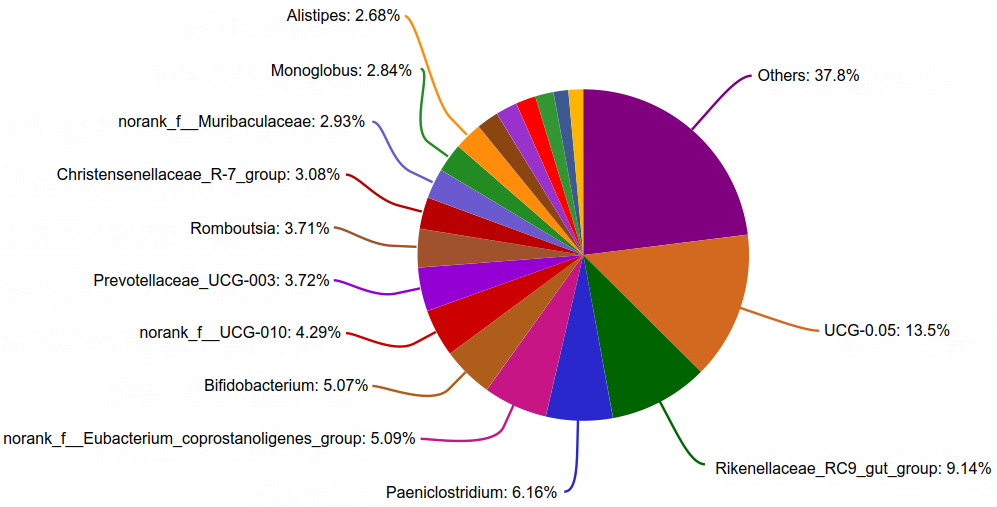

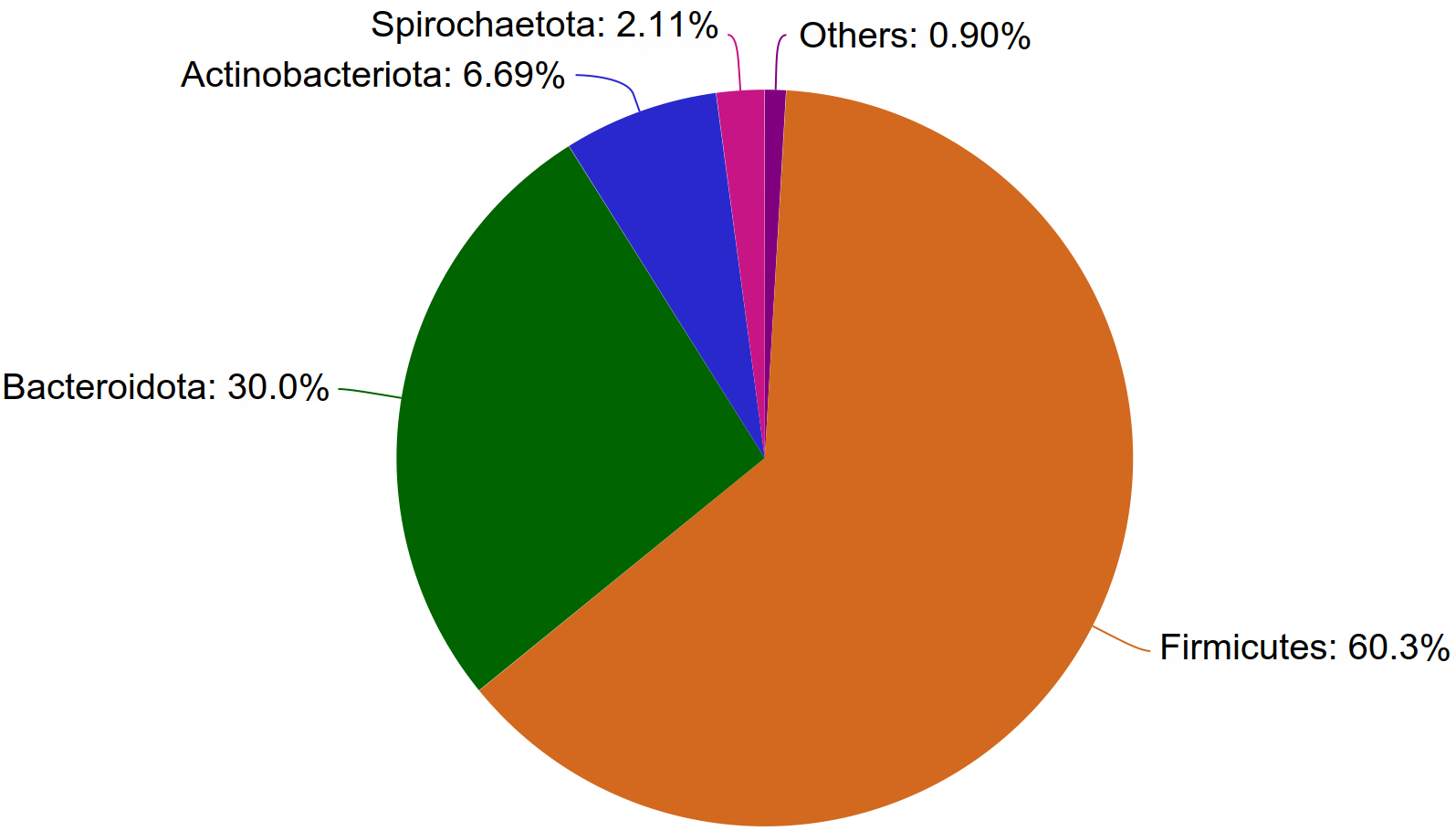


b

a

c


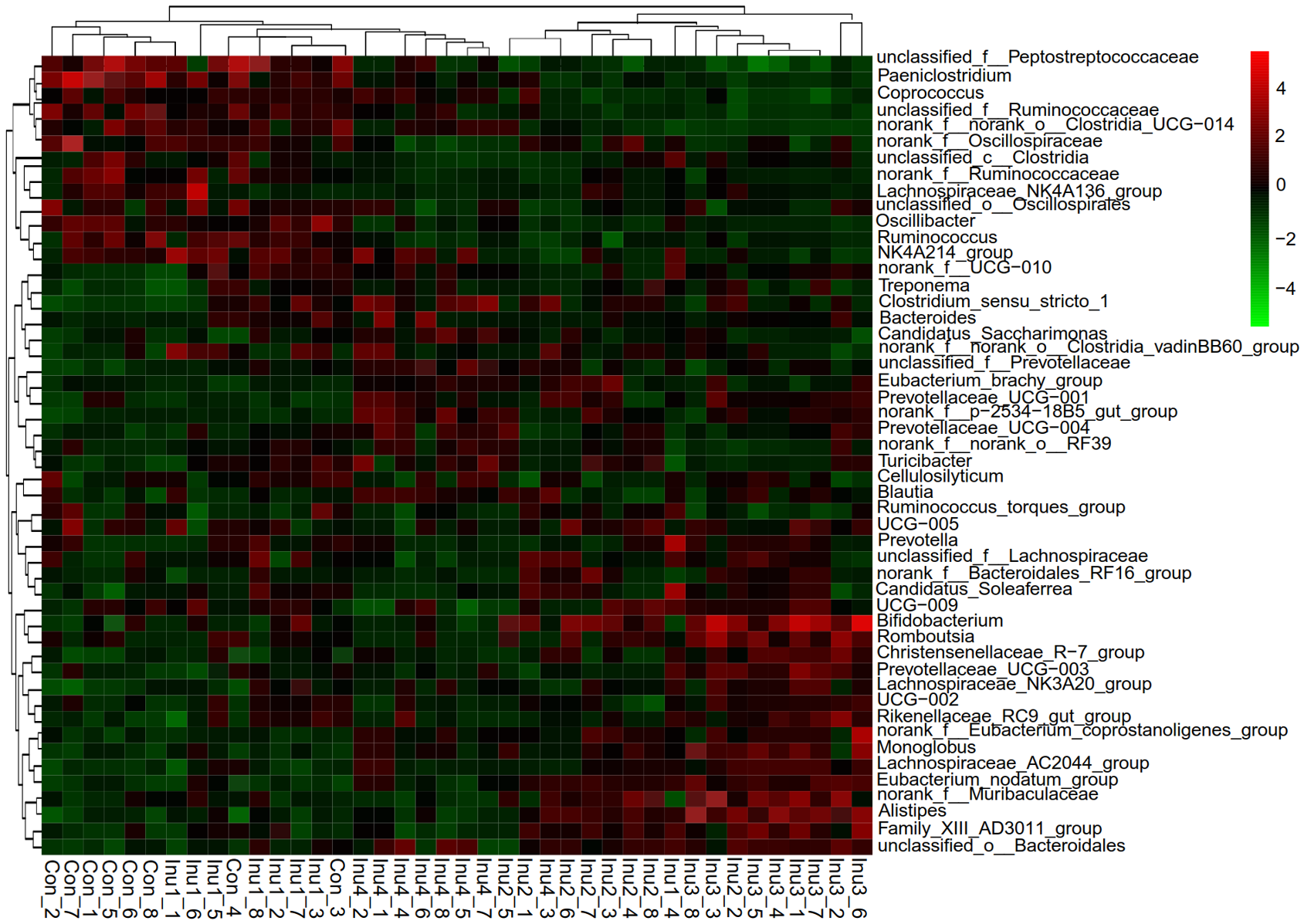


Figure S2 Fecal microbiota composition at (a) phylum and (b) genus level as well as (c) hierarchical clustering analysis (HCA) at genus level. Each row represents a genus of bacteria and each column represents a sample. The color in each cell means the relative abundance of this bacteria measured in rumen, red represents high level of relative abundance while green means the low relative abundance. Con = control group; Inu-1 = inulin-1 group, the inulin addition level was 100 g/d per cow; Inu-2 = inulin-2 group, the inulin addition level was 200 g/d per cow; Inu-3 = inulin-3 group, the inulin addition level was 300 g/d per cow; Inu-4 = inulin-4 group, the inulin addition level was 400 g/d per cow.


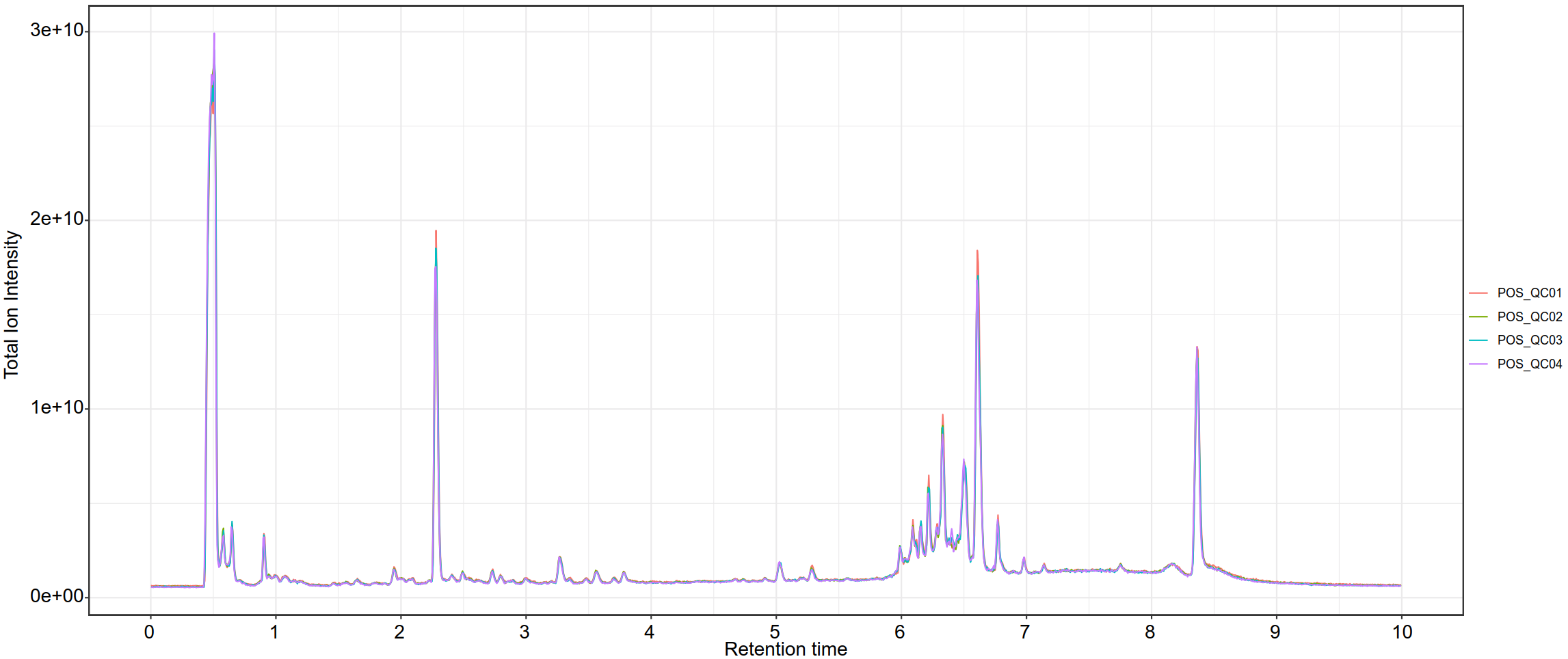


a


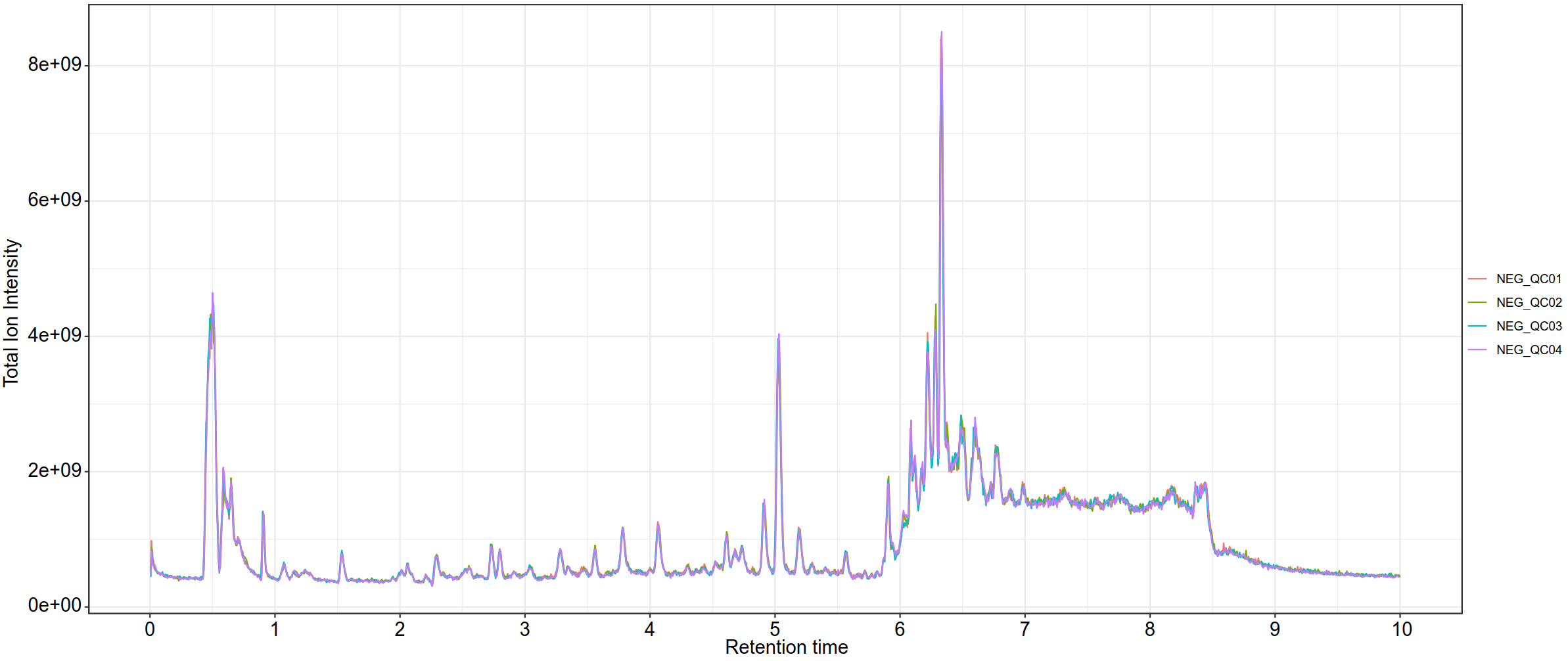


b

c


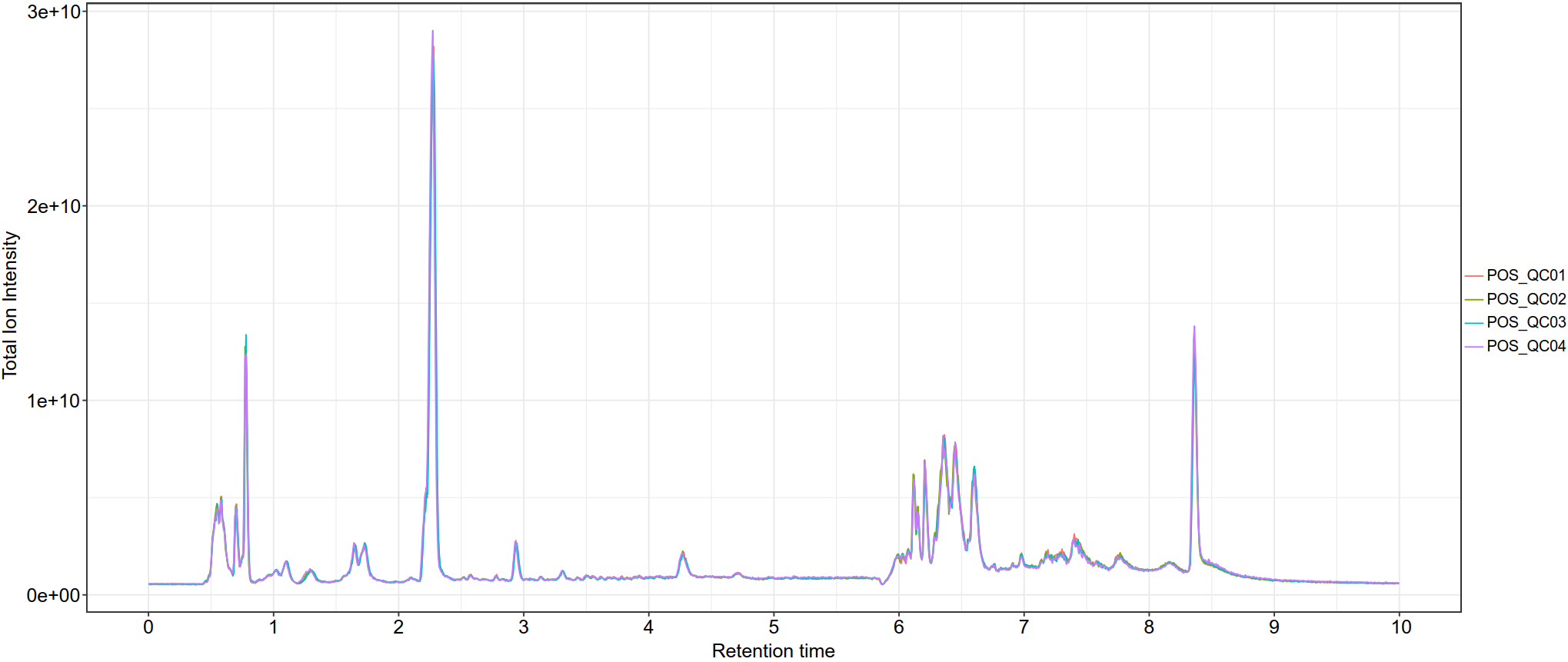


d


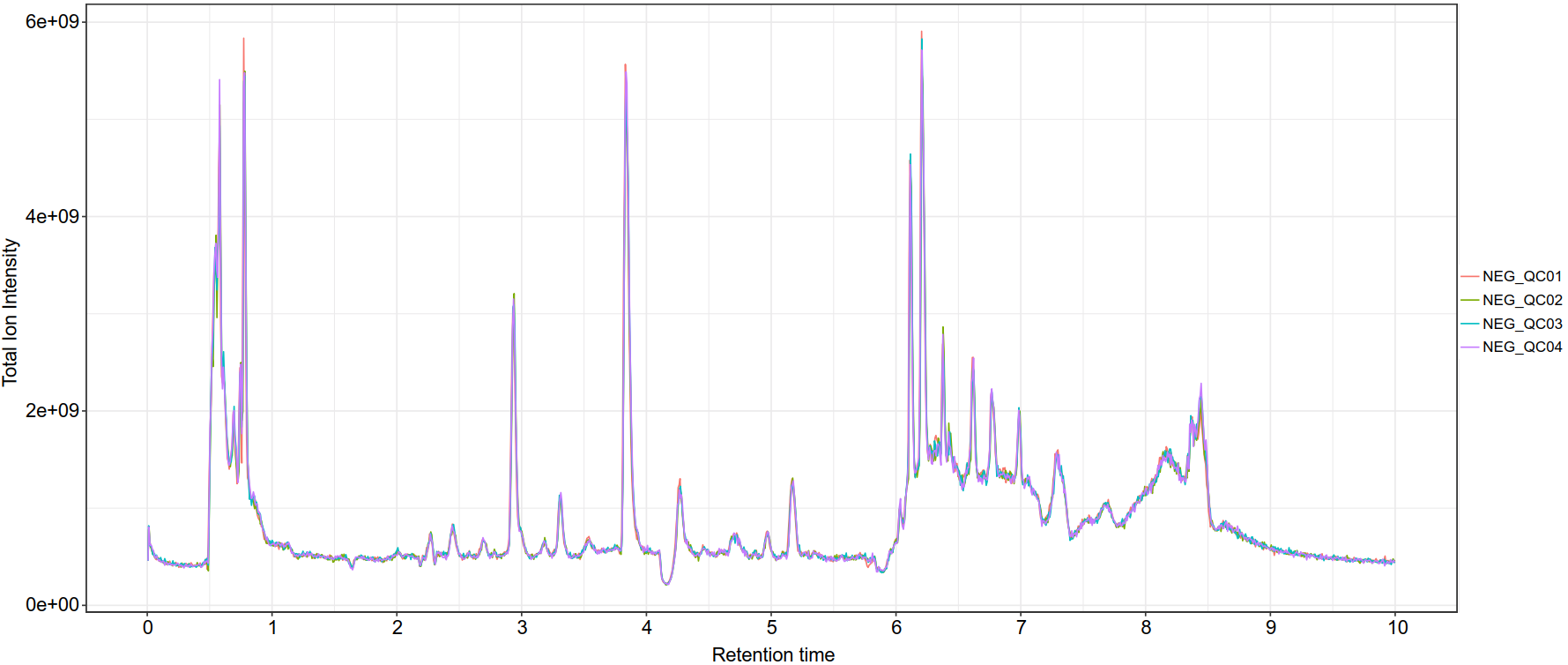


Figure S3 Total ion chromatogram (TIC) of the quality control (QC) sample in (a and b) feces and (c and d) serum. (A and C) the positive ion modes; (b and d) negative ion modes.


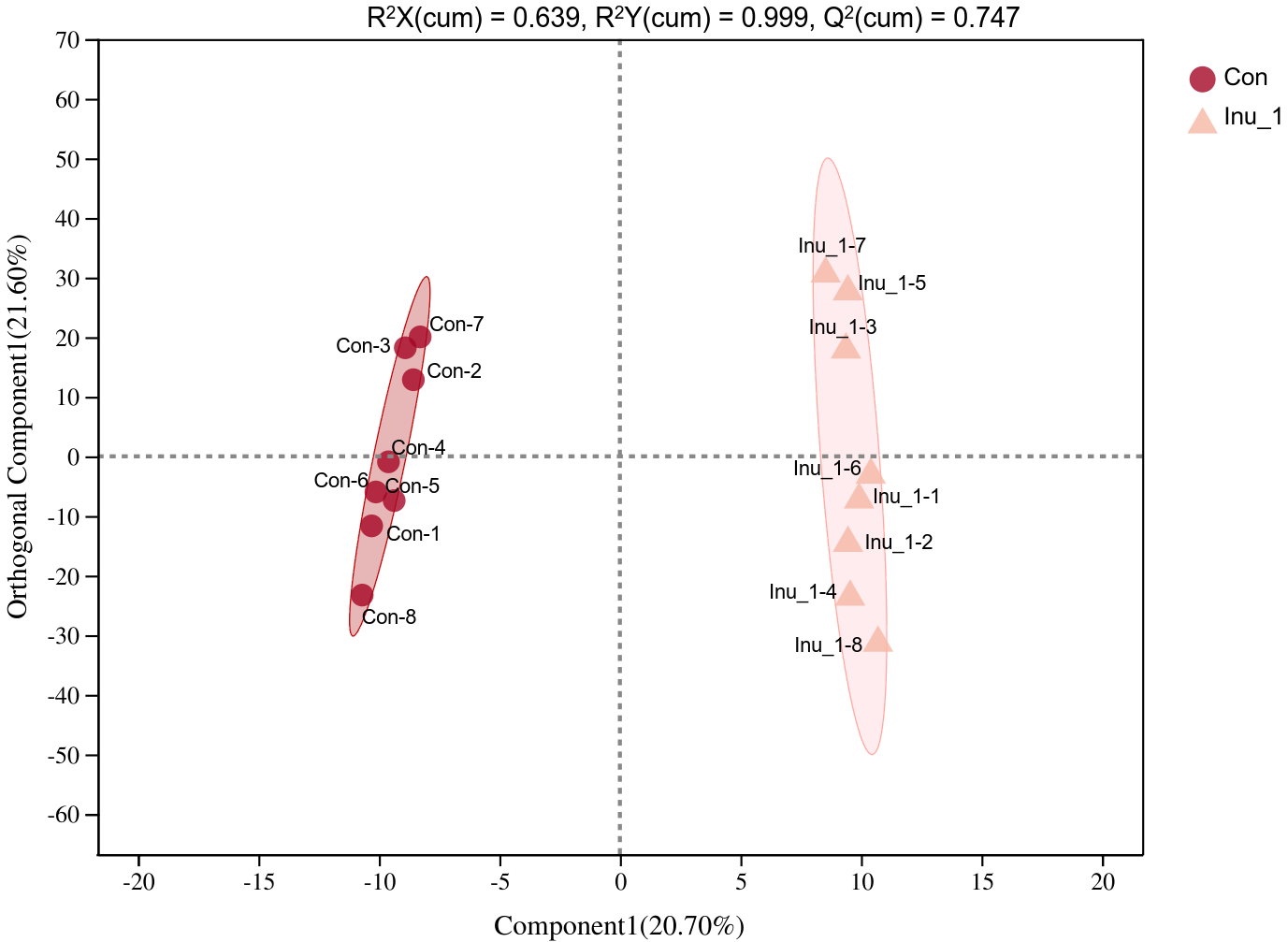

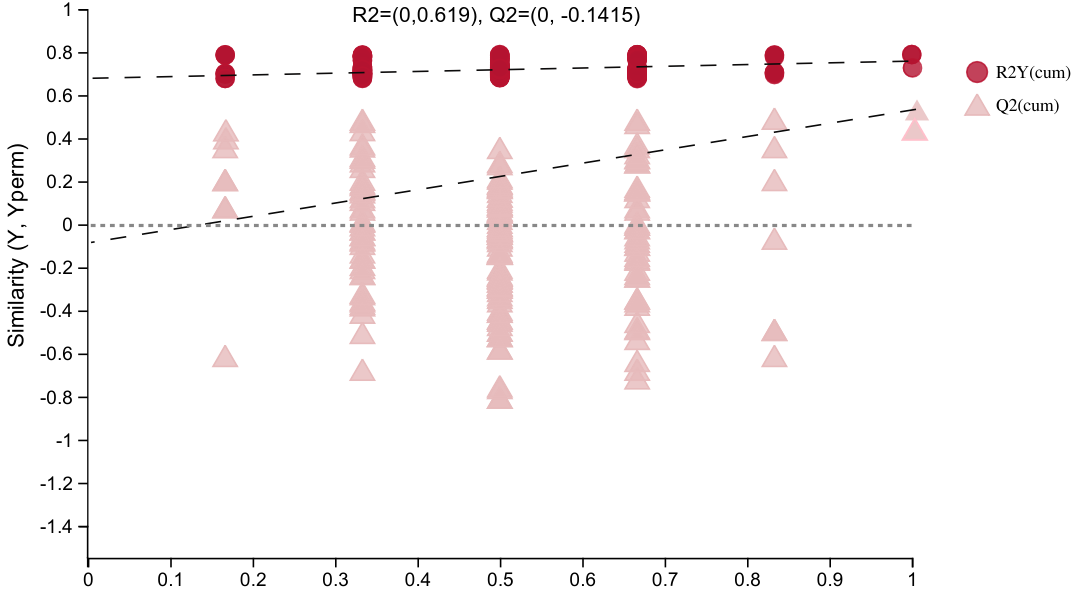


a

b


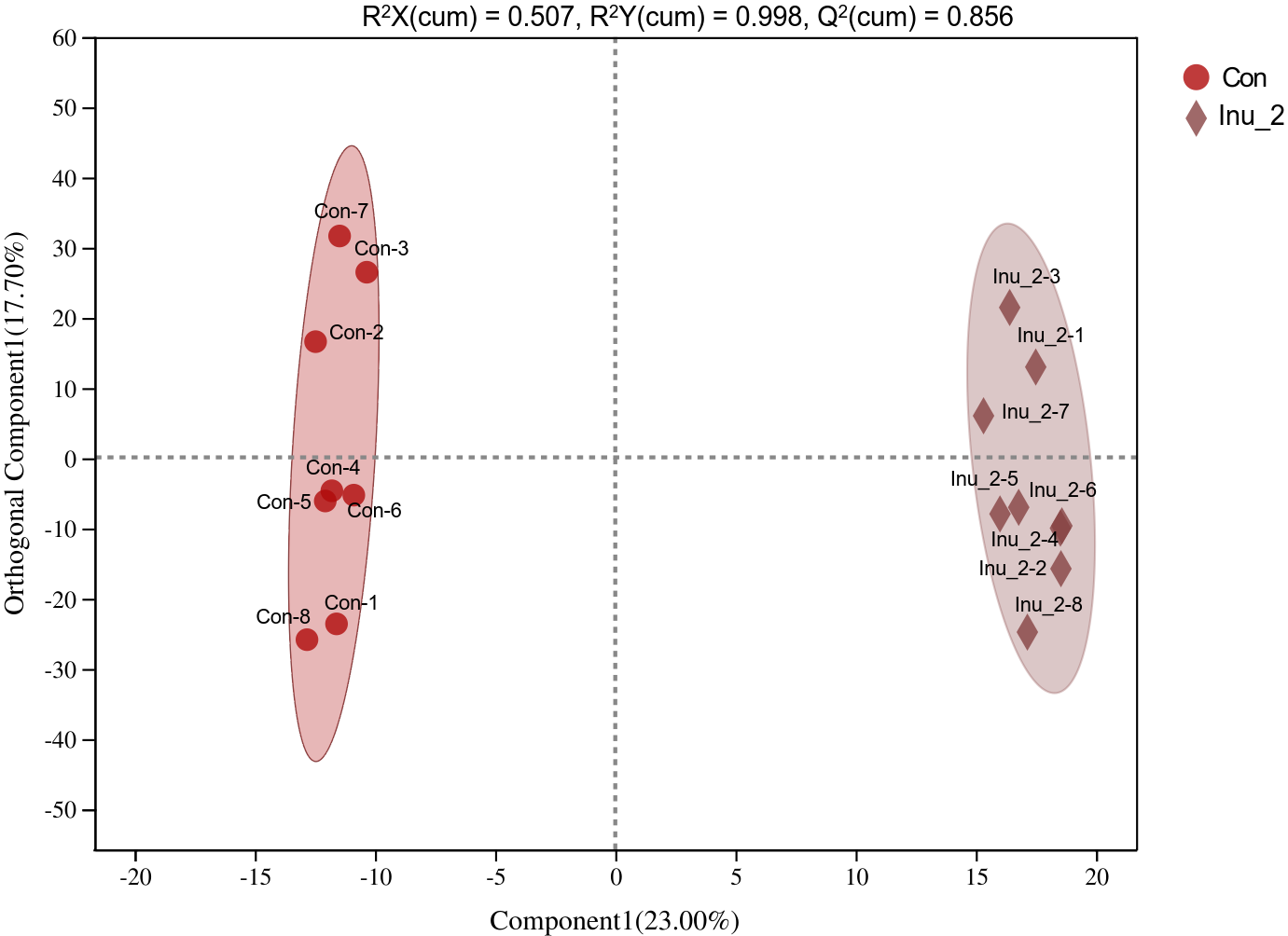


d

c


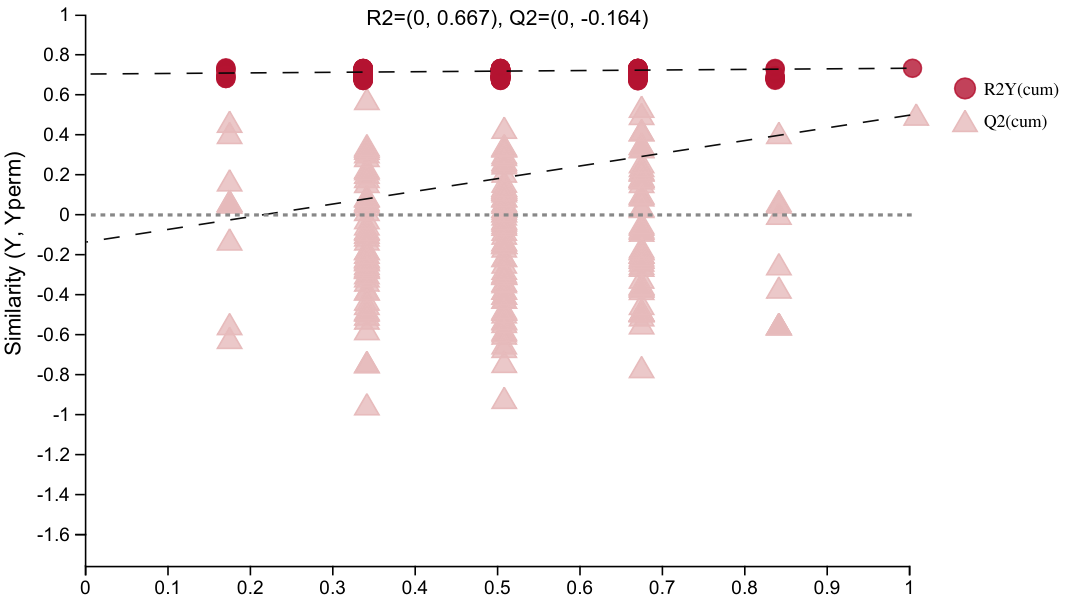


f

e


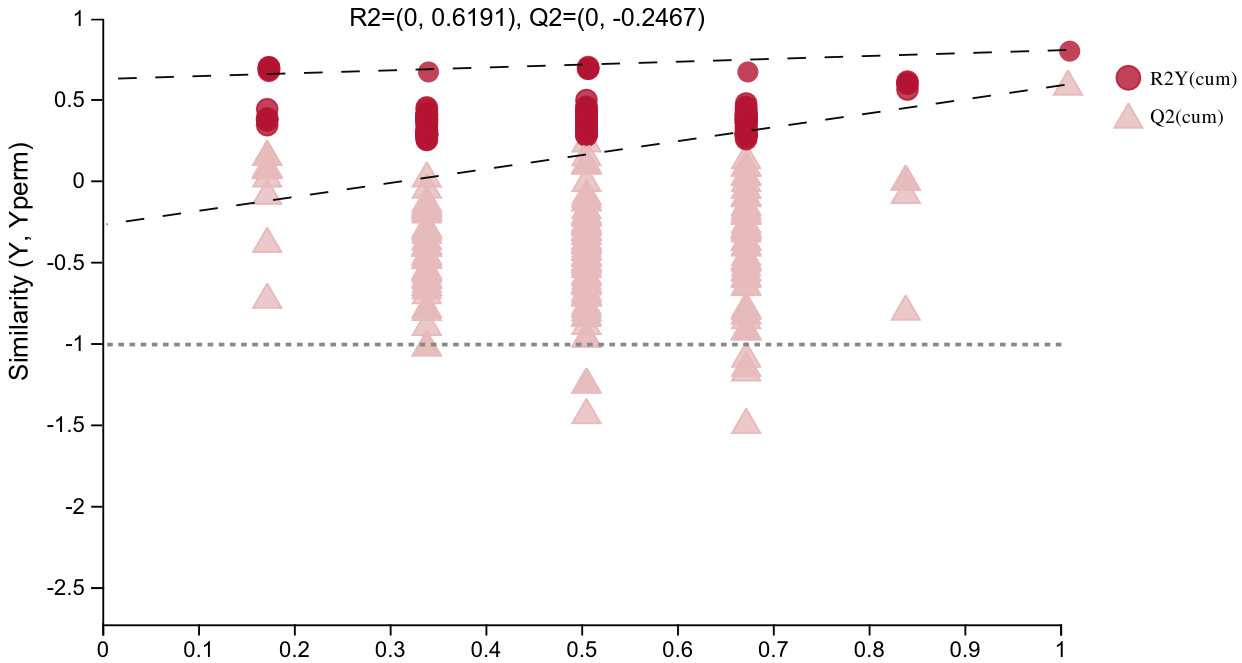

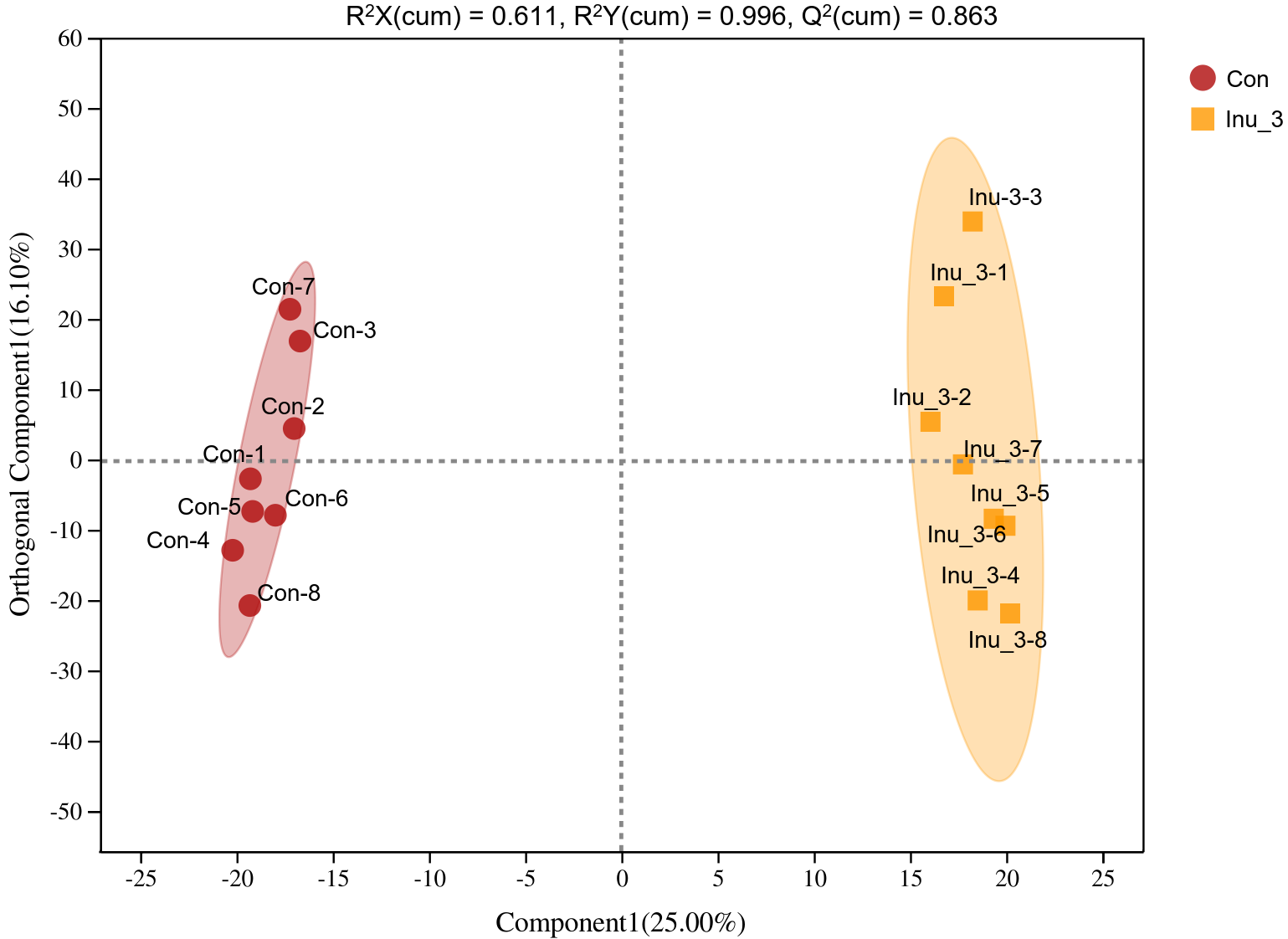


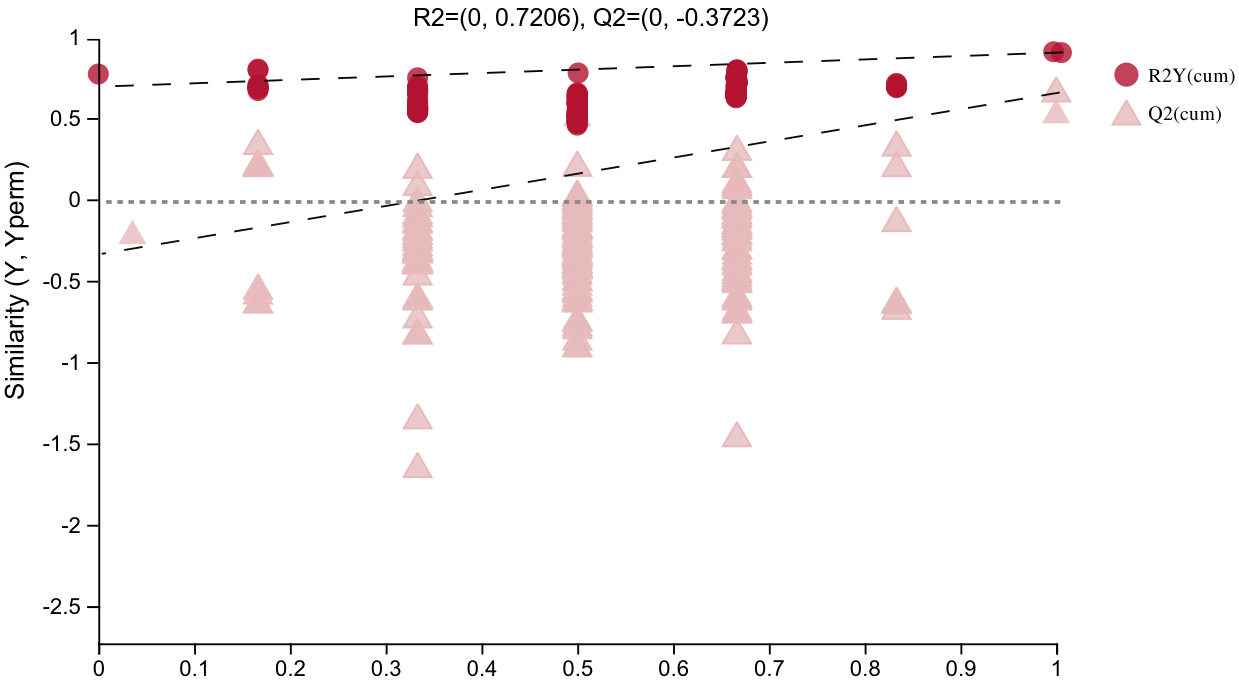

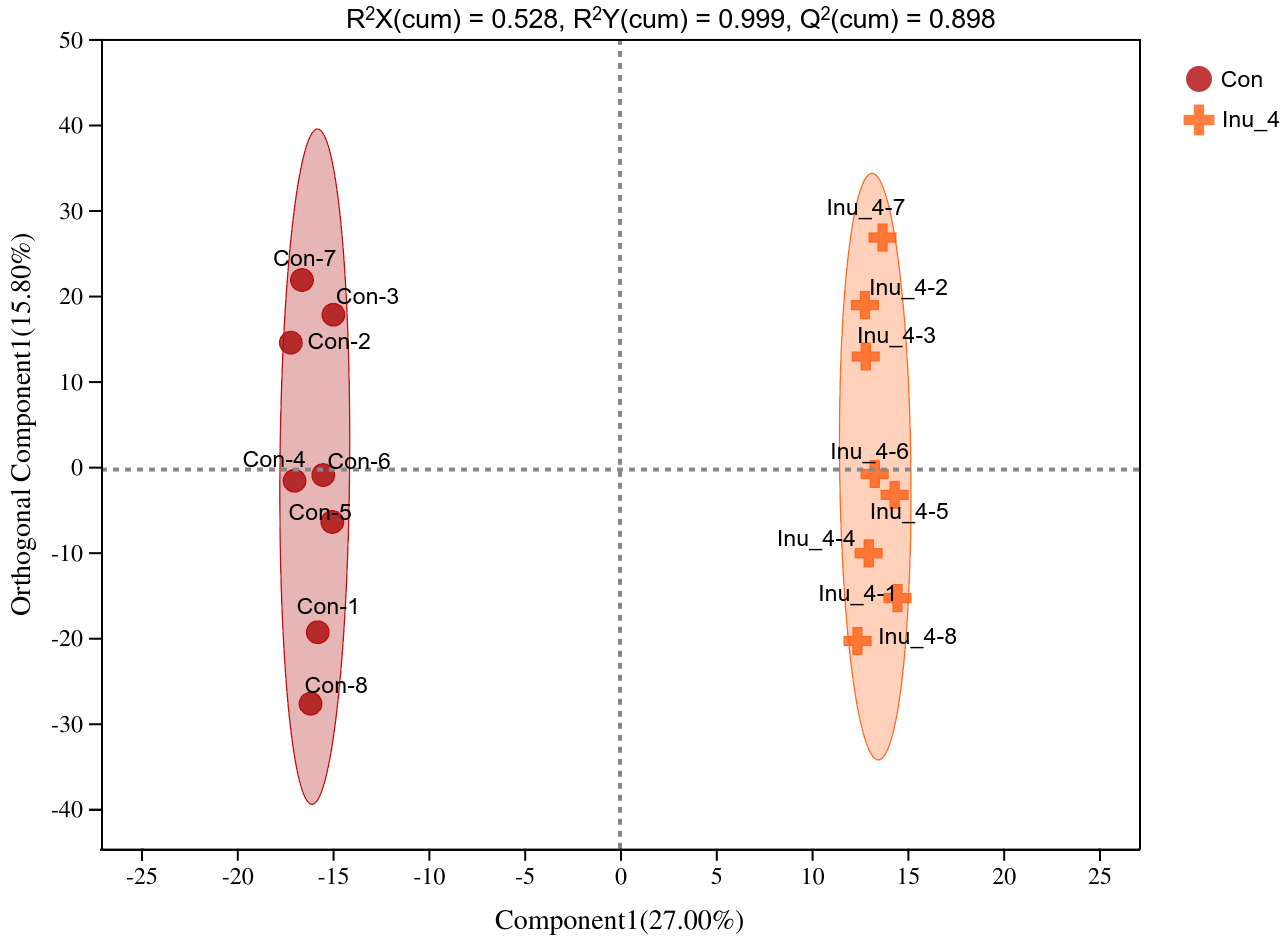


h

g


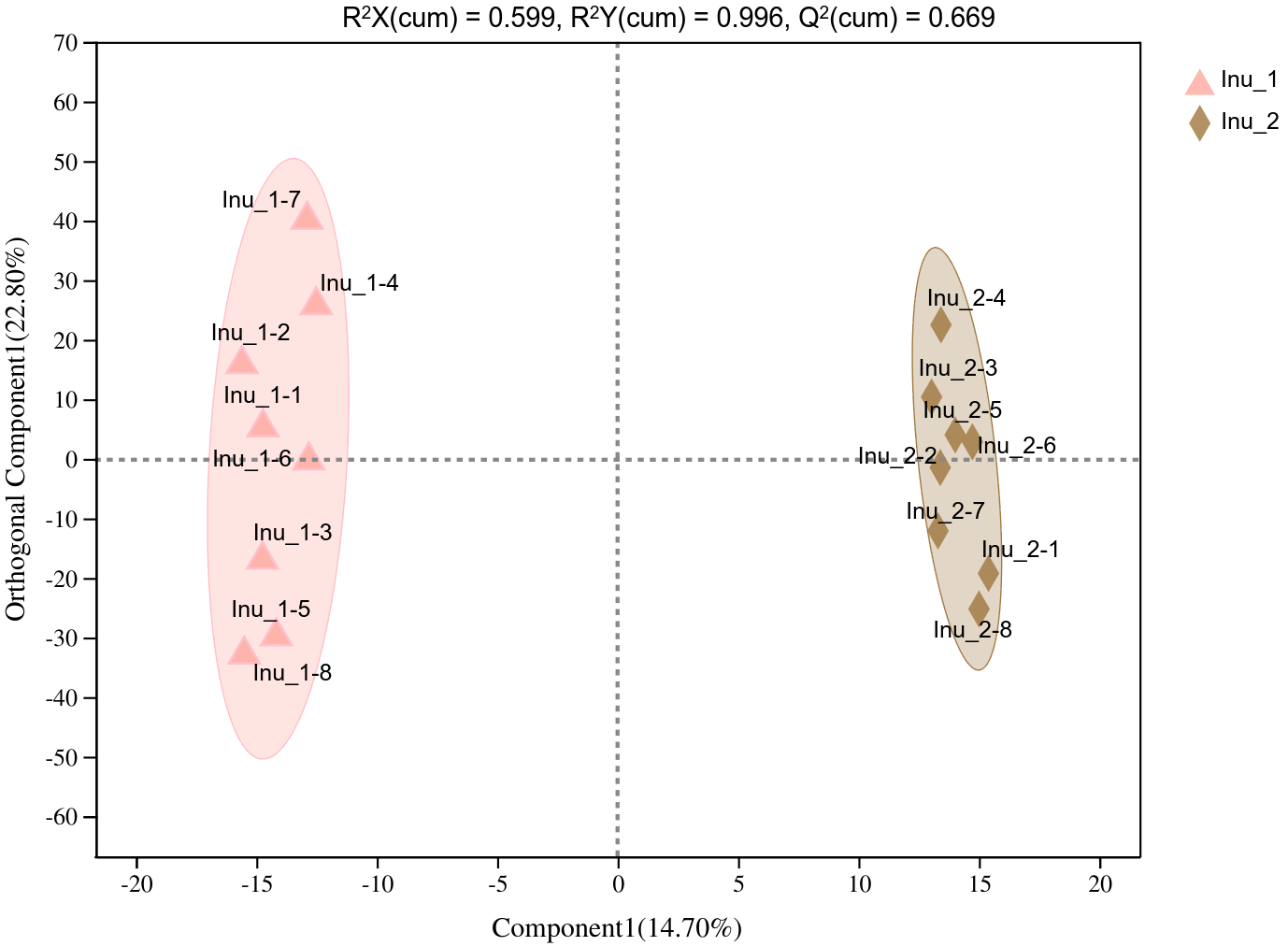

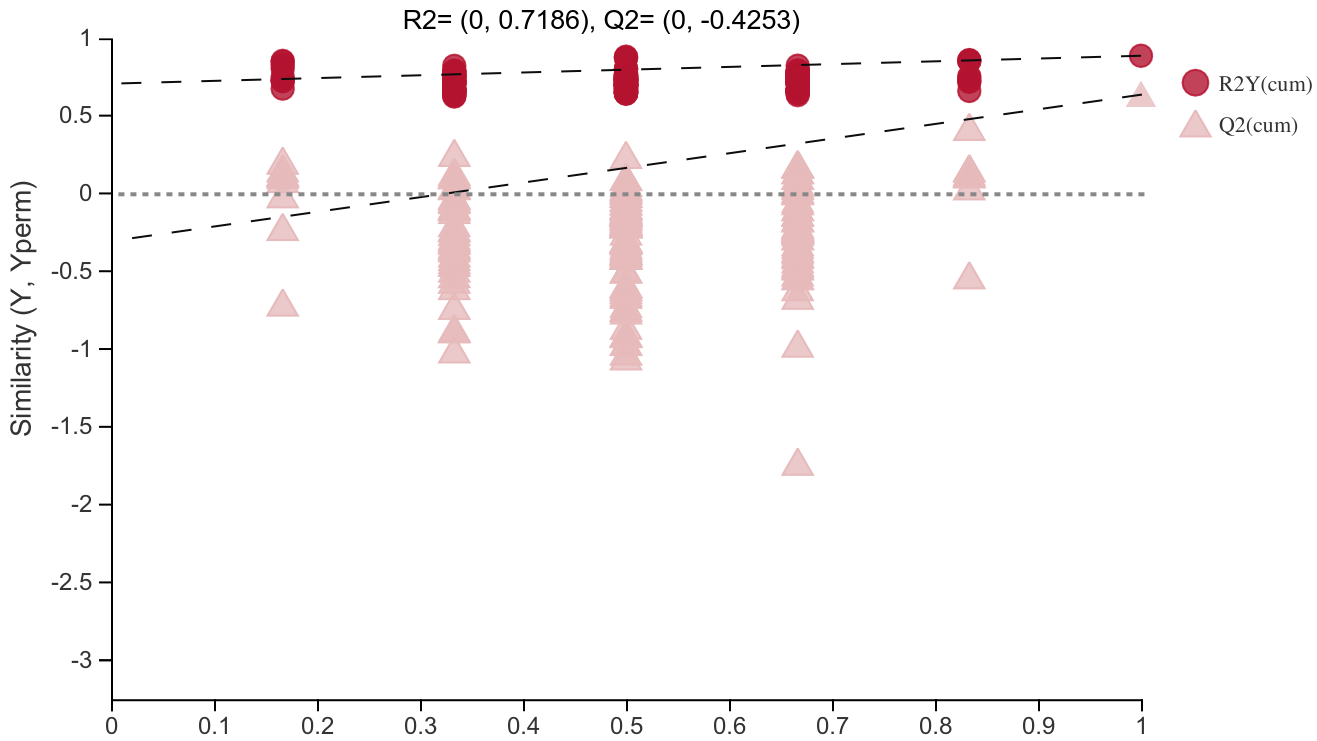


i

j


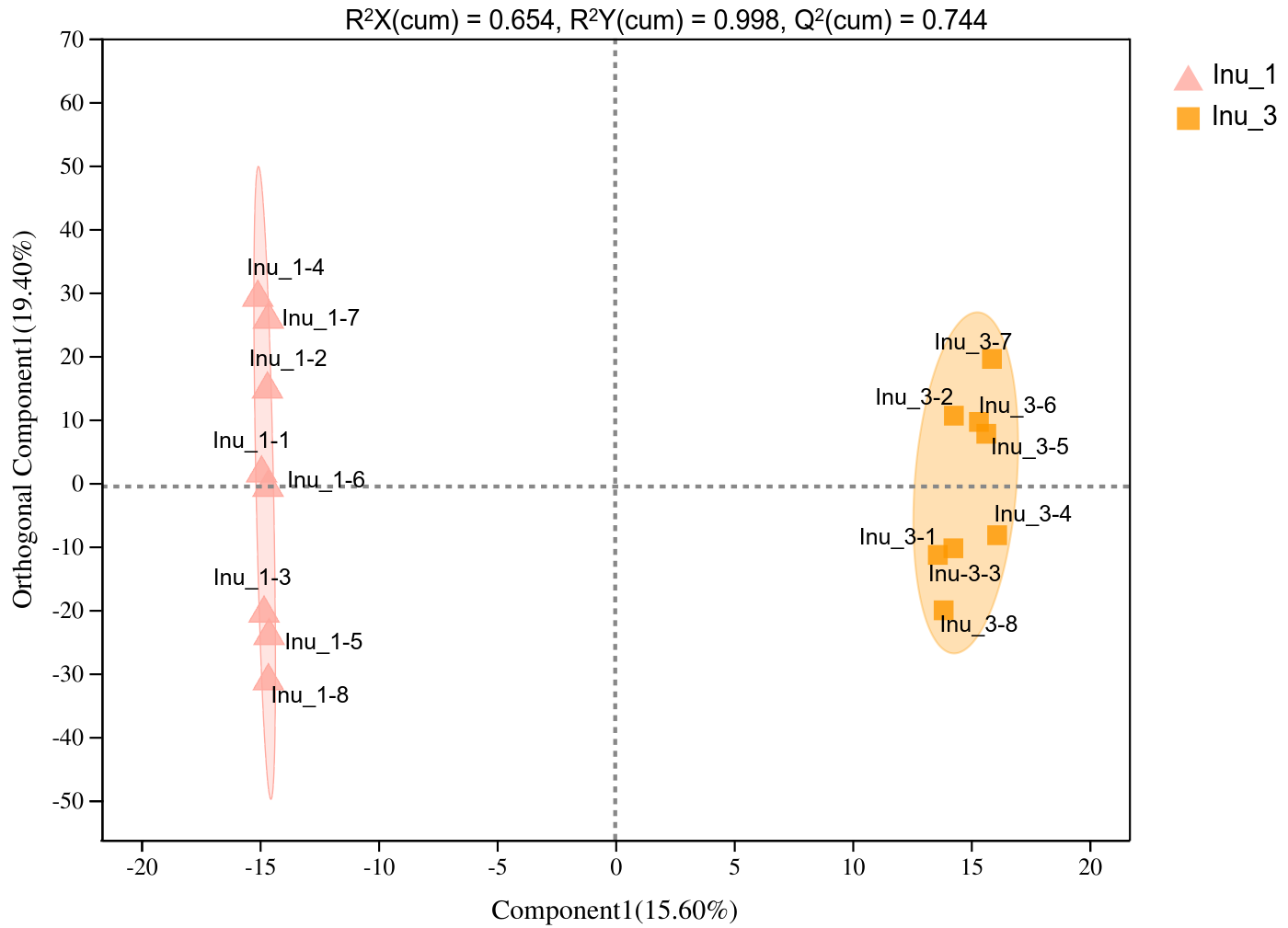

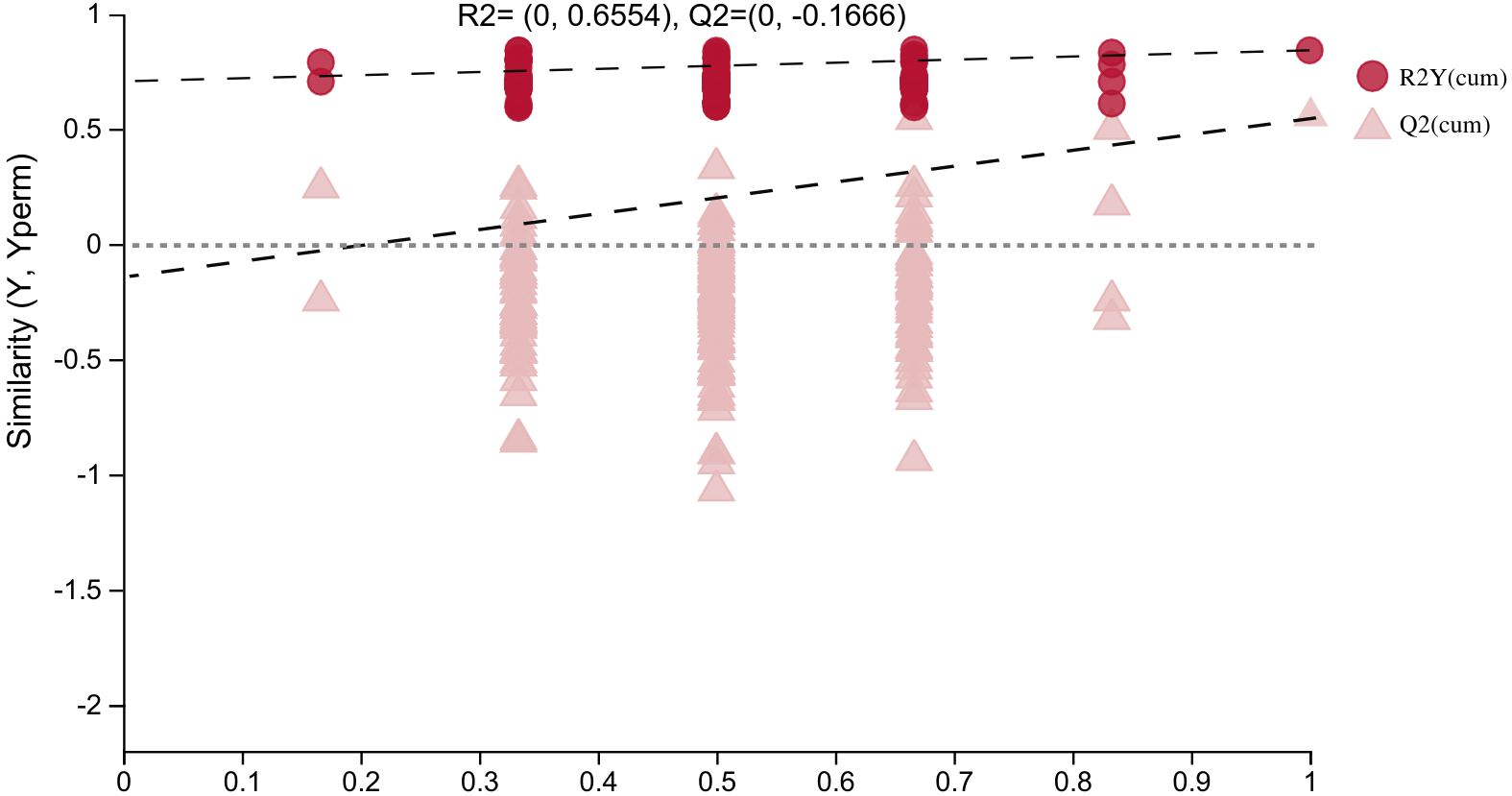


k

l

n

m


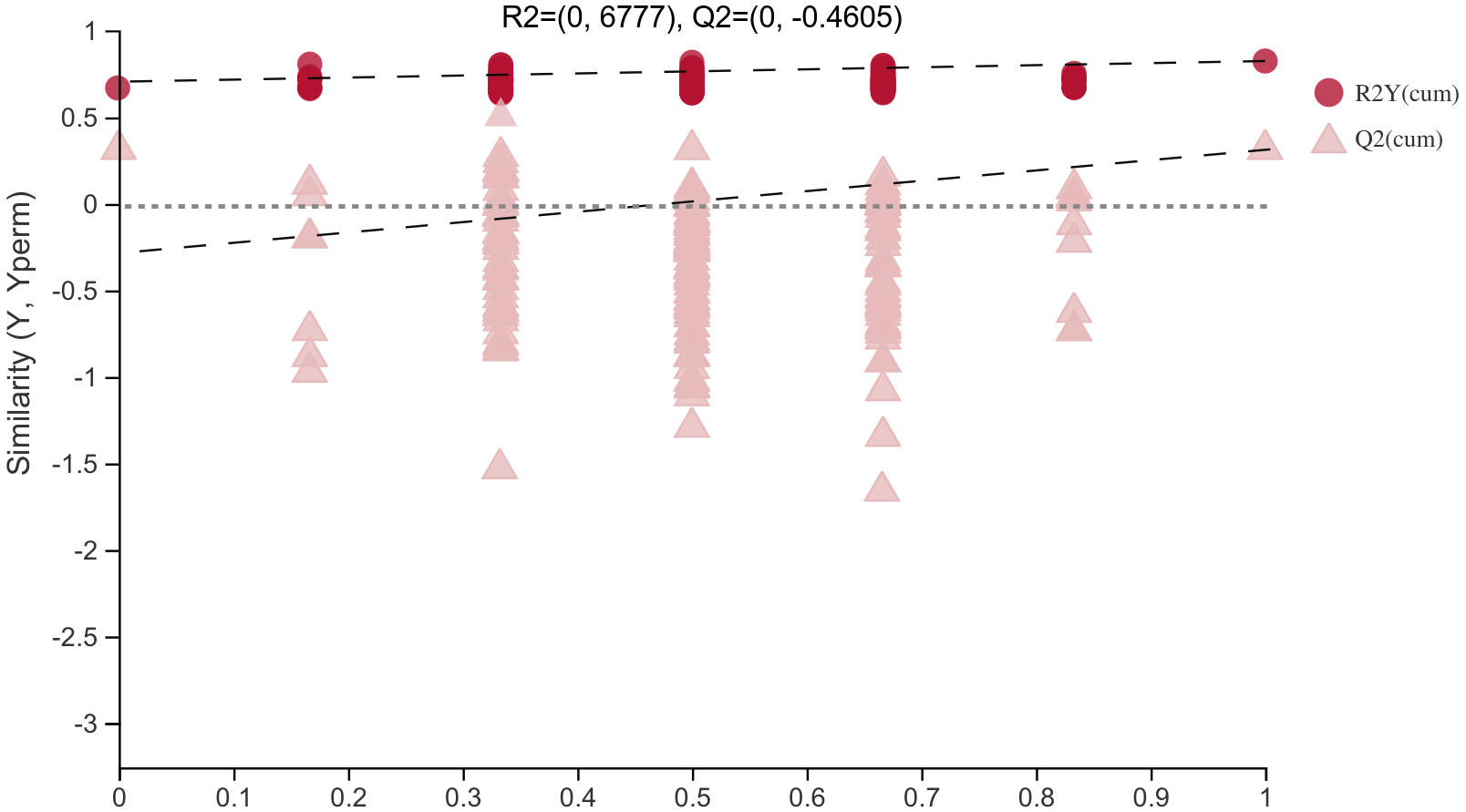

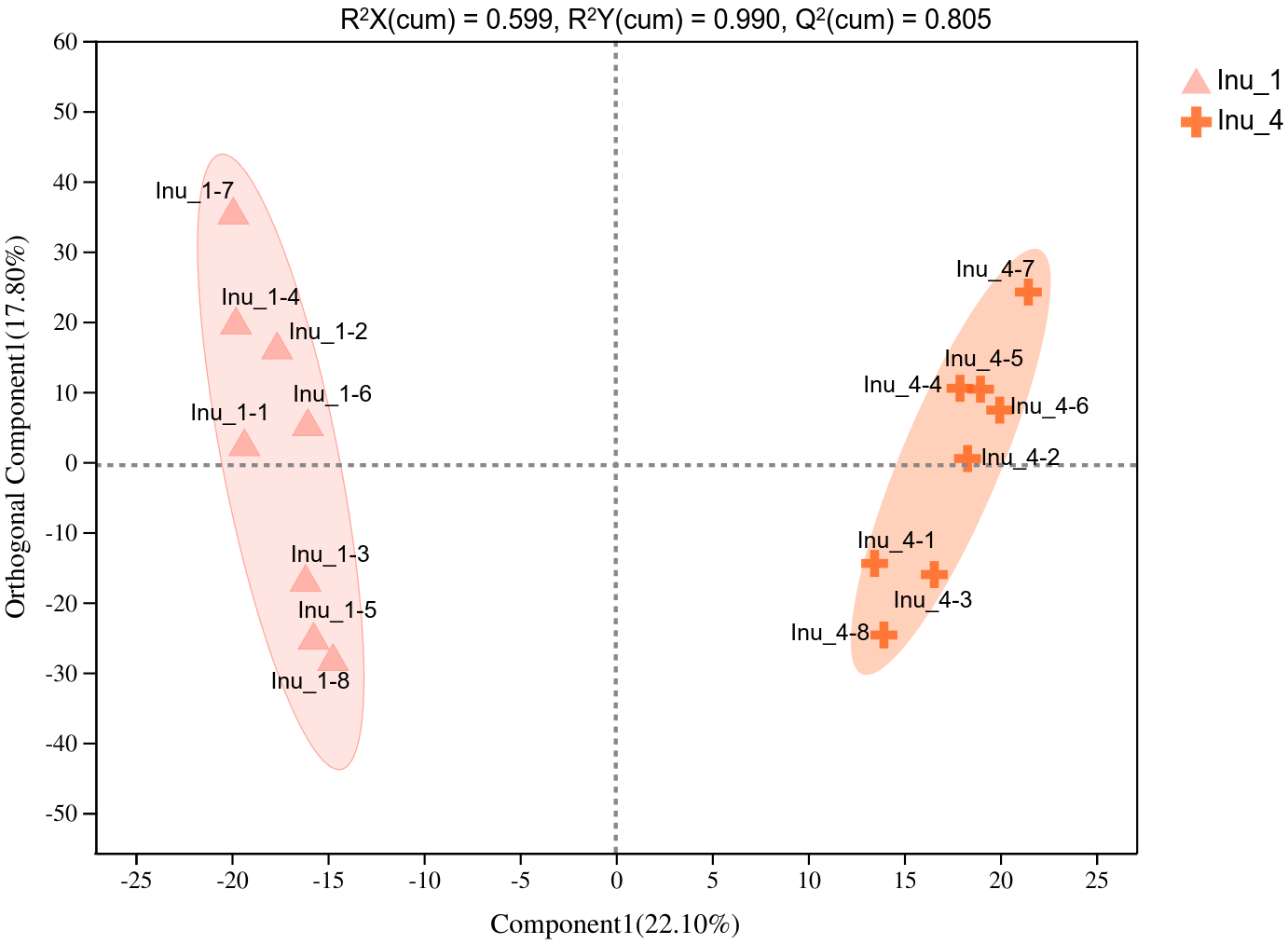


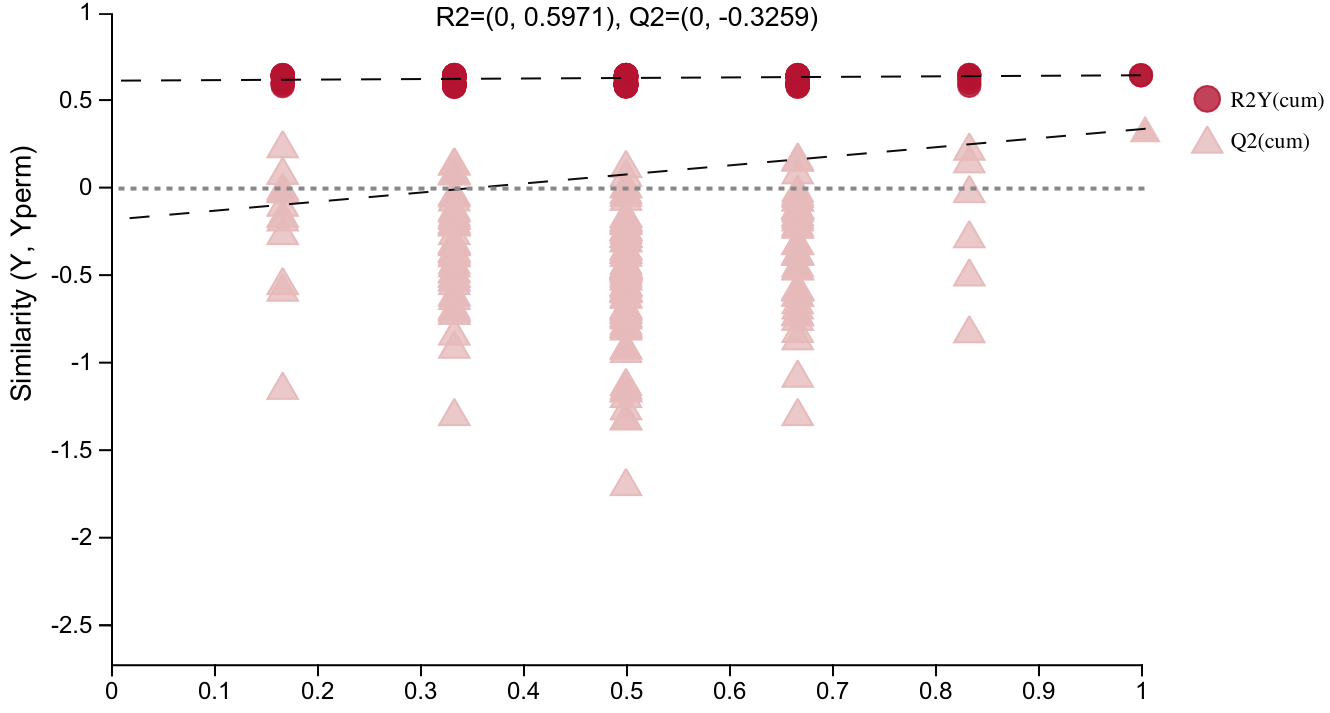


p

o


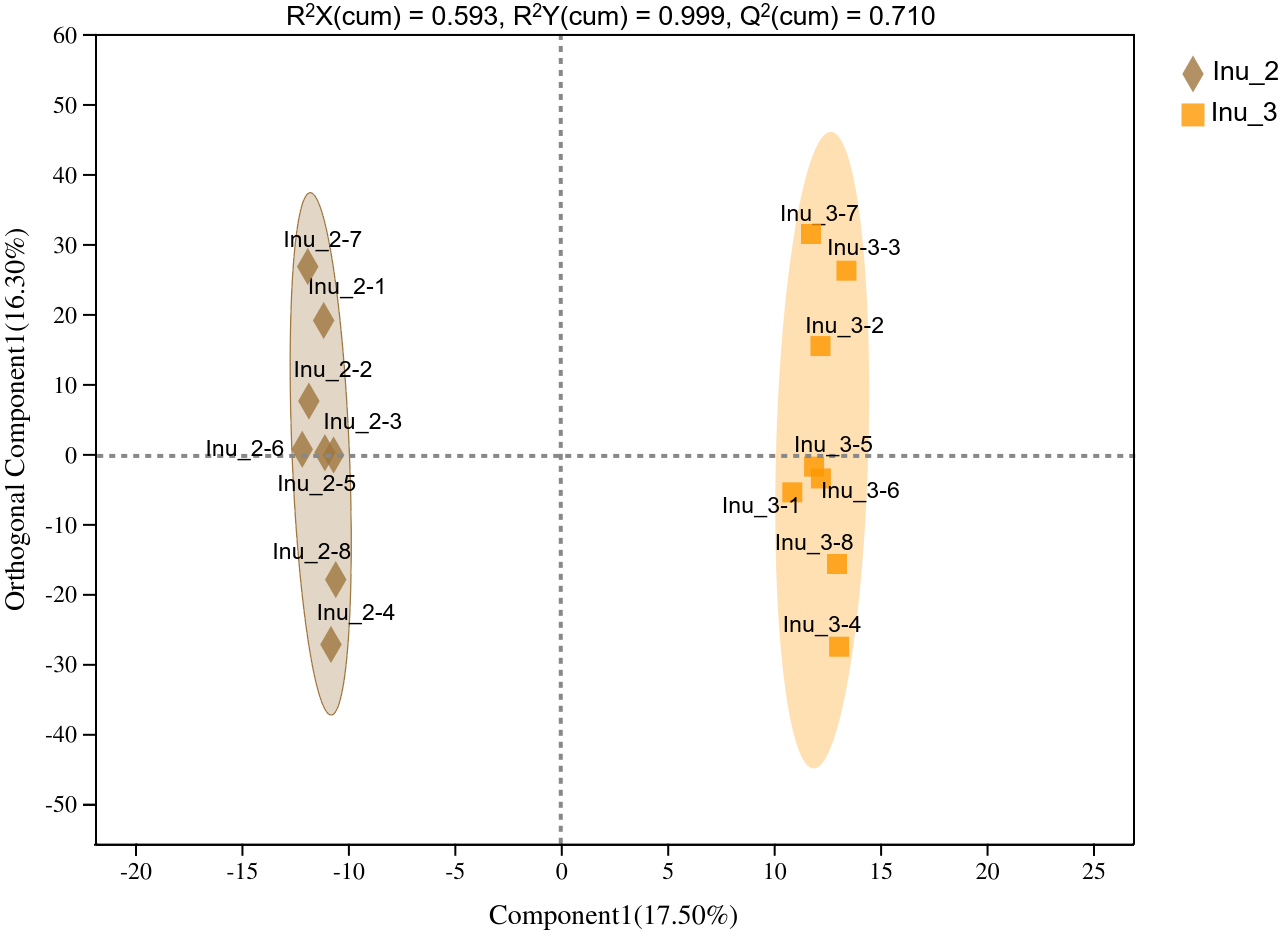


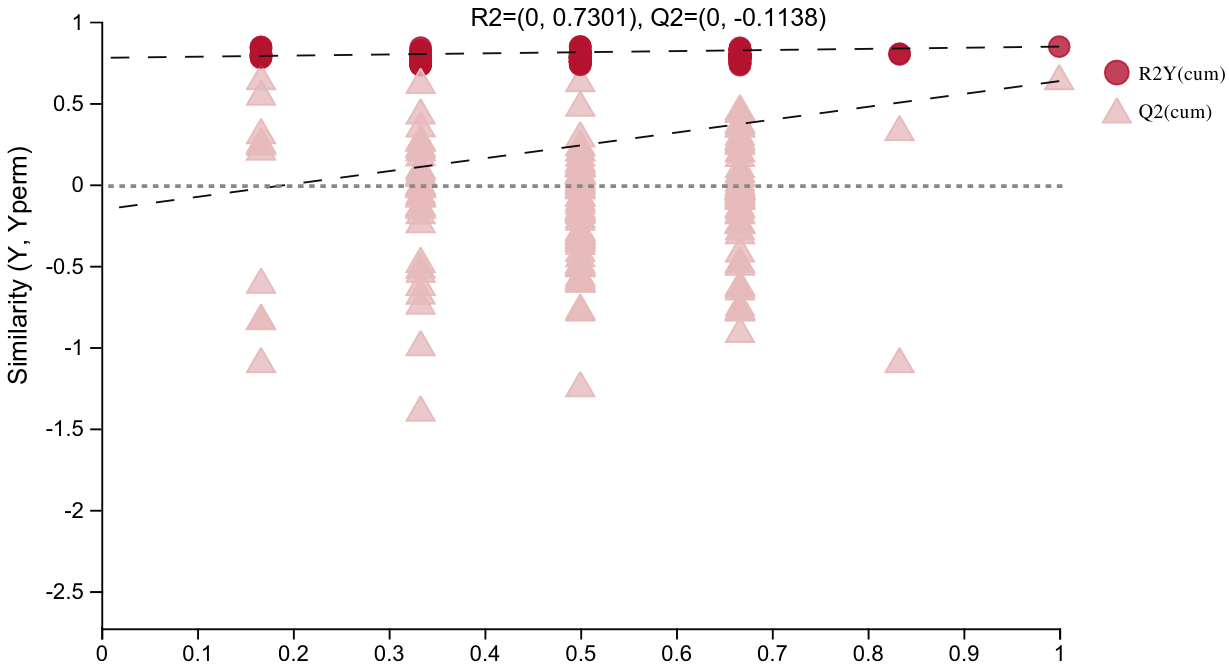

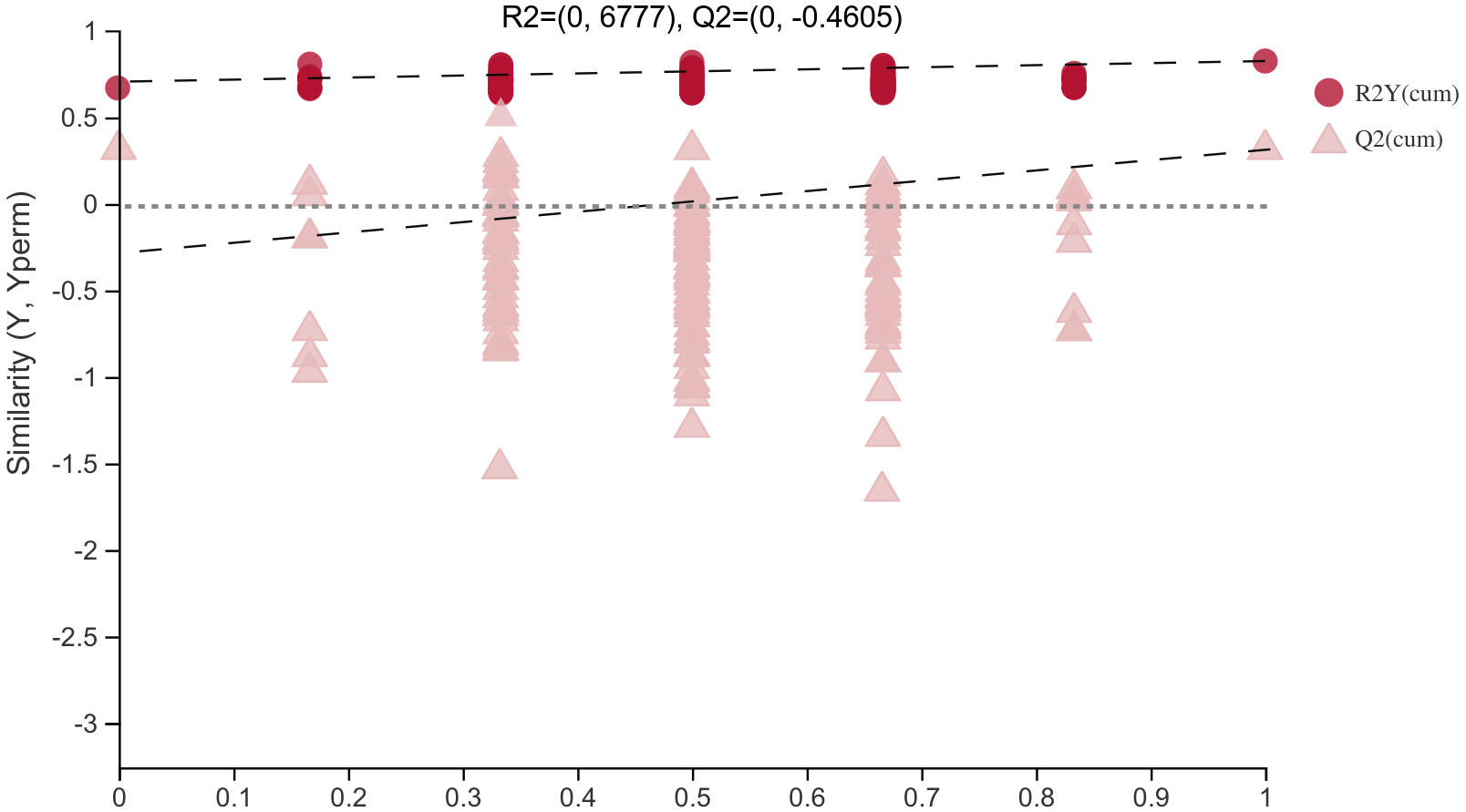

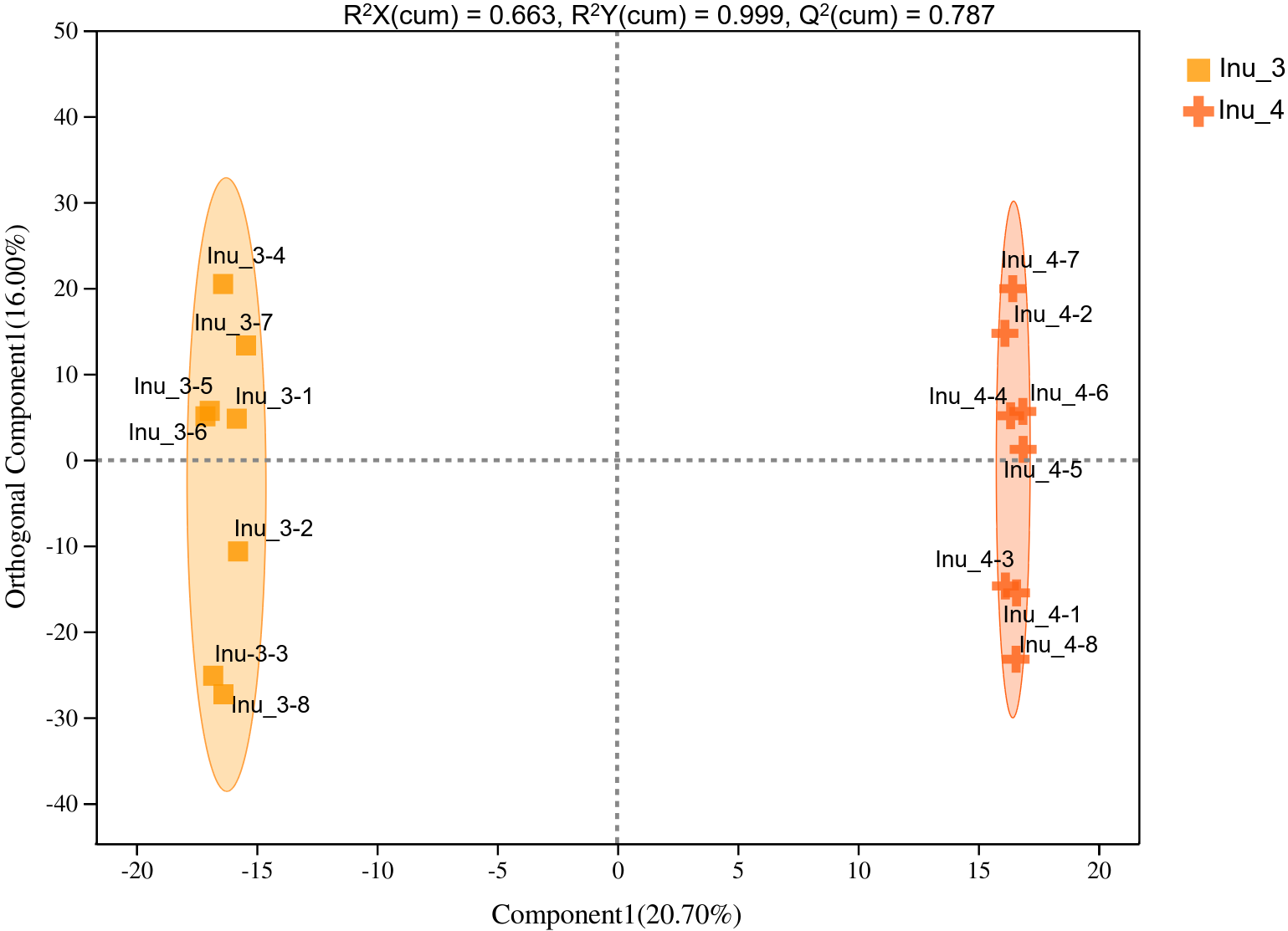

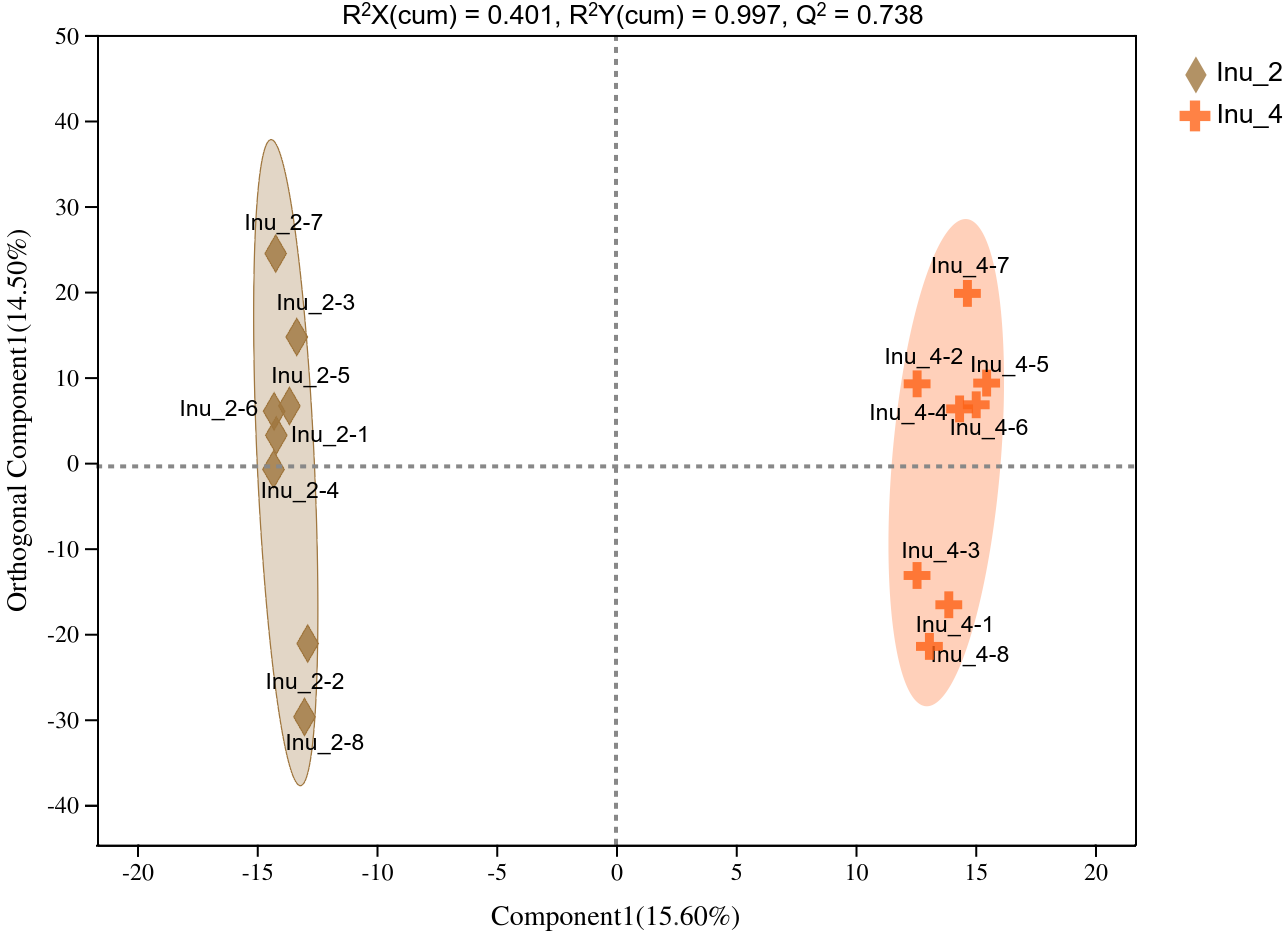


t

s

q

r

Figure S4 Orthogonal partial least squares discrimination analysis (OPLS-DA) score plot (A, C, E, G, I, K, M, O, Q, S) and OPLS-DA permutation testing (B, D, F, H, J, L, N, P, R, T) of fecal metabolites in control and different inulin addition groups. R^2^X(cum) and R^2^Y(cum) represent the cumulative interpretation rate to the X and Y matrices of the model, respectively; Q^2^(cum) represents the predictive ability of the model. The closer these three indicators are to 1, the more stable and reliable the model is. Q^2^(cum) > 0.5 indicates that the predictive ability of the model is better. Con = control group; Inu_1 = inulin-1 group, the inulin addition level was 100 g/d per cow; Inu_2 = inulin-2 group, the inulin addition level was 200 g/d per cow; Inu_3 = inulin-3 group, the inulin addition level was 300 g/d per cow; Inu_4 = inulin-4 group, the inulin addition level was 400 g/d per cow.


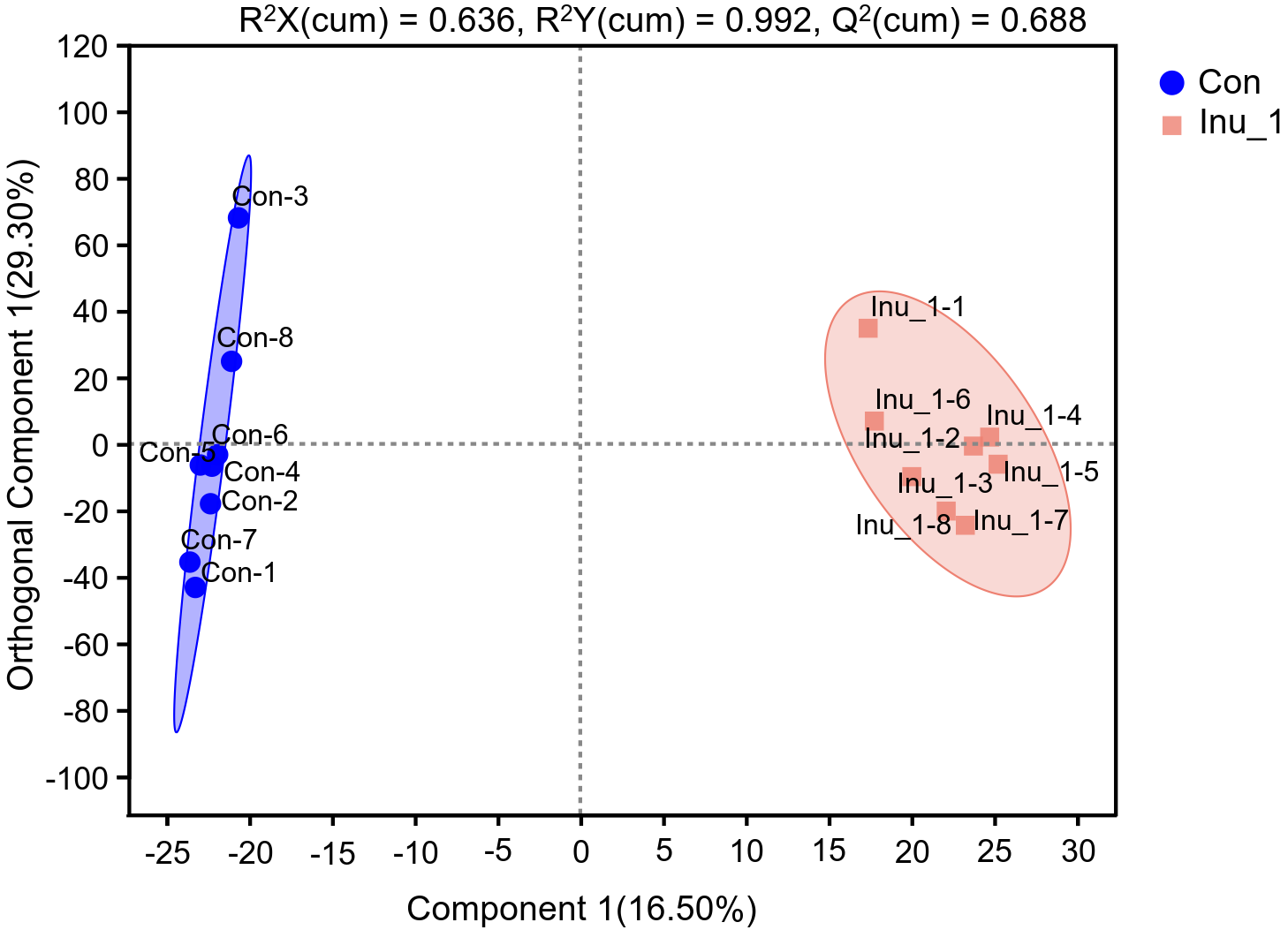


b

a


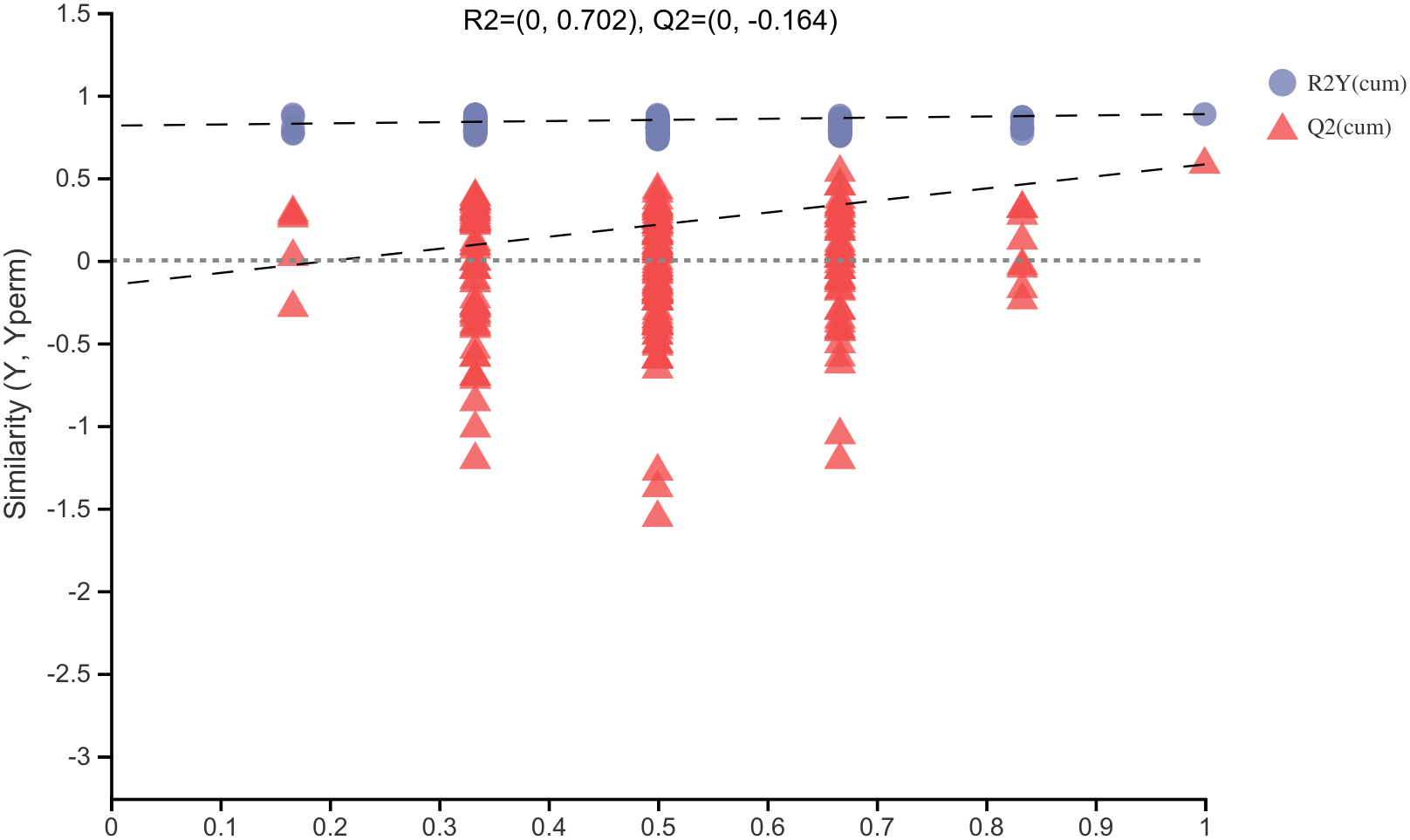


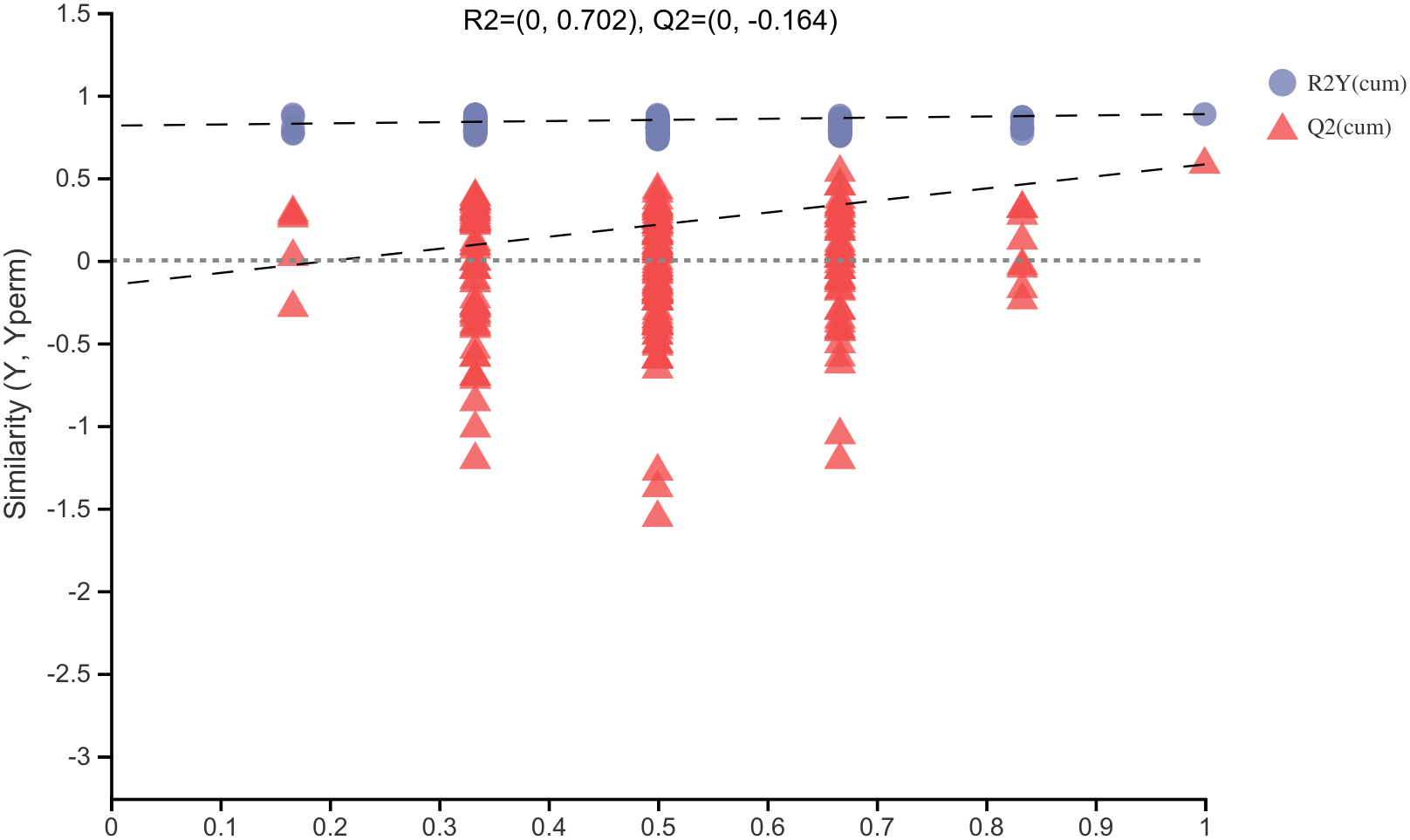


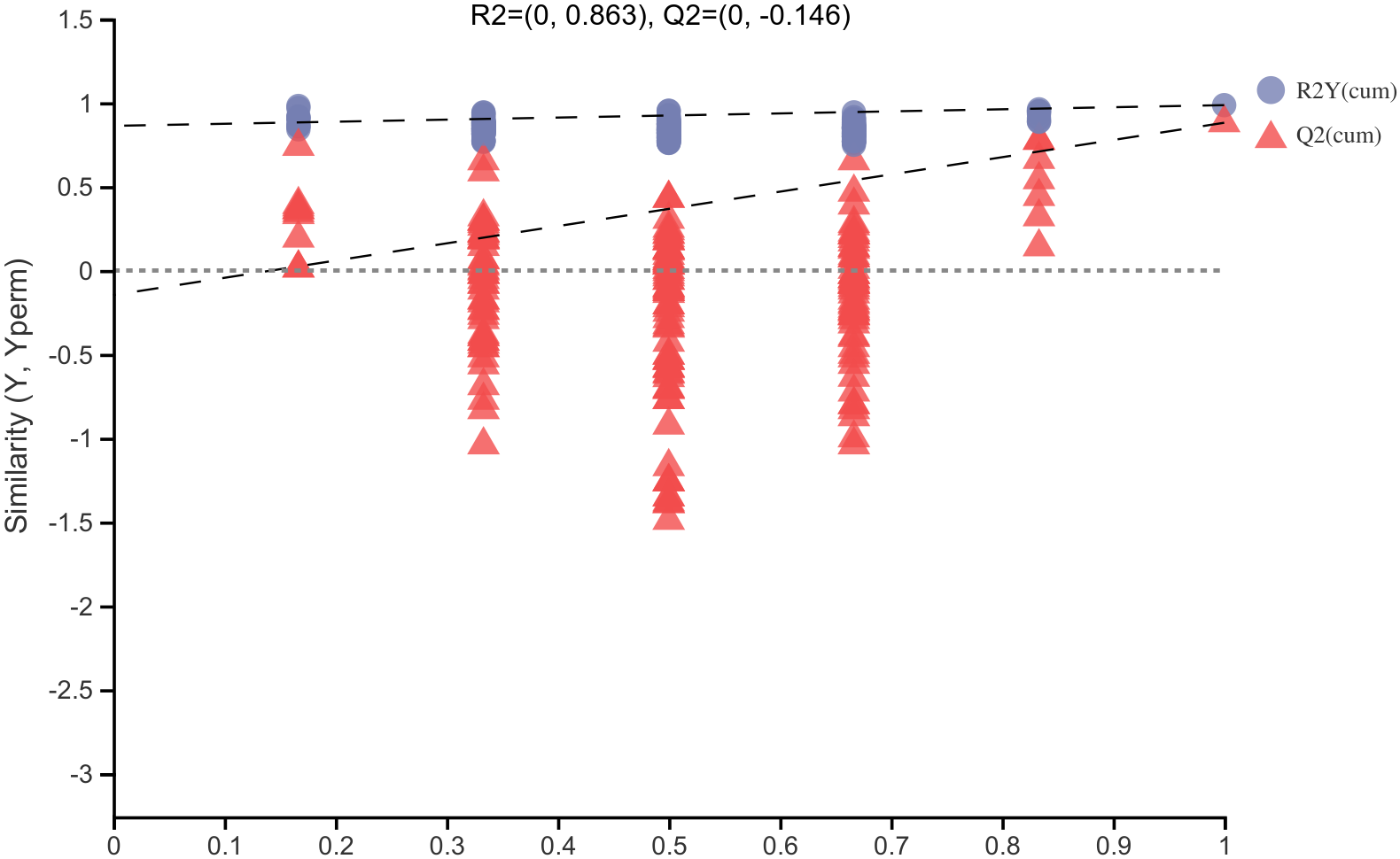

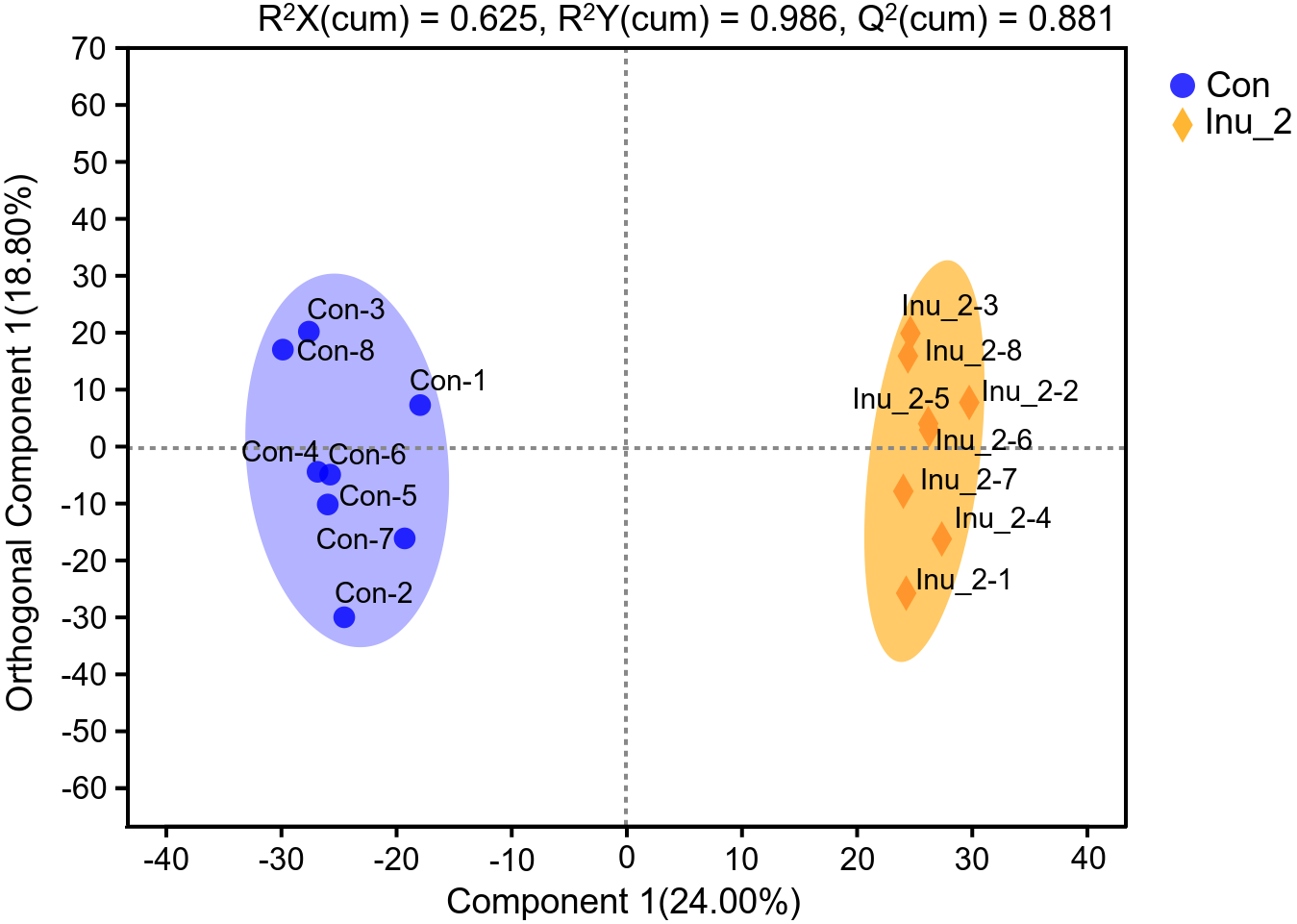


d

c

e


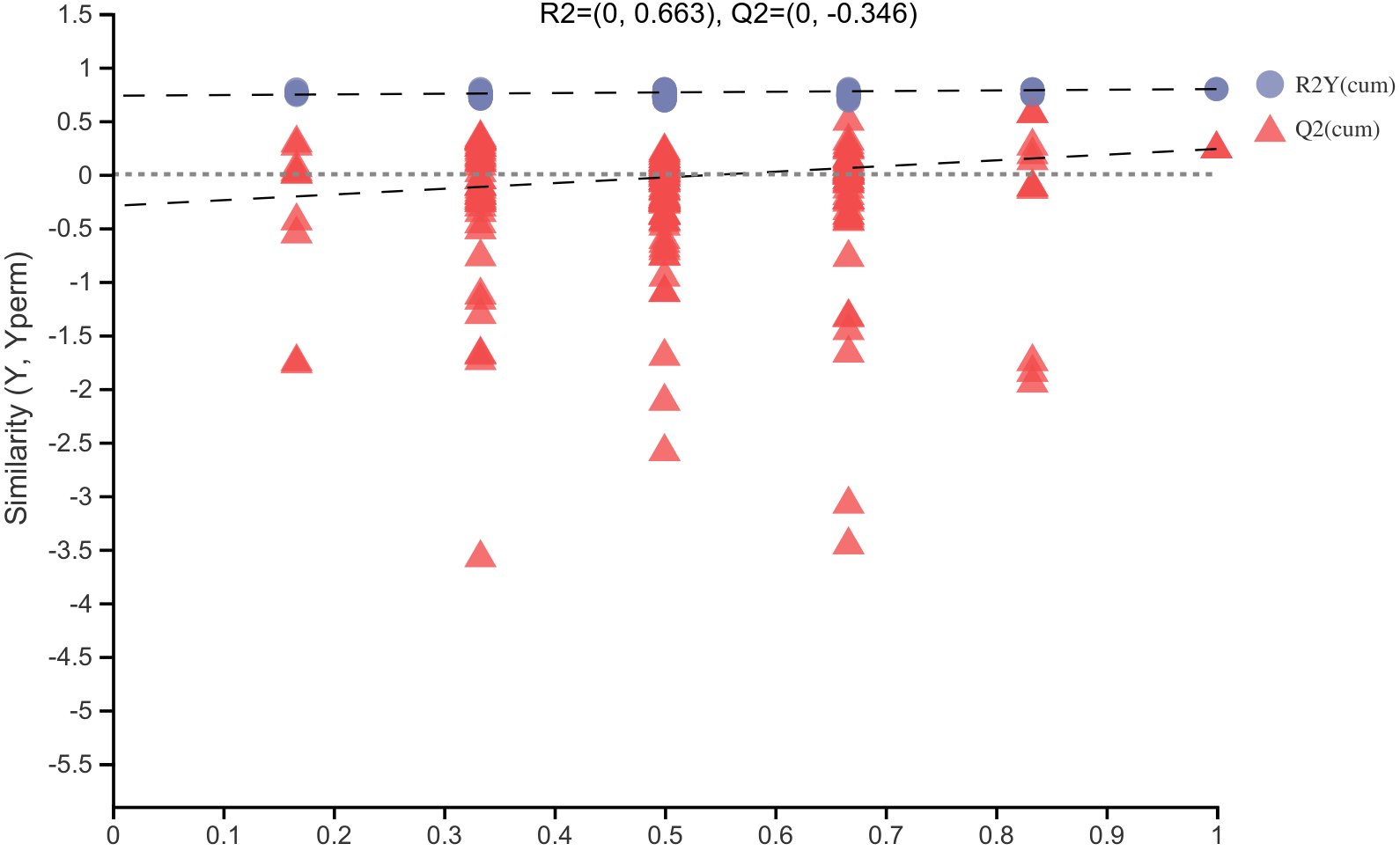

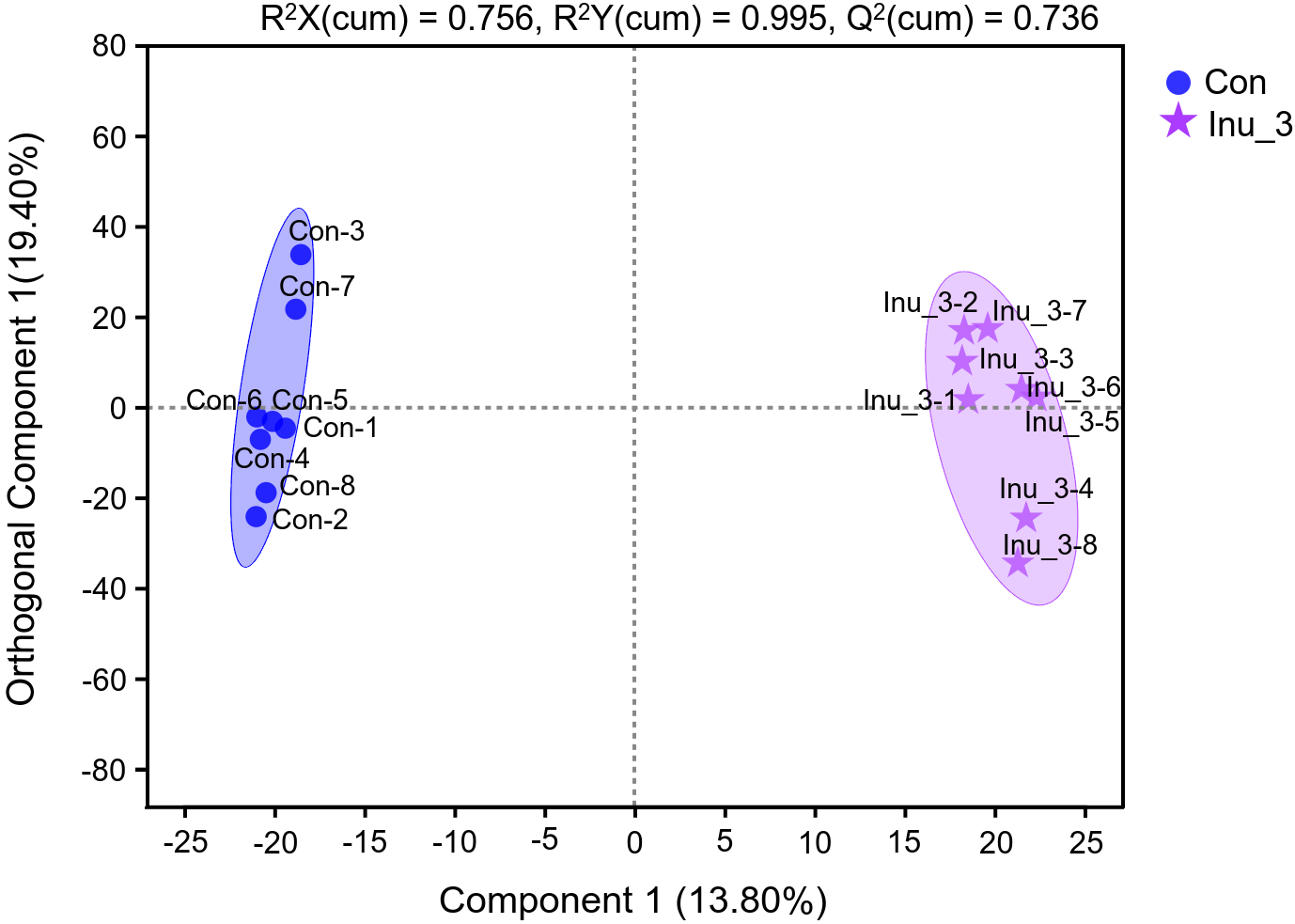


f

h

g


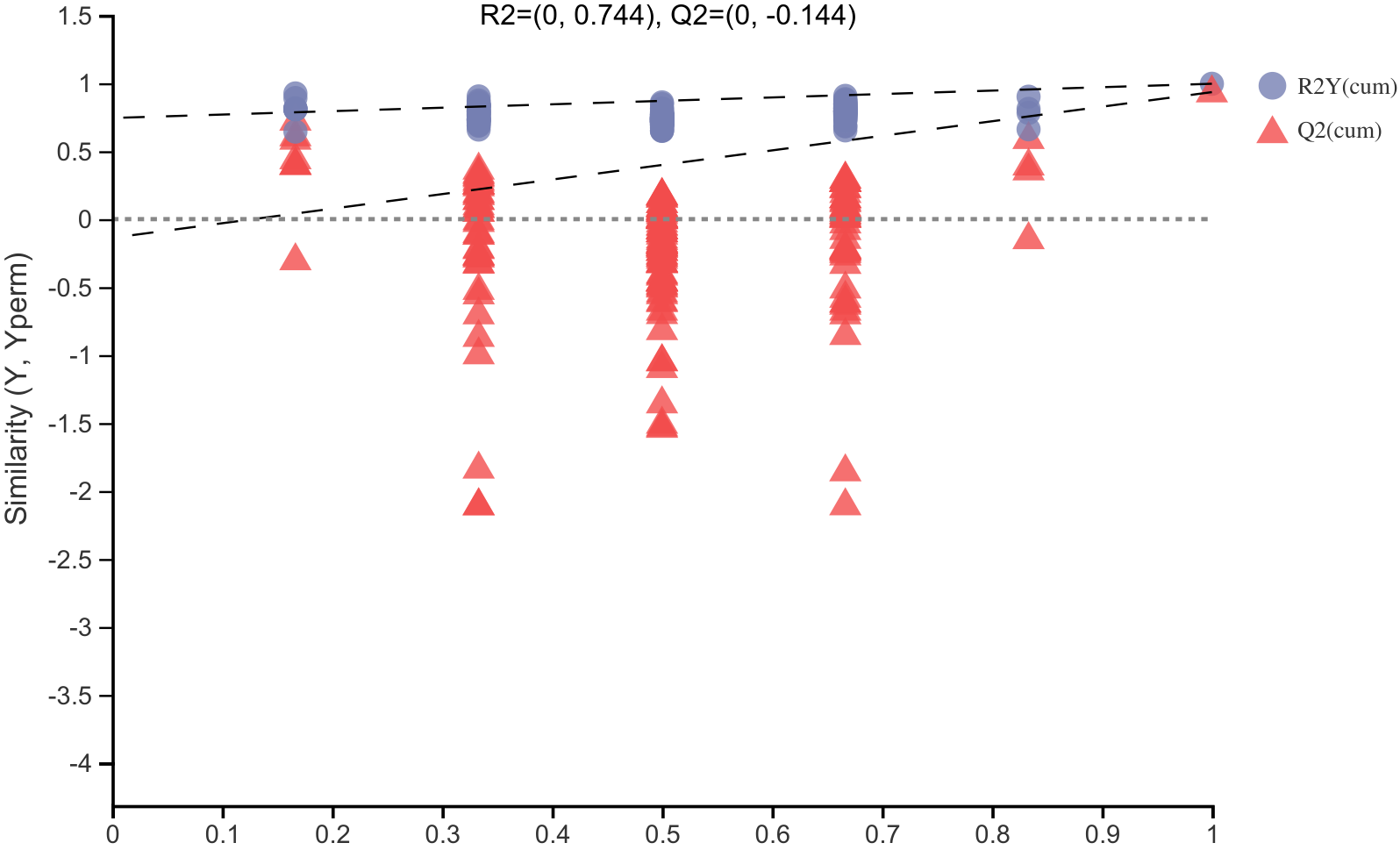

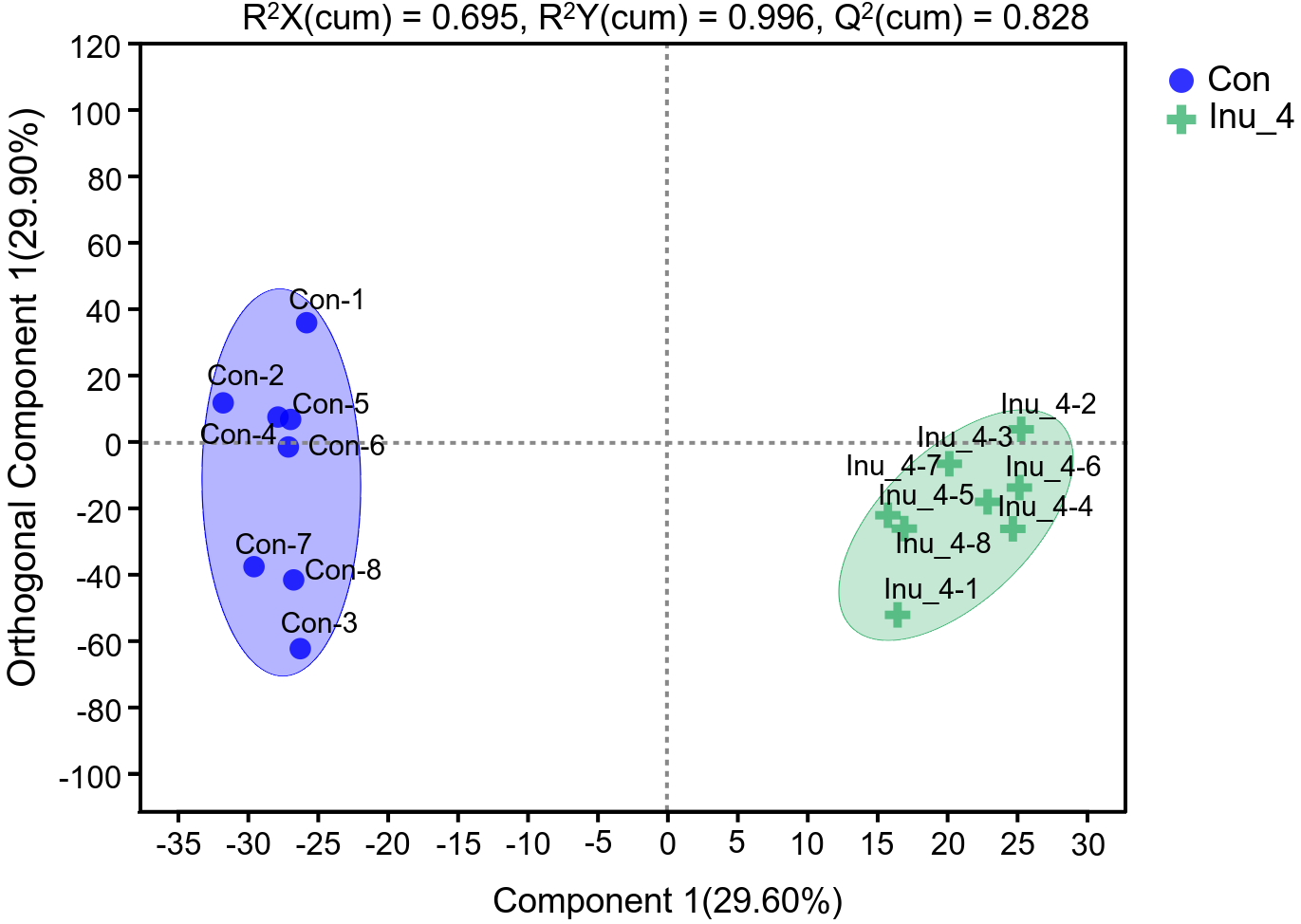


j

i


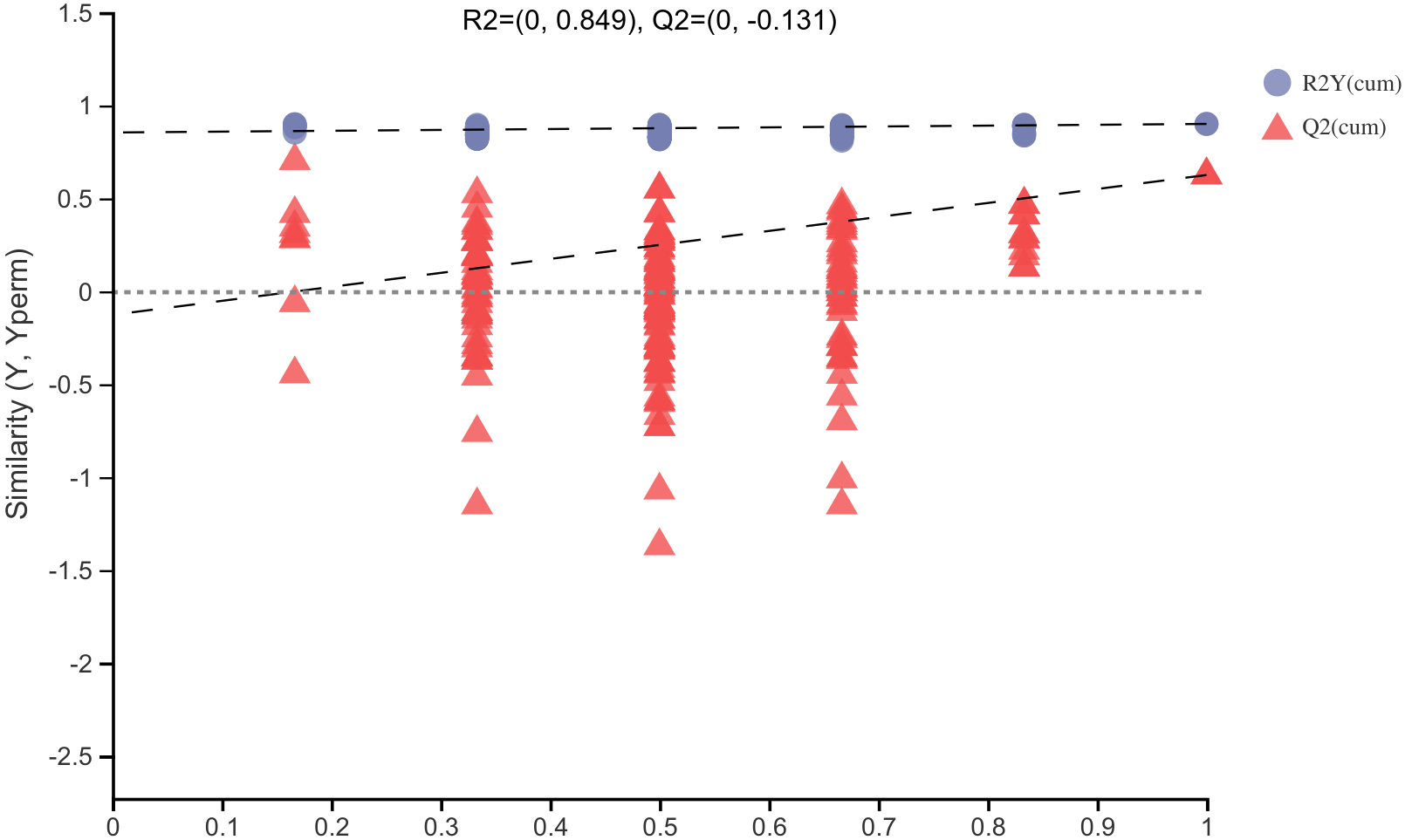

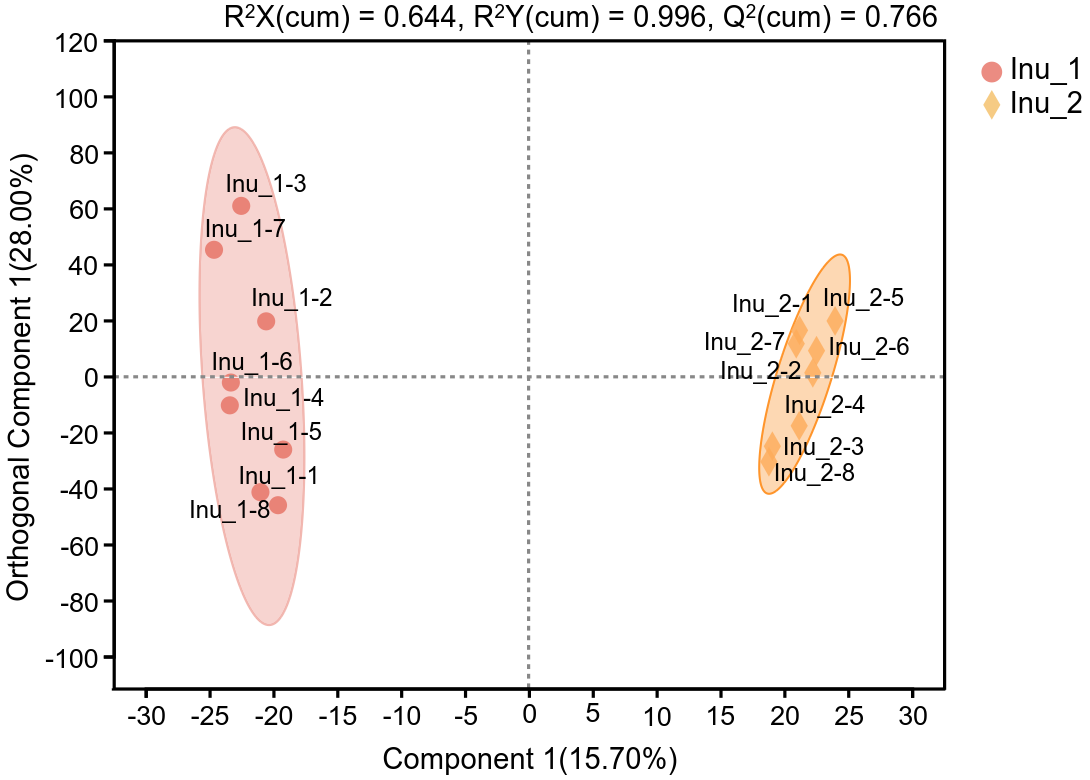


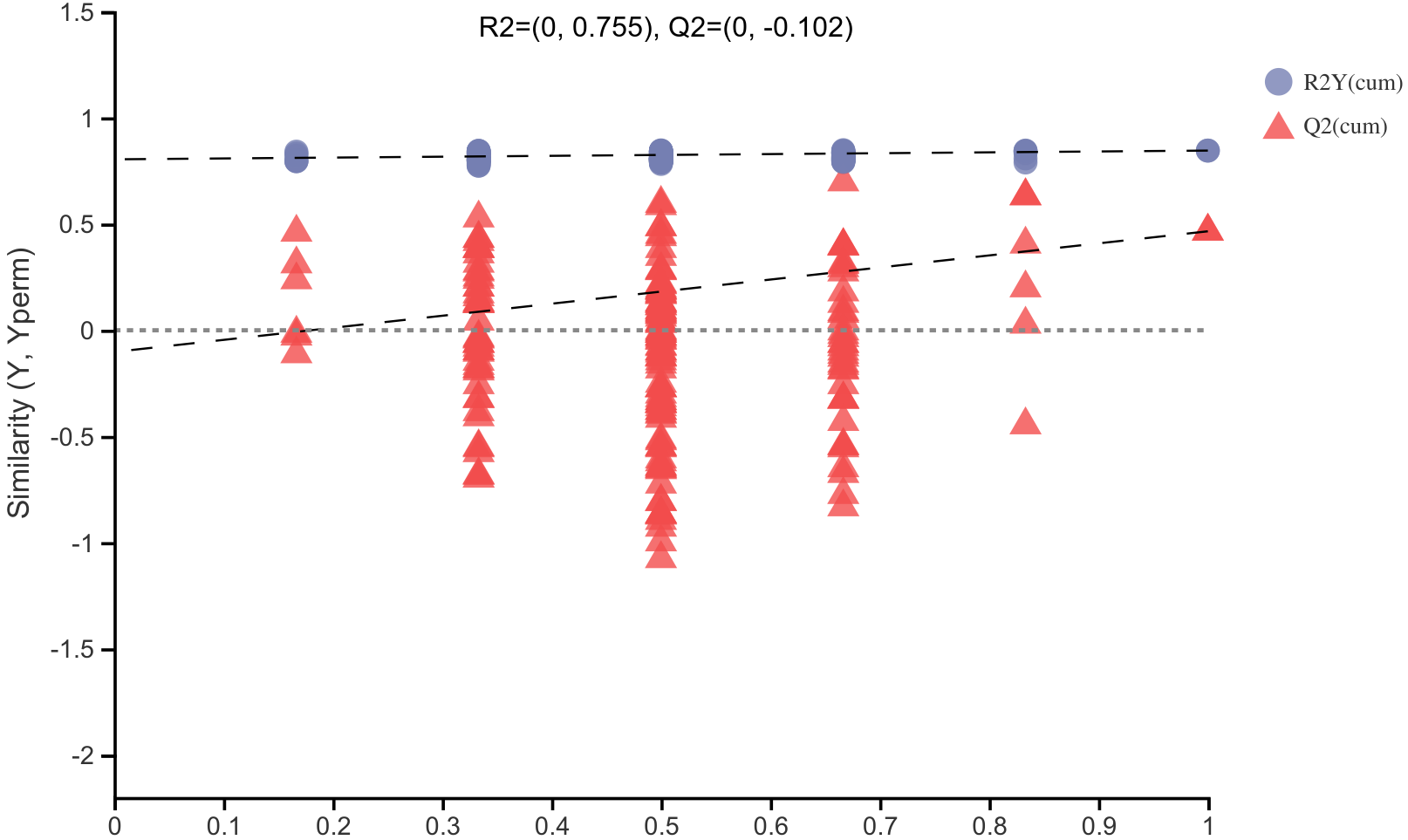

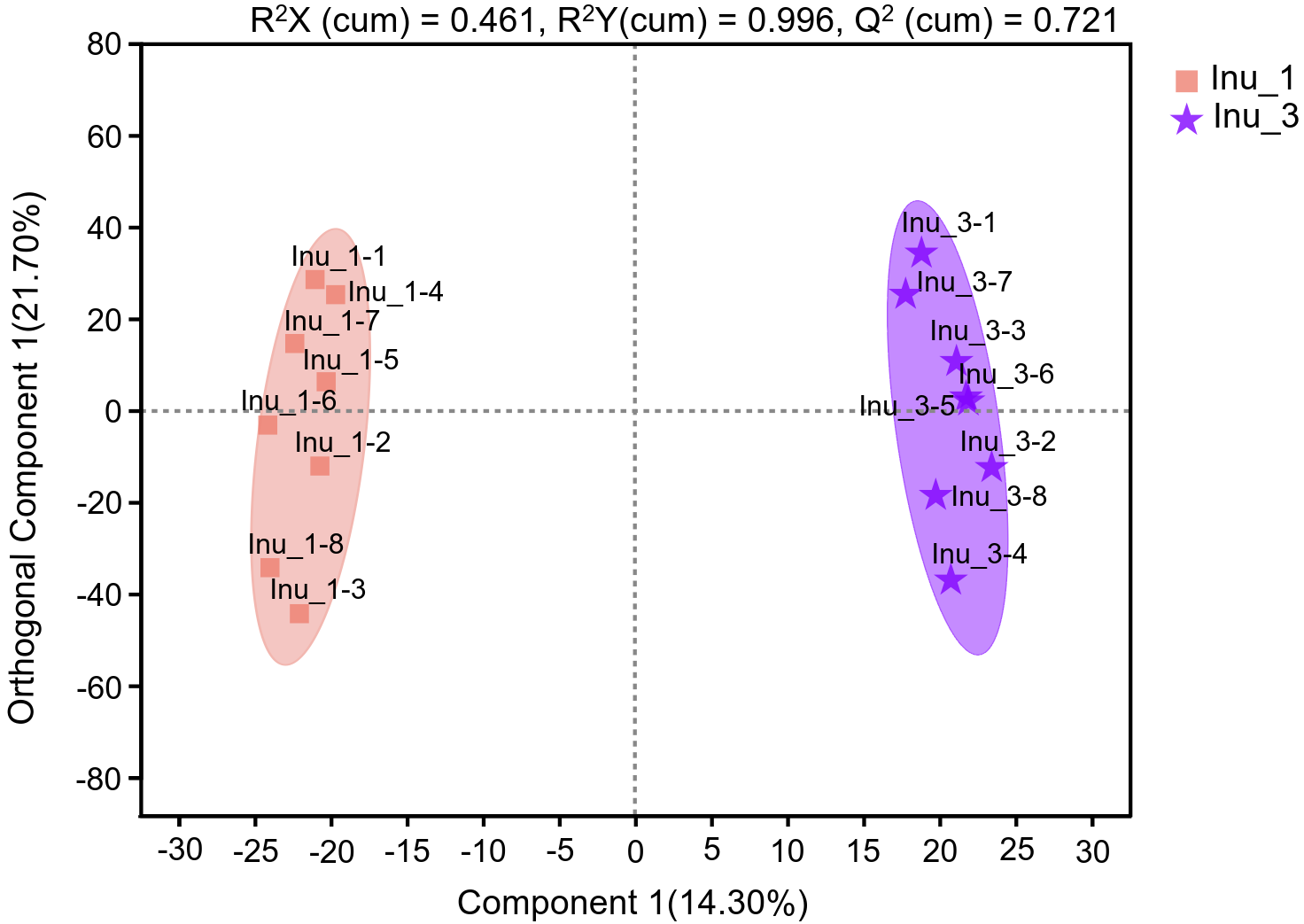


k

l

n

m


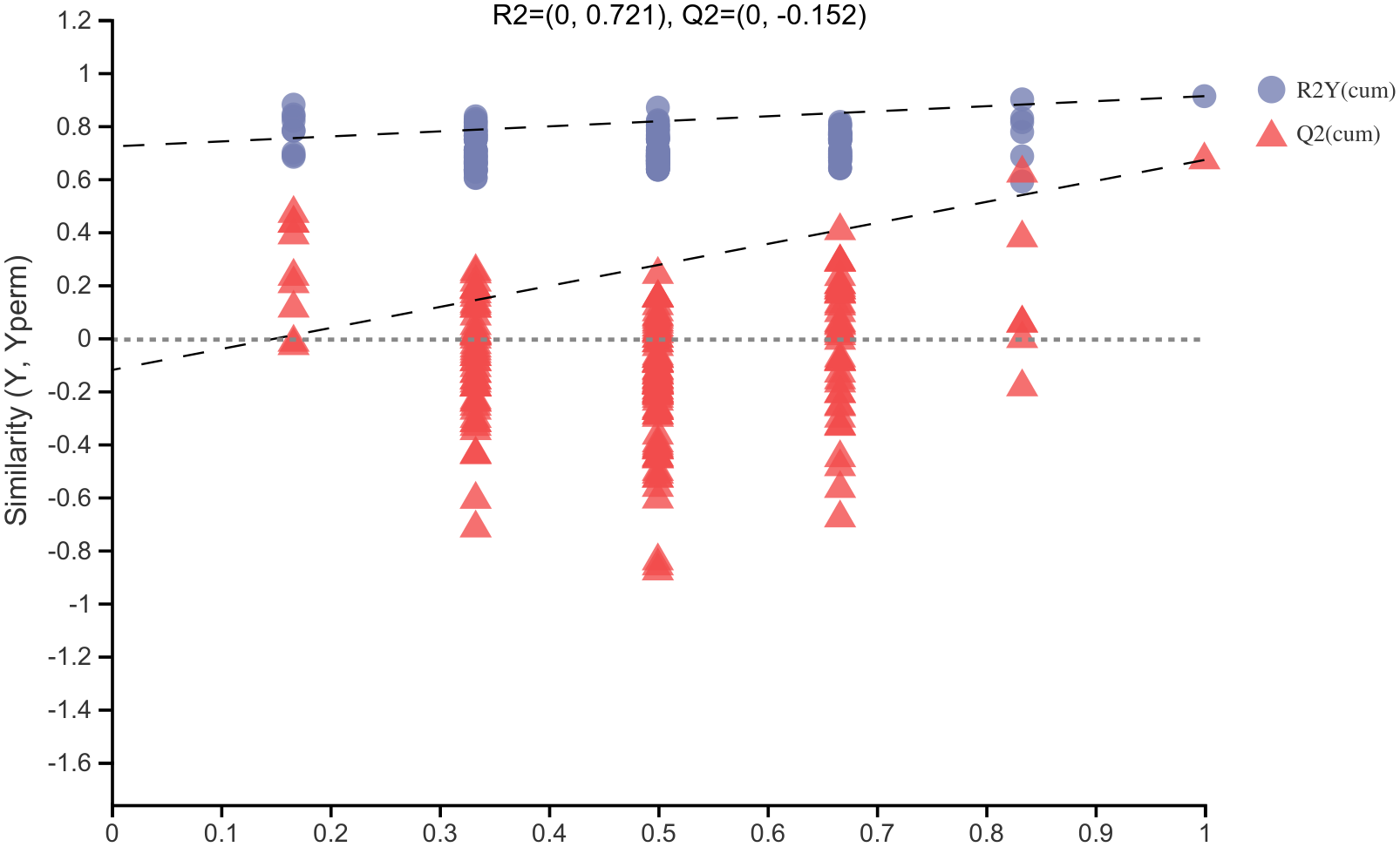

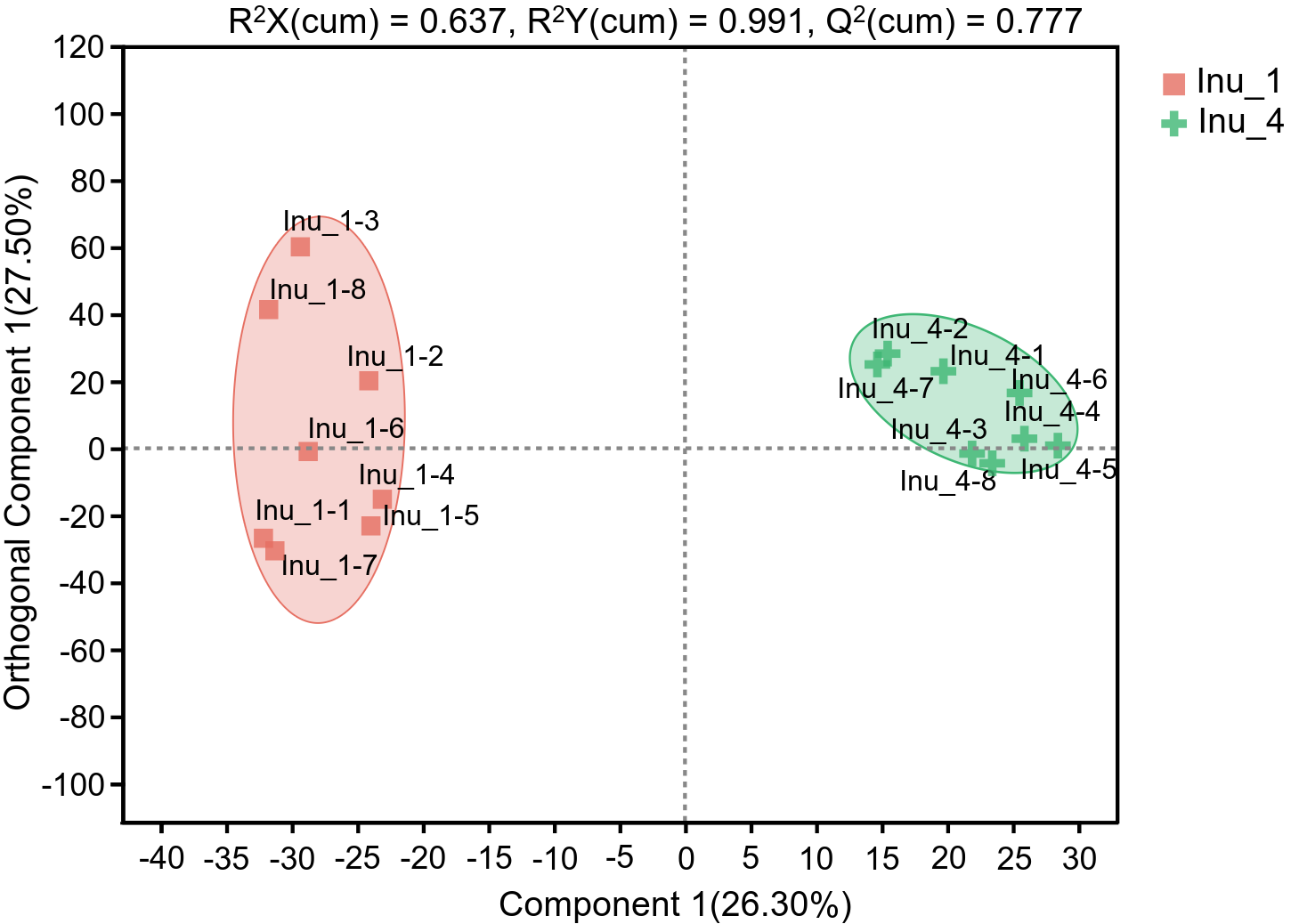


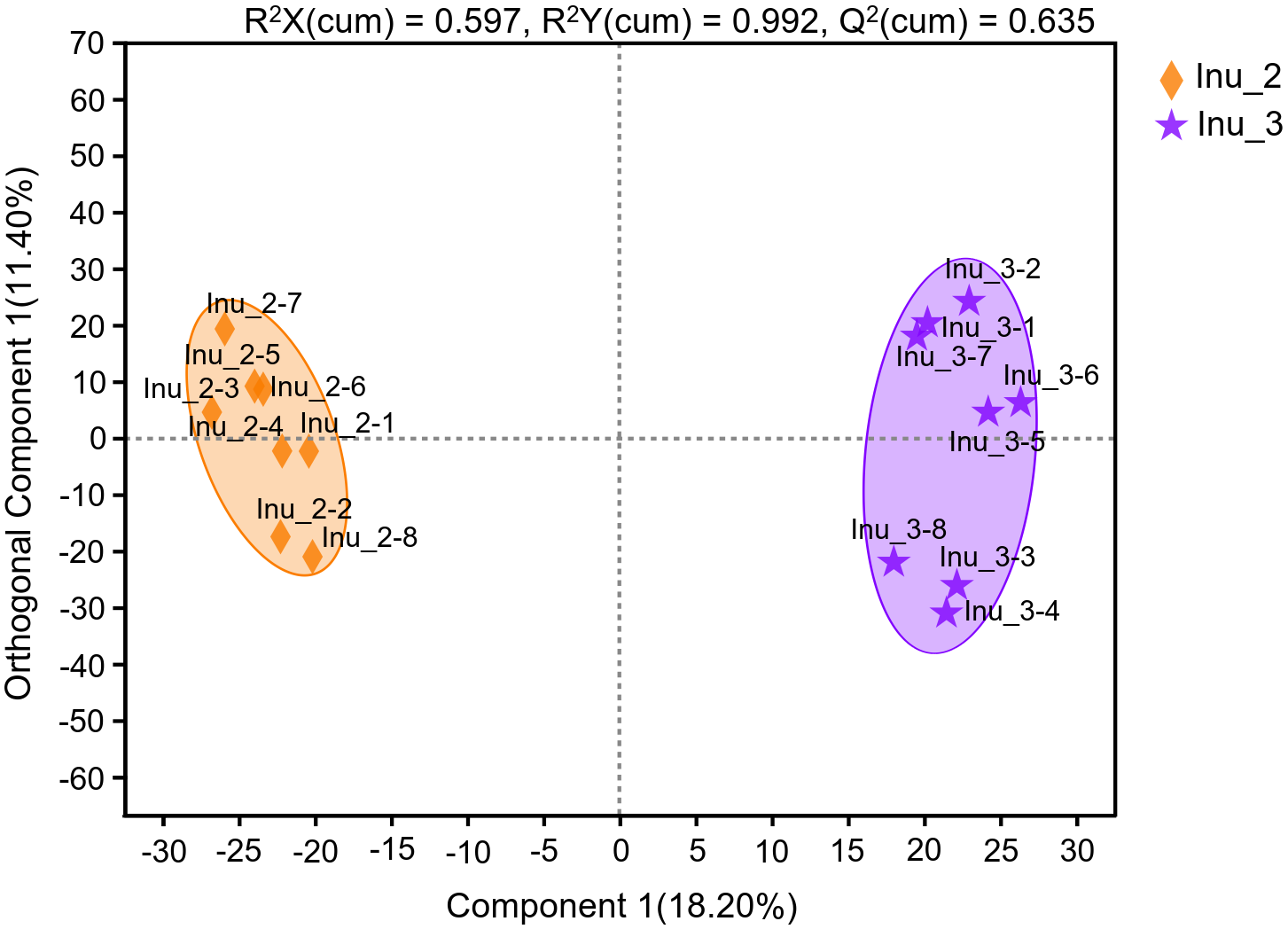


o

p


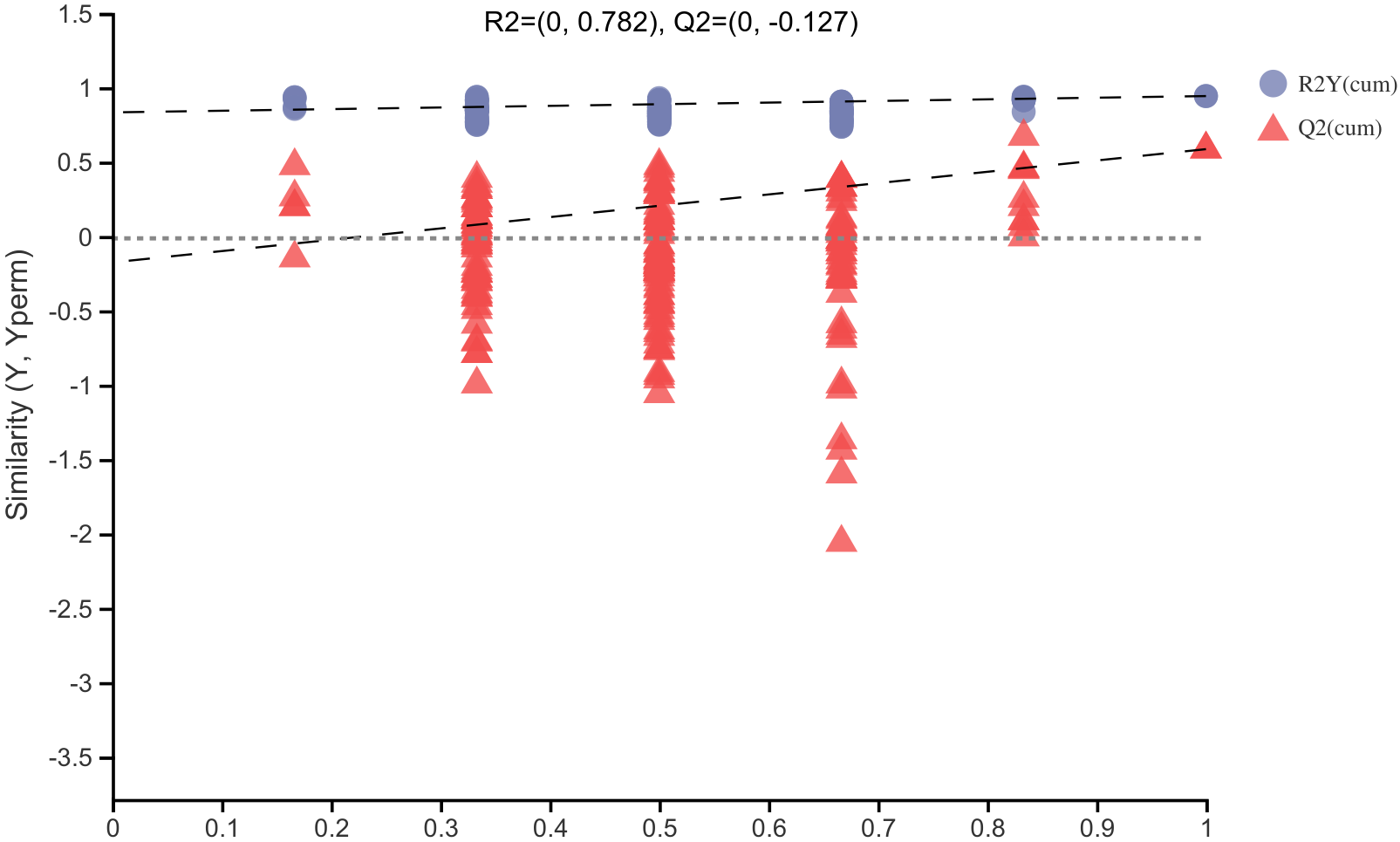


q

r


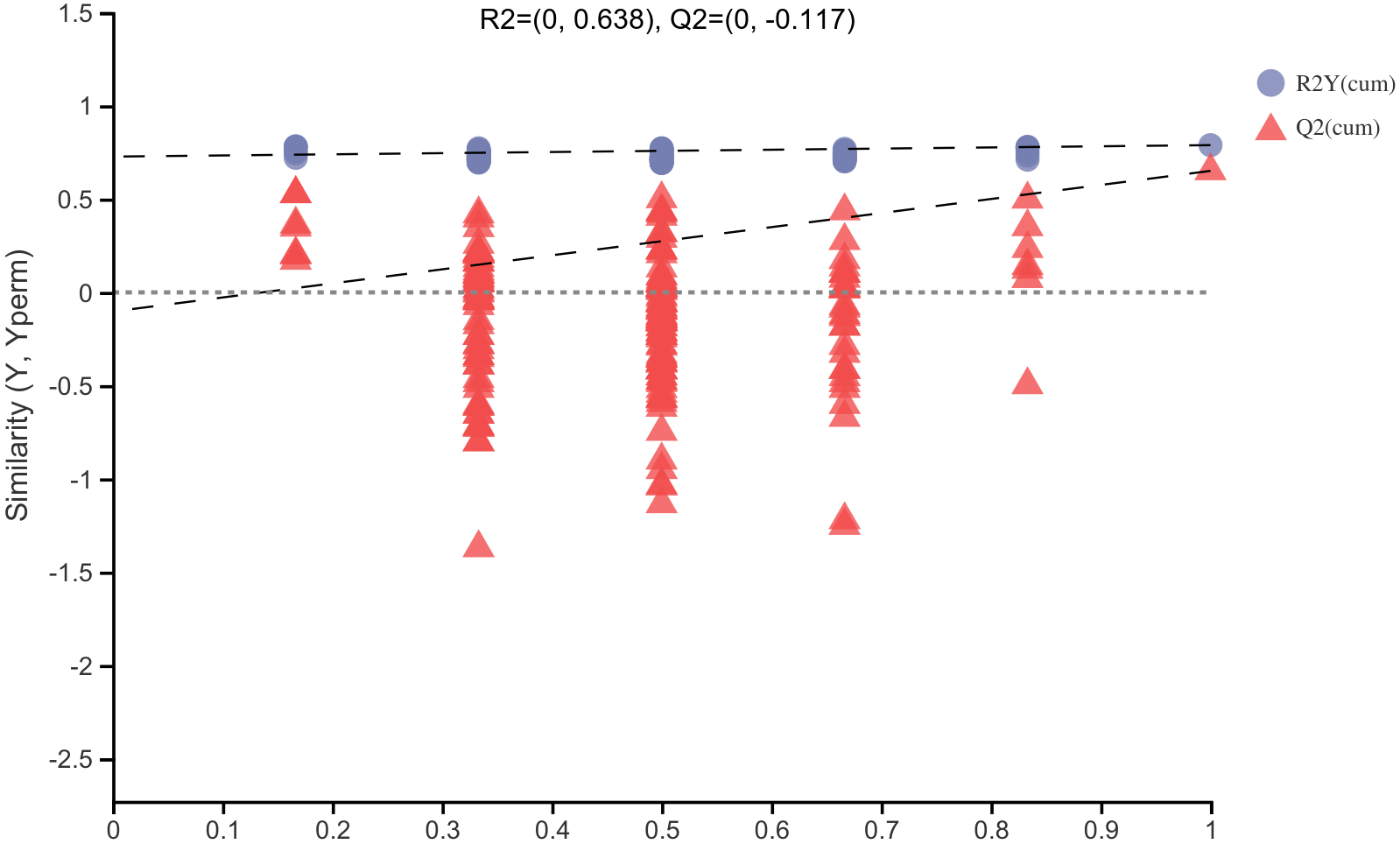

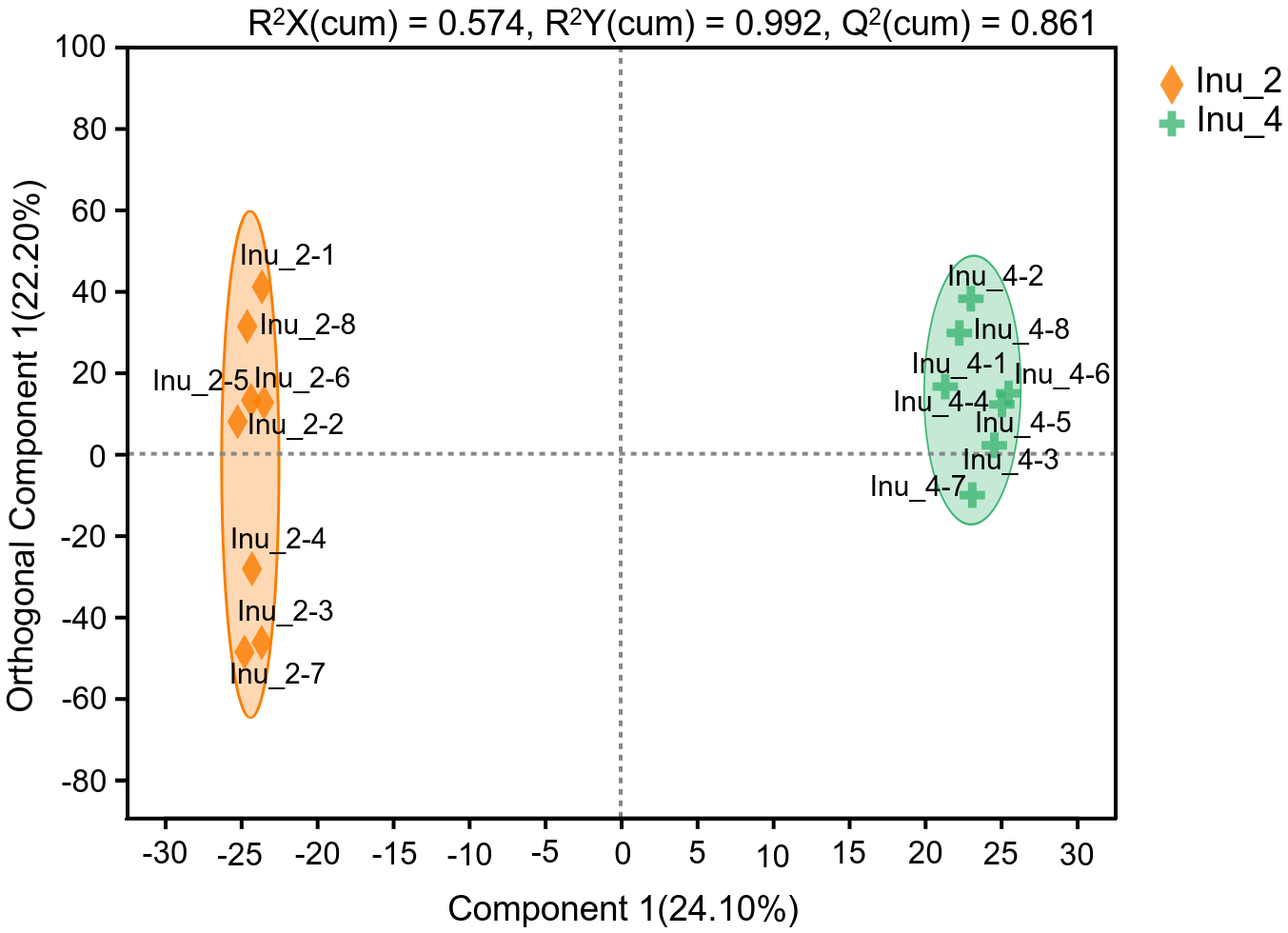


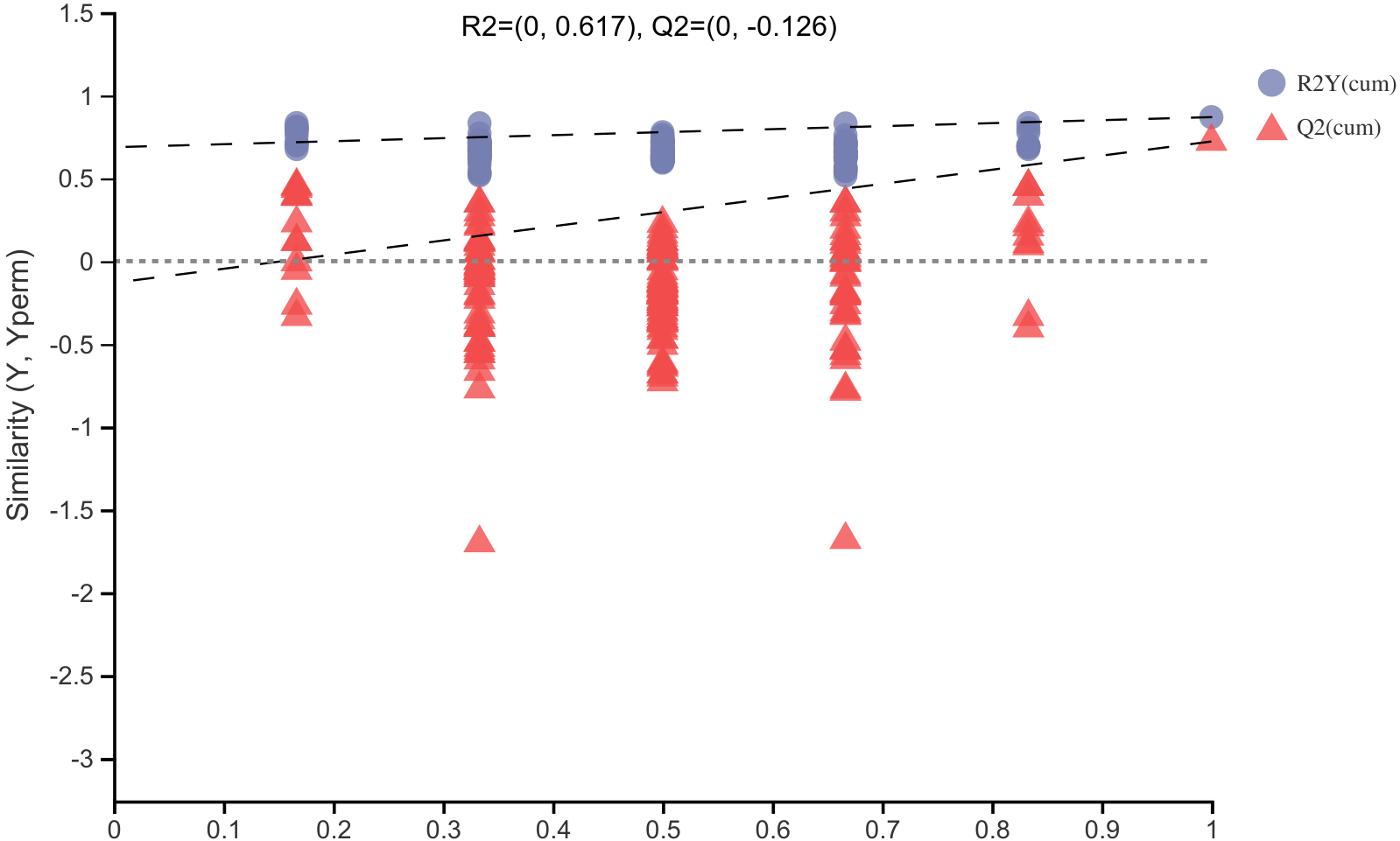

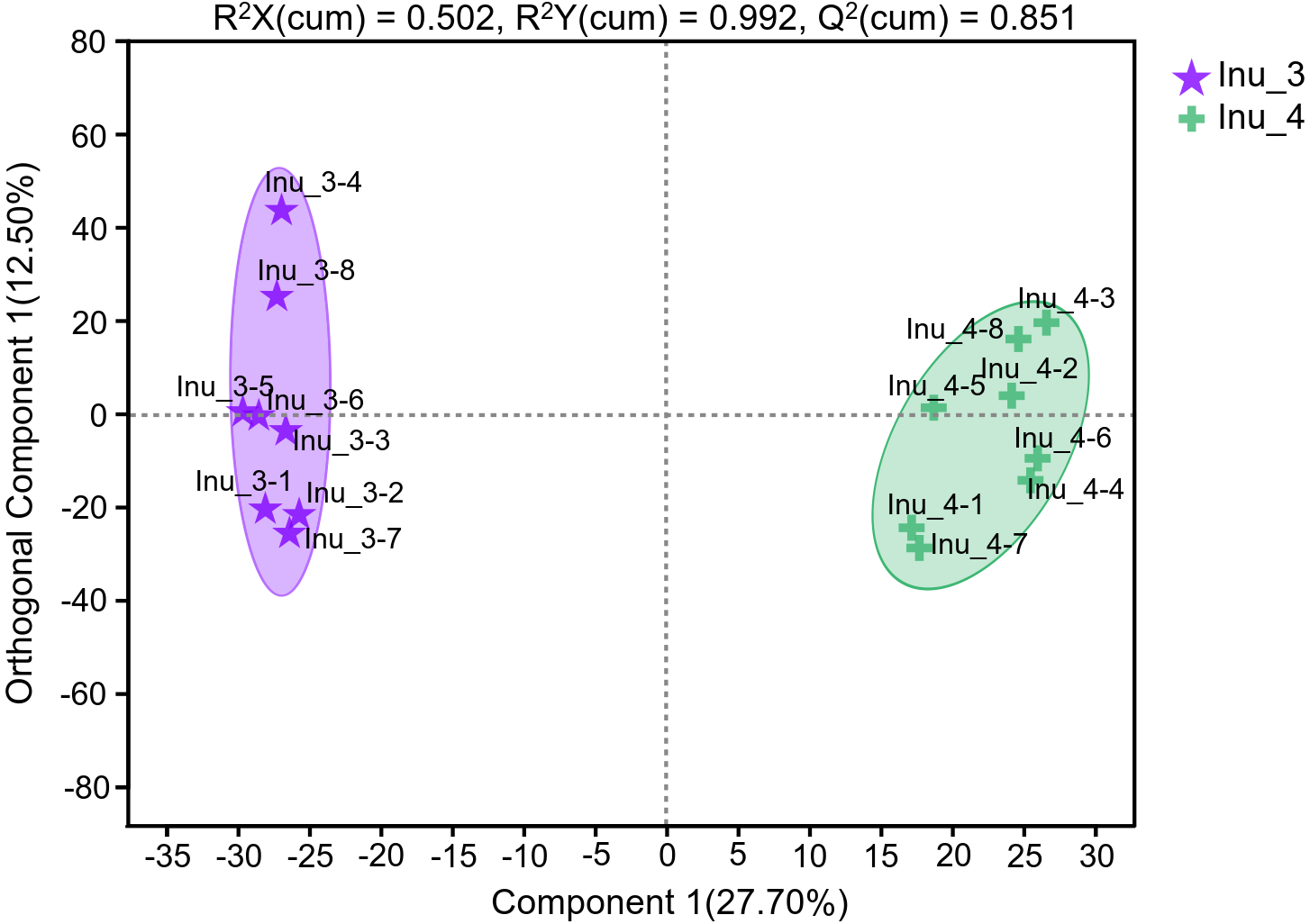


s

t


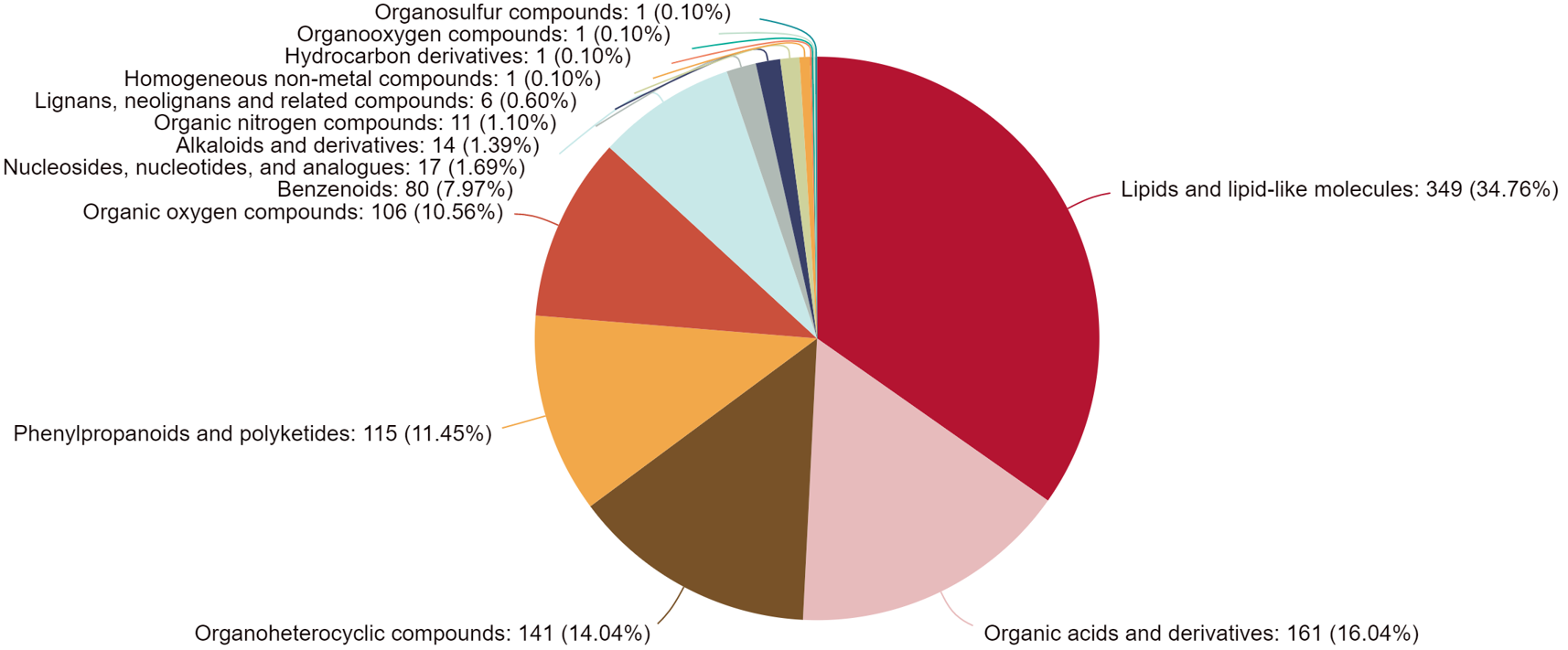

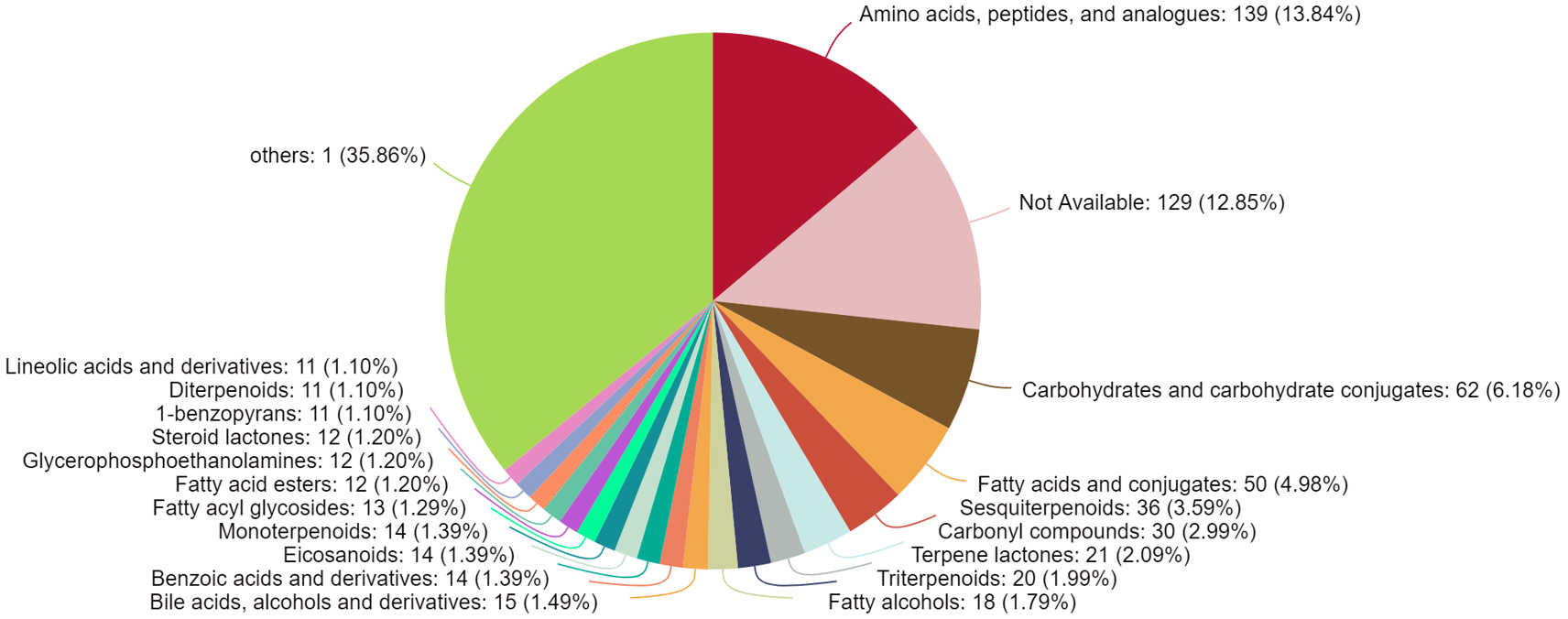
Figure S5 Orthogonal partial least squares discrimination analysis (OPLS-DA) score plot (A, C, E, G, I, K, M, O, Q, S) and OPLS-DA permutation testing (B, D, F, H, J, L, N, P, R, T) of serum metabolites in control and different inulin addition groups. R^2^X(cum) and R^2^Y(cum) represent the cumulative interpretation rate to the X and Y matrices of the model, respectively; Q^2^(cum) represents the predictive ability of the model. The closer these three indicators are to 1, the more stable and reliable the model is. Q^2^(cum) > 0.5 indicates that the predictive ability of the model is better. Con = control group; Inu_1 = inulin-1 group, the inulin addition level was 100 g/d per cow; Inu_2 = inulin-2 group, the inulin addition level was 200 g/d per cow; Inu_3 = inulin-3 group, the inulin addition level was 300 g/d per cow; Inu_4 = inulin-4 group, the inulin addition level was 400 g/d per cow.

a

b


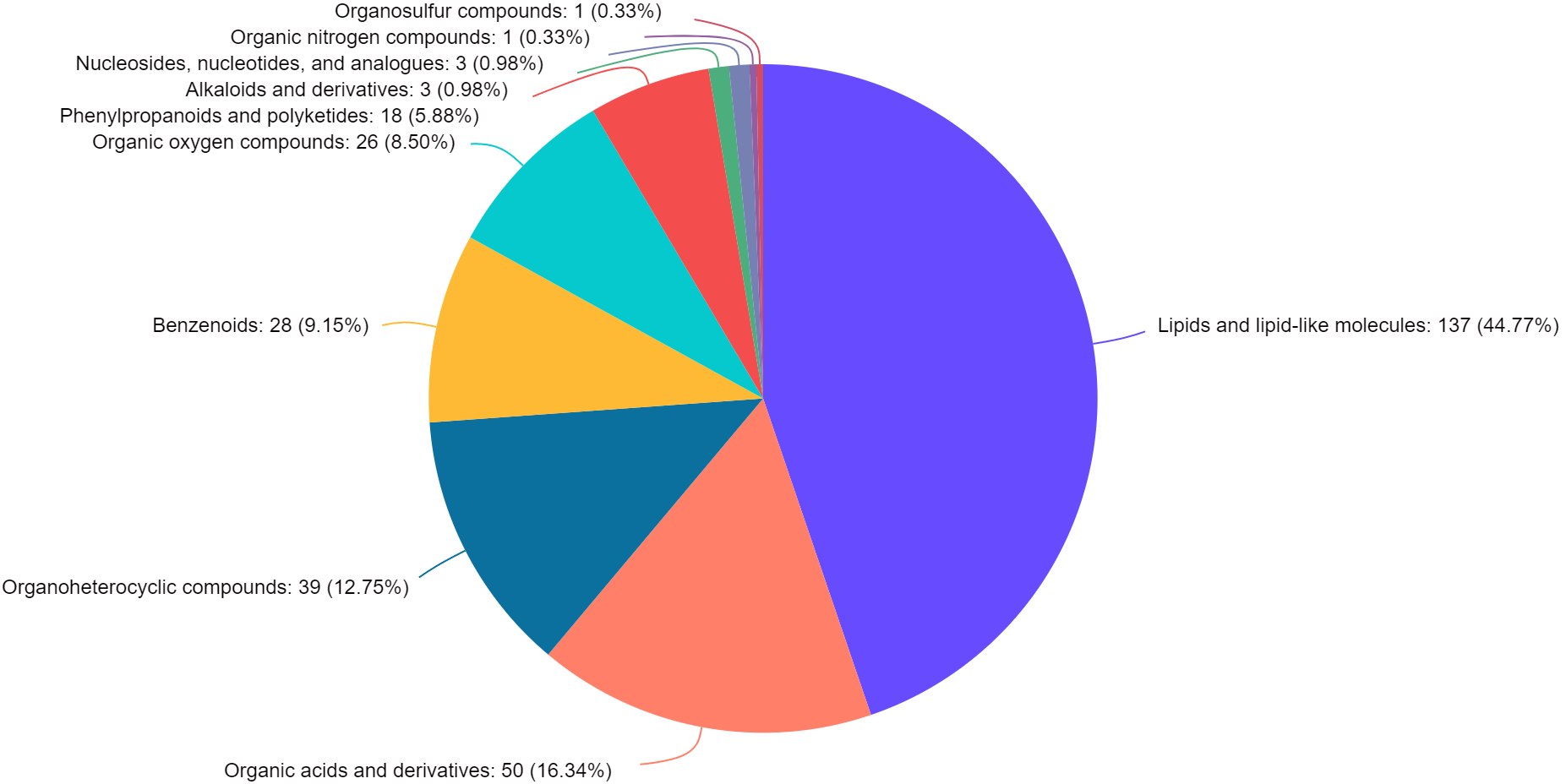


c

d


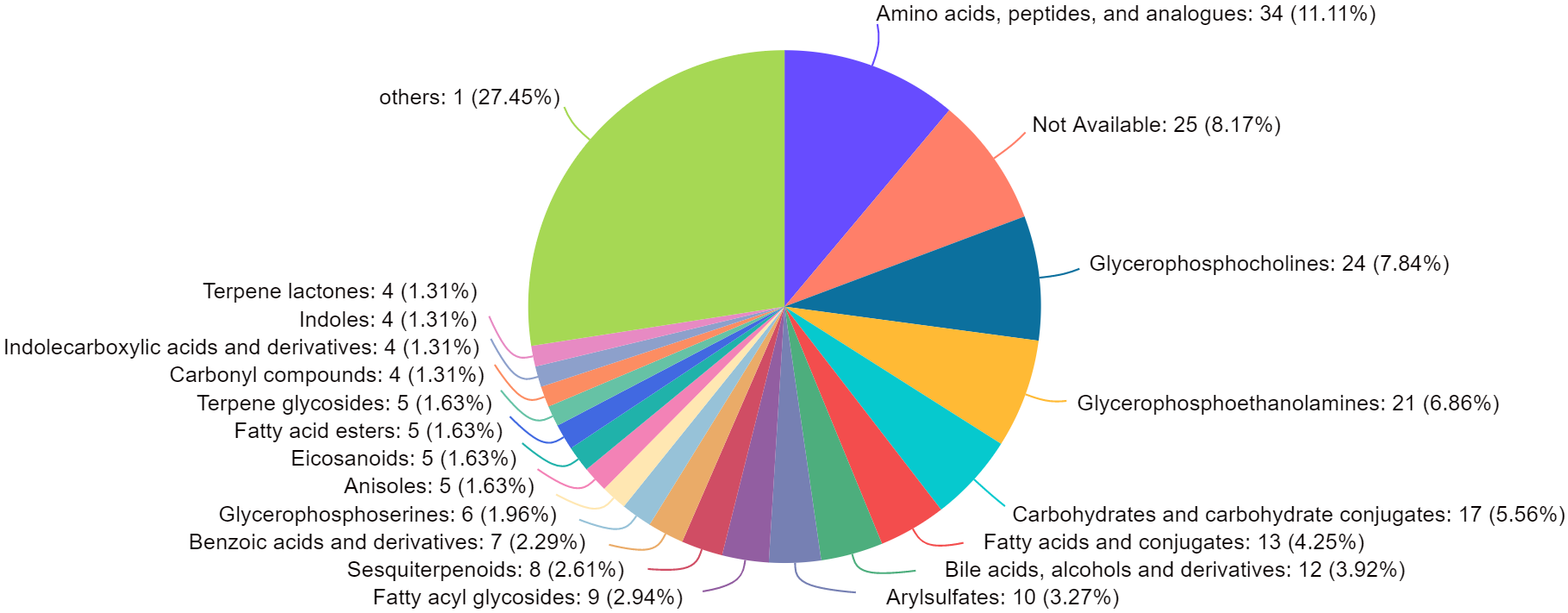


Figure S6 Classification of metabolites in (A and B) feces and (C and D) serum based on Human metabolome database (HMDB). (A and C) at superclass; (B and D) at subclass level.
